# Supplementary figures and images for: The ortholog of human ssDNA-binding protein SSBP3 influences neurodevelopment and autism-like behaviors in Drosophila melanogaster
Source: PLoS Biol. 2023 Jul 24;21(7):e3002210. doi: 10.1371/journal.pbio.3002210 (PMC10399856; doi:10.1371/journal.pbio.3002210)

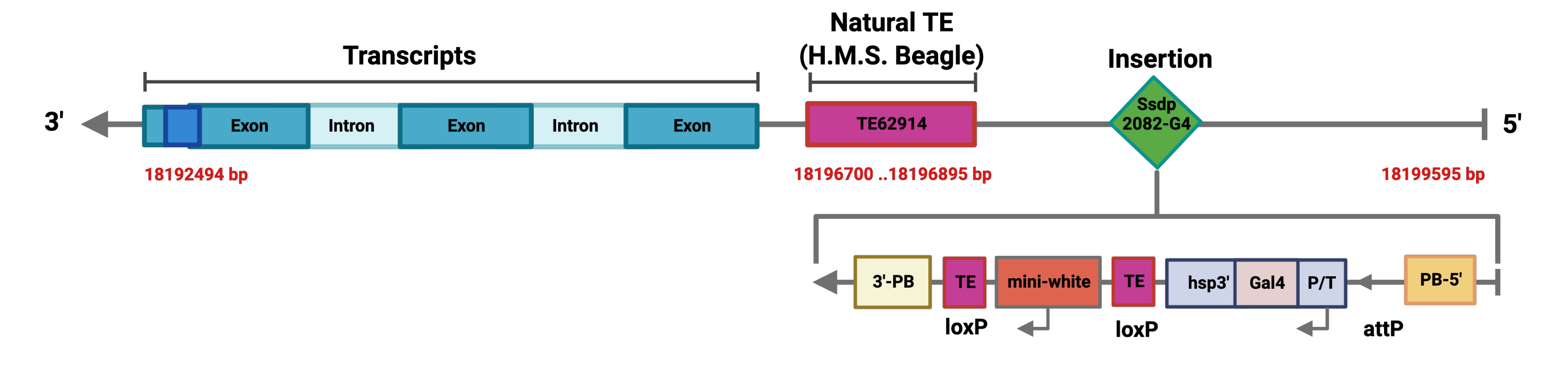

Supplement: S1 Fig — Mapped positions show presence of H.M.S. Beagle, a natural transposable elements (TE) as well as other TEs upstream of the Ssdp[2082-G4]-Gal4 insertion, which may enhance the transcription of nearby loci. Created with BioRender.com. (TIF) [file pbio.3002210.s001.tif]

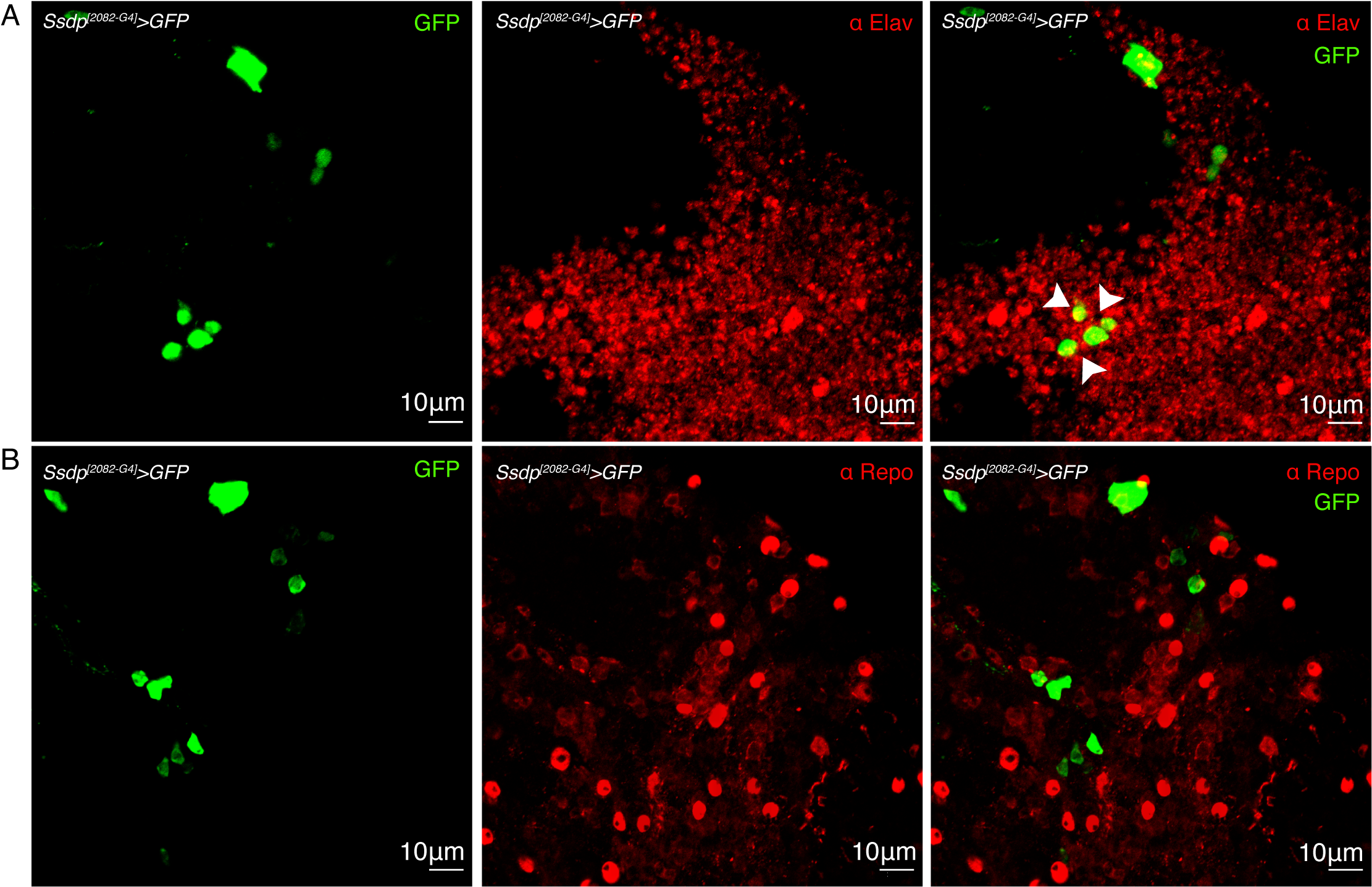

Supplement: S2 Fig — (A and B) 100× magnification images showing expression pattern of 6x-UAS-GFP driven by Ssdp[2082-G4]-Gal4 in the SLP region with anti-Elav and anti-Repo staining, respectively. White arrows point towards co-localization with Elav-positive neurons in yellow. Scale bar = 10 μm. (TIF) [file pbio.3002210.s002.tif]

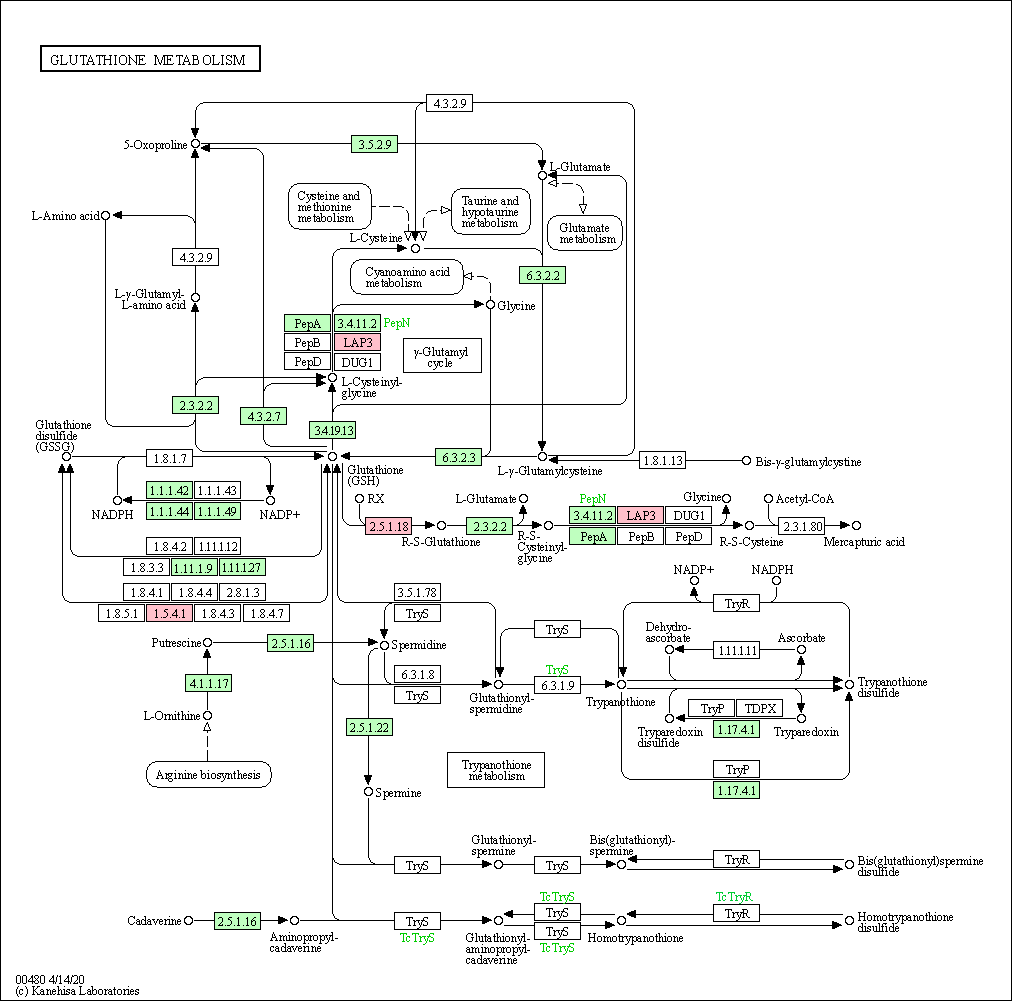

Supplement: S3 Fig — (TIF) [file pbio.3002210.s003.tif]

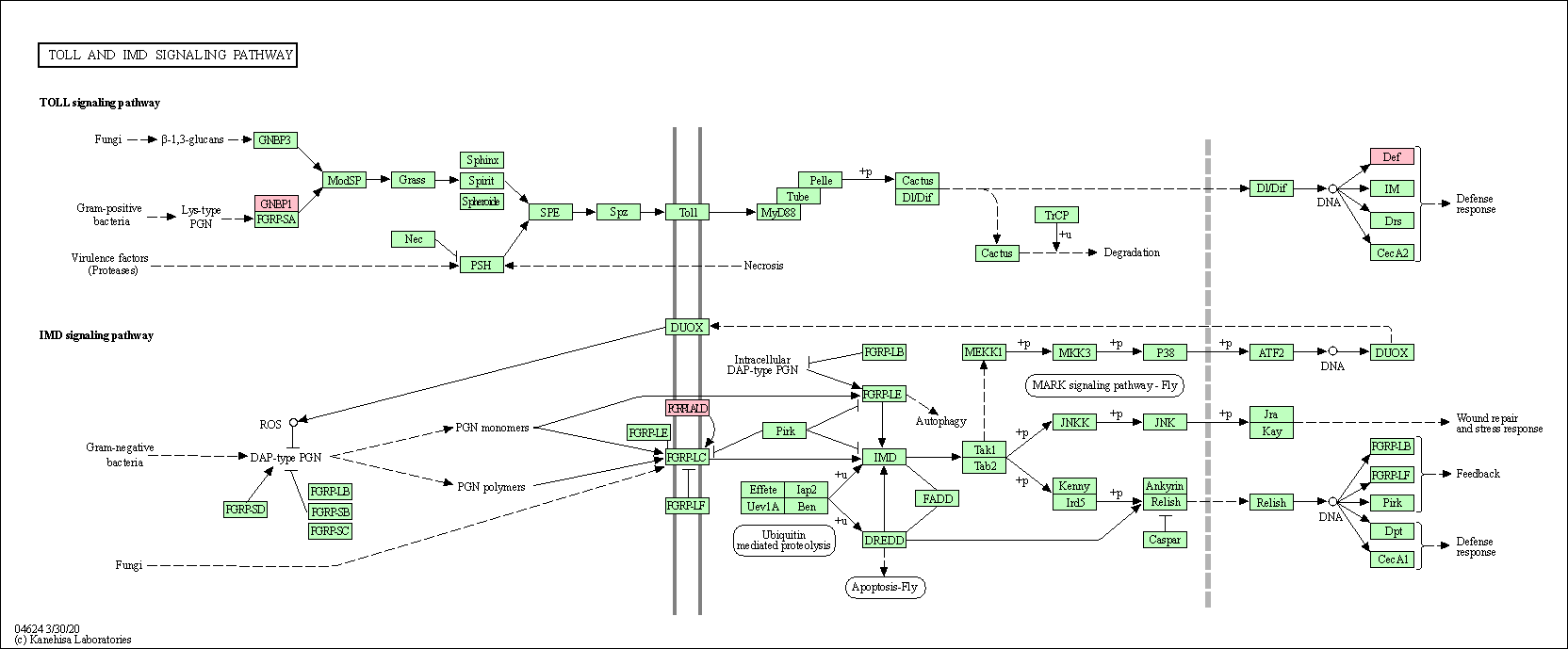

Supplement: S4 Fig — (TIF) [file pbio.3002210.s004.tif]

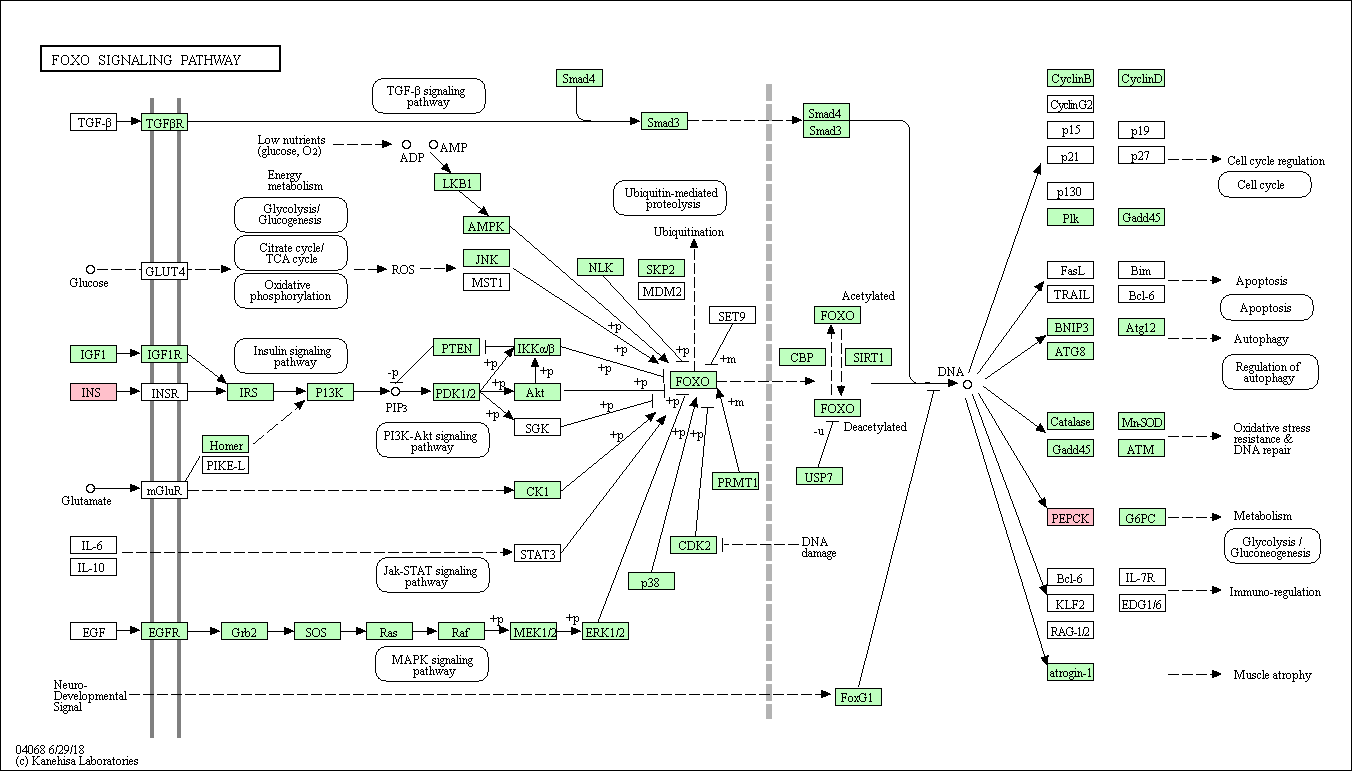

Supplement: S5 Fig — (TIF) [file pbio.3002210.s005.tif]

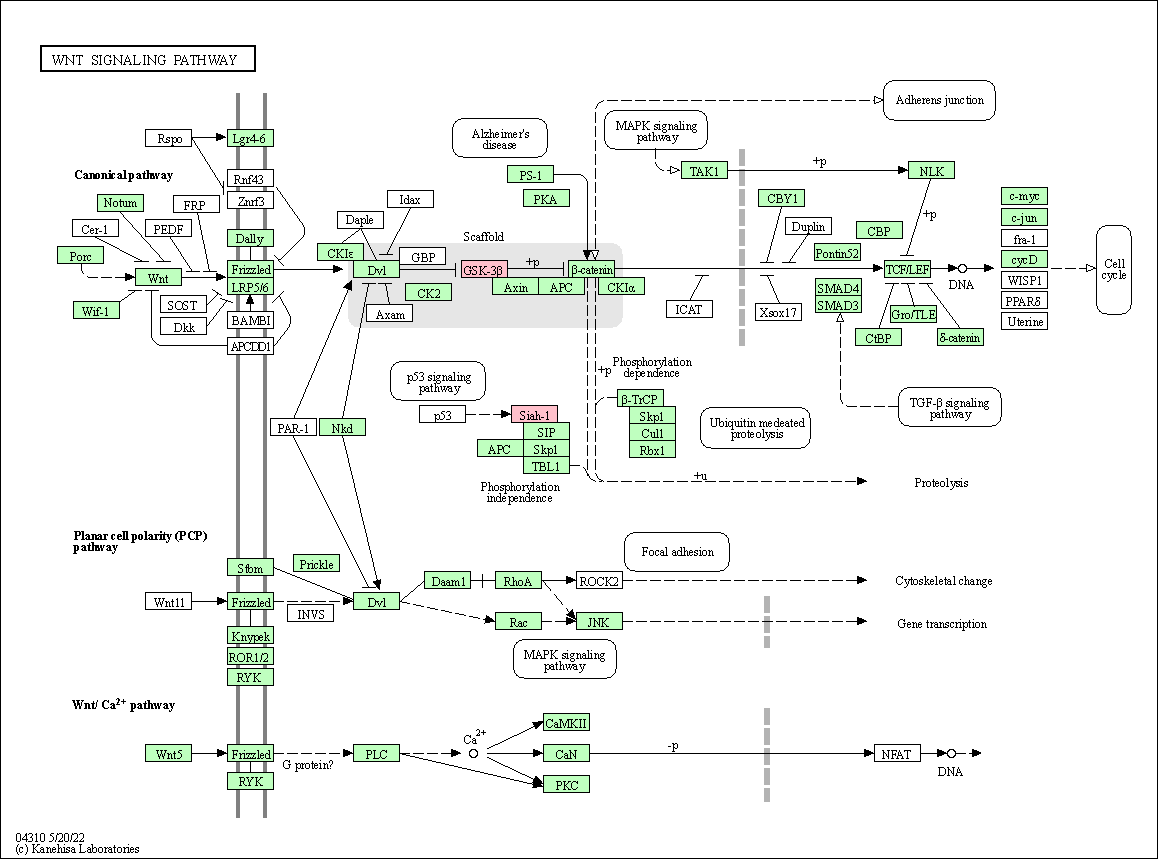

Supplement: S6 Fig — (TIF) [file pbio.3002210.s006.tif]

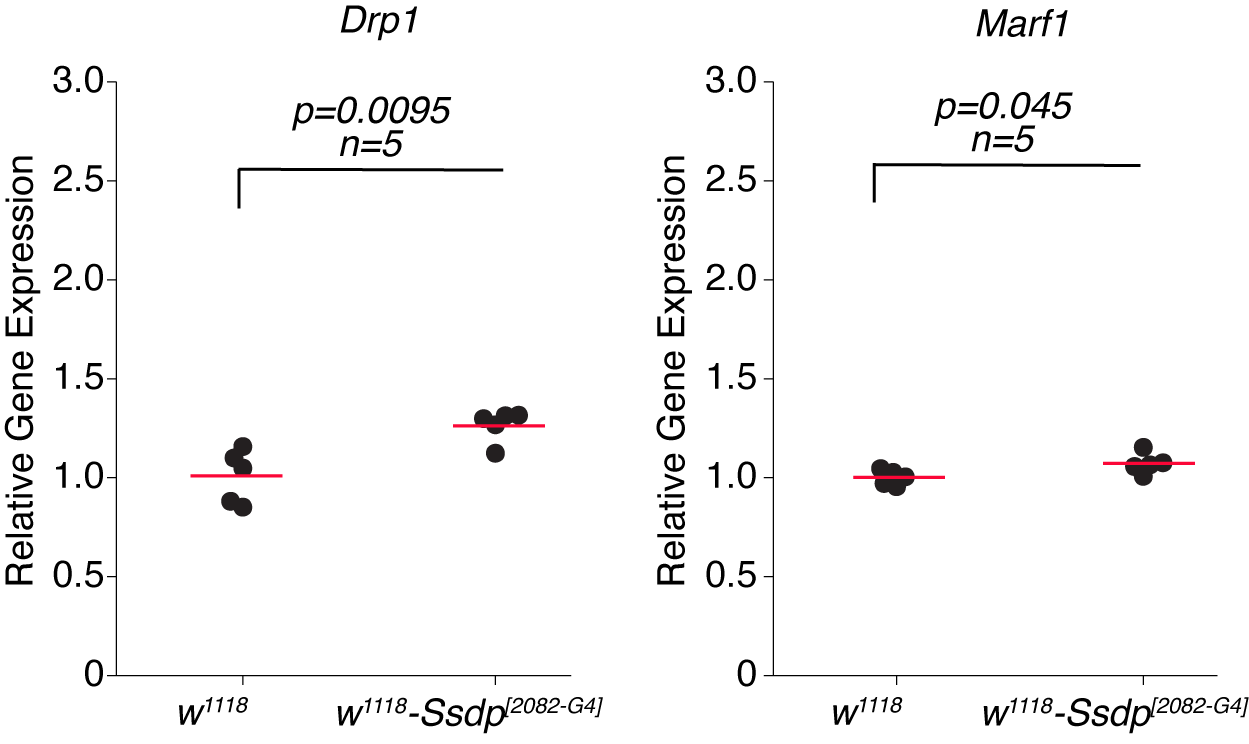

Supplement: S7 Fig — Both Drp1 (p = 0.0095, n = 5) and Marf1 (p = 0.045, n = 5) levels are significantly up-regulated in the brains of Ssdp[2082-G4]/+ flies compared to w1118 controls. P-values were obtained by Student’s t test. For all qRT-PCR experiments, expression was normalized to Rpl32. Horizontal red line represents the mean value. Each black dot is a data point representing an independent biological replicate with 20 individual fly heads per biological replicate. The raw data underlying this figure can be found in S1 Data file. (TIF) [file pbio.3002210.s007.tif]

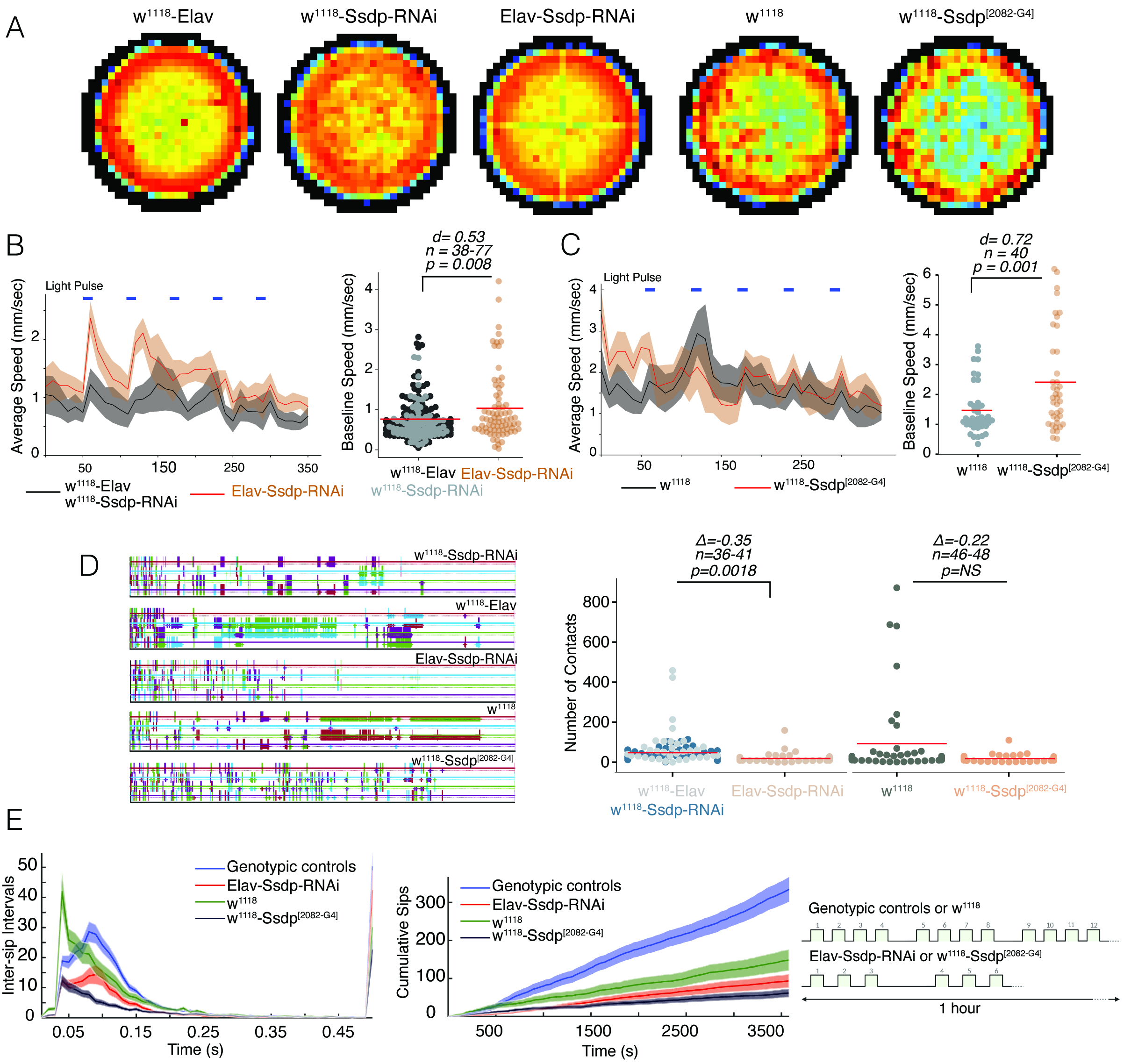

Supplement: S8 Fig — (A) Heatmap occupancy plots of wall-following behavior in an open field were averaged from the recorded behavior of all flies in each group; blue indicates the lowest occupancy in that region, and red indicates maximum occupancy. (B) Elav-Gal4>UAS-Ssdp-RNAi exhibit a heightened baseline locomotor speed (n = 38–77, Cohen’s d = 0.53 [95 CI 0.102, 0.985], p = 0.008) and startle to the first blue pulse than w1118>Elav-Gal4 and w1118-Ssdp-RNAi controls. (C) Heterozygous Ssdp[2082]/+ flies also show increased baseline locomotor speed (n = 40, Cohen’s d = 0.72 [95 CI 0.298, 1.09], p = 0.001) and startle to the first blue pulse compared to w1118 controls. (D) History of sitting fly interactions represents an interactogram for 20 min for each genotype. Each vertical line’s thickness represents time spent interacting with another fly (left). Number of direct social contacts between flies in a group are reduced in both Elav-Gal4>UAS-Ssdp-RNAi (n = 36–41, Cliff’s Δ = −0.35 [95 CI −0.55, −0.13], p = 0.0018) and Ssdp[2082]/+ flies (n = 46–48, Cliff’s Δ = −0.22 [95 CI −0.44, 0.016], p = 0.067) compared to controls (right); however, for Ssdp[2082]/+ flies the decrease is not statistically significant. (E) Histogram depicts reduced inter-sip intervals in Elav-Gal4>UAS-Ssdp-RNAi and Ssdp[2082]/+ flies (n = 35–45) (left). Line plots show cumulative sips over the assay time, which are decreased in Elav-Gal4>UAS-Ssdp-RNAi and Ssdp[2082]/+ flies compared to their respective controls (1 h, n = 35–45) (middle). The schematic on the right shows the architecture of the feeding behavior of genotypic control and experimental flies (not drawn to scale). In scatter plots, each dot represents the mean value for a single fly. The red horizontal line represents the mean value. P-values are from the permutation t test. The raw data underlying this figure can be found in S1 Data file. (TIF) [file pbio.3002210.s008.tif]

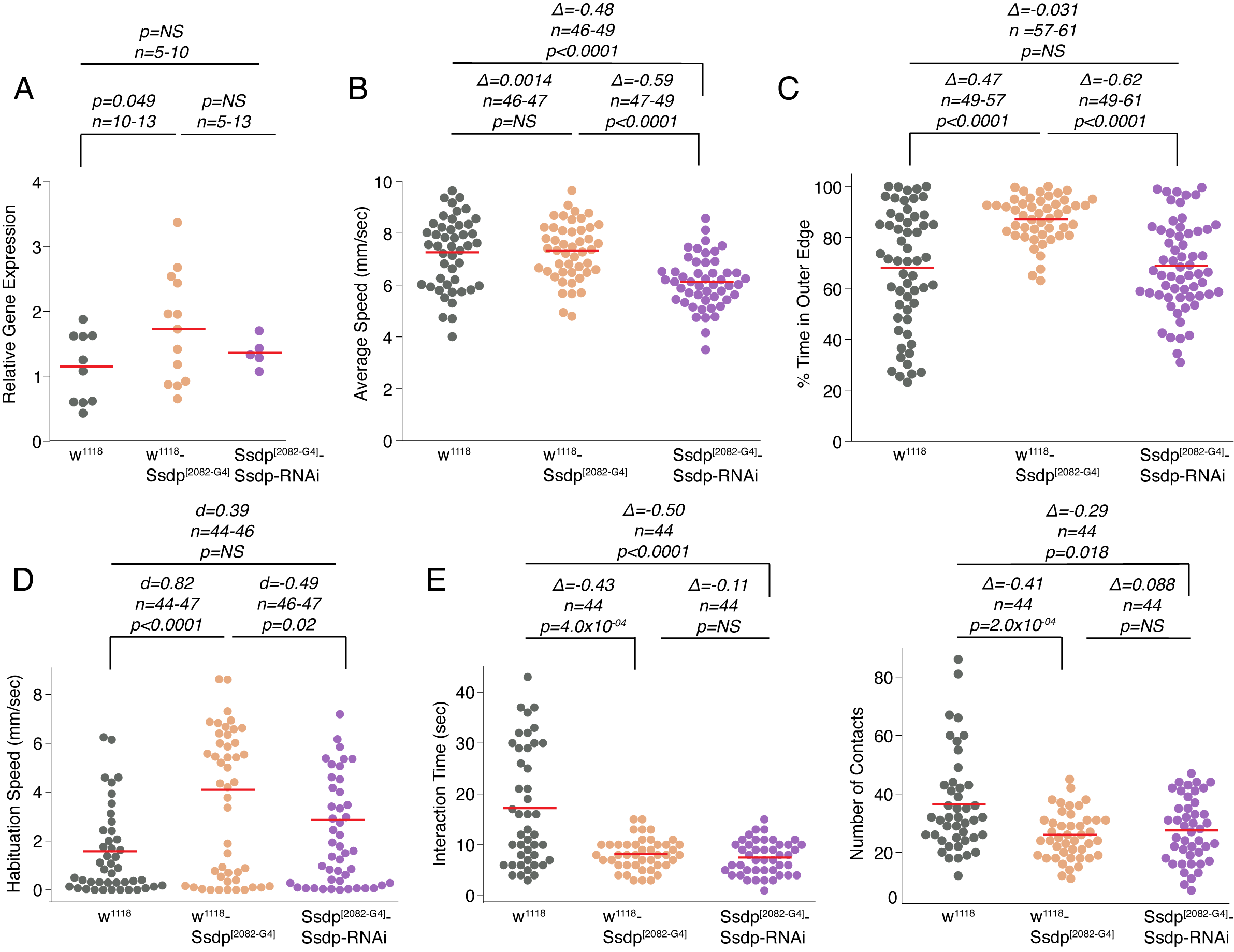

Supplement: S9 Fig — (A) Relative Ssdp mRNA expression in the heads of heterozygous Ssdp[2082]/+ flies was enhanced (p = 0.049, n = 10–13) compared to w1118 controls, but was not different in heads of Ssdp[2082-G4]>UAS-Ssdp-RNAi flies compared to w1118 controls (p = 0.26, n = 5–10) and Ssdp[2082]/+ flies (p = 0.16, n = 5–13). Each black dot is a data point representing an independent biological replicate with 20 individual fly heads per biological replicate. Total cDNA concentration was normalized to endogenous Rpl32 expression. P-values are calculated using the Student’s t test. (B) The active average speed of Ssdp[2082-G4] /+ flies was similar to control flies (n = 46–47, Cliff’s Δ = 0.0014 [95 CI −0.235, 0.24], p = 0.99); however, Ssdp[2082-G4] >UAS-Ssdp-RNAi flies moved slower in open field compared to w1118 controls (n = 46–49, Cliff’s Δ = −0.48 [95 CI −0.667, −0.248], p < 0.0001) and Ssdp[2082]/+ flies (n = 47–49, Cliff’s Δ = −0.59 [95 CI −0.755, −0.391], p < 0.0001). (C) Heterozygous Ssdp[2082-G4] /+ flies show a significantly higher wall-following compared to controls (n = 49–57, Cliff’s Δ = 0.47 [95 CI 0.256, 0.651], p < 0.0001); however, Ssdp[2082-G4]>UAS-Ssdp-RNAi flies show wall-following behavior similar to controls (n = 57–61, Cliff’s Δ = −0.031 [95 CI −0.246, 0.183], p = 0.78) and significantly decreased wall-following compared to Ssdp[2082]/+ flies (n = 49–61, Cliff’s Δ = −0.62 [95 CI −0.768, −0.428], p < 0.0001). (D) Ssdp[2082-G4] flies show defective habituation speed to the fifth pulse (n = 44–47, Cohen’s d = 0.82 [95 CI 0.391, 1.27], p < 0.0001), Ssdp[2082-G4]>UAS-Ssdp-RNAi flies show no habituation defect compared to w1118 controls (n = 44–46, Cohen’s d = 0.39 [95 CI −0.0448, 0.796], p = 0.064) and a significantly decreased habituation speed compared to Ssdp[2082]/+ flies (n = 46–47, Cohen’s d = −0.49 [95 CI −0.923, −0.0548], p = 0.02. (E) Both Ssdp[2082-G4]/+ (n = 44, Cliff’s Δ = −0.43 [95 CI −0.63, −0.185], p = 4.0 × 10^−04^) and Ssdp[2082-G4]>UAS-Ssdp-RNAi (n = 44, [file pbio.3002210.s009.tif]

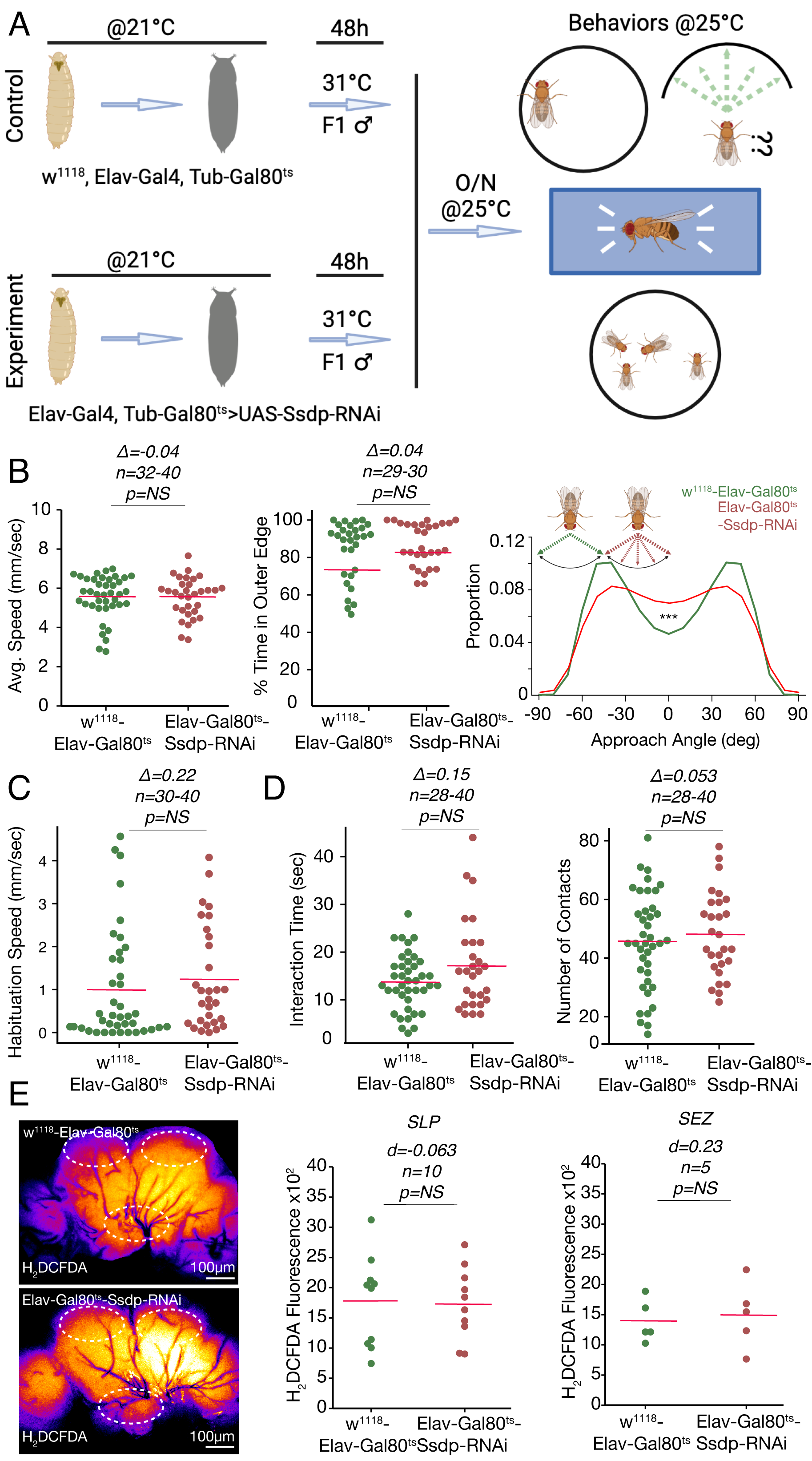

Supplement: S10 Fig — (A) Schematic depicting the timeline of experimental procedure on w1118>Elav-Gal4, Tub-Gal80ts and Elav-Gal4, Tub-Gal80ts>UAS-Ssdp-RNAi flies. Crosses were maintained at 21°C, after eclosion the adult F1s were conditioned at 31°C for 48 h, then kept at 25°C overnight, following which behavioral and functional assays were performed. Created with BioRender.com. (B) In the open field arena, no changes were observed in average speed (n = 32–40, Cliff’s Δ = −0.04 [95 CI −0.32, 0.23], p = 0.79) and percentage time spent in outer edge (n = 29–30, Cliff’s Δ = 0.04 [95 CI −0.27, 0.34], p = 0.78); however, wall approach angles were statistically significantly different between Elav-Gal4, Tub-Gal80ts>UAS-Ssdp-RNAi flies and w1118>Elav-Gal4, Tub-Gal80ts controls. Fly representations in wall approach angles graph created with BioRender.com. (C) Habituation speed to the fifth blue light pulse is unaltered in Elav-Gal4, Tub-Gal80ts>UAS-Ssdp-RNAi flies compared to w1118>Elav-Gal4, Tub-Gal80ts controls (n = 30–40, Cliff’s Δ = 0.22, [95 CI −0.0625, 0.47], p = 0.12). (D) In the social interaction assay, no differences were observed in the interaction time (n = 28–40, Cliff’s Δ = 0.15 [95 CI −0.15, 0.42], p = 0.31) and number of contacts (n = 28–40, Cliff’s Δ = 0.053 [95 CI −0.22, 0.32], p = 0.72) between Elav-Gal4, Tub-Gal80ts>UAS-Ssdp-RNAi flies and w1118>Elav-Gal4, Tub-Gal80ts controls. (E) Pseudocolor representative images of w1118>Elav-Gal4, Tub-Gal80ts and Elav-Gal4, Tub-Gal80ts>UAS-Ssdp-RNAi brains stained with H2DCFDA. White dashed ovals mark regions of interest in the SLP and SEZ. H2DCFDA fluorescence is not different in both SLP (n = 10, Cohen’s d = −0.063 [95 CI −1.03, 0.919], p = 0.89) and SEZ (n = 5, Cohen’s d = 0.23 [95 CI −1.25, 1.82], p = 0.72) regions between Elav-Gal4, Tub-Gal80ts>UAS-Ssdp-RNAi flies and w1118>Elav-Gal4, Tub-Gal80ts controls. In scatter plots, each dot represents the mean value for a single fly. Horizontal red line represents the mean value. P-values [file pbio.3002210.s010.tif]

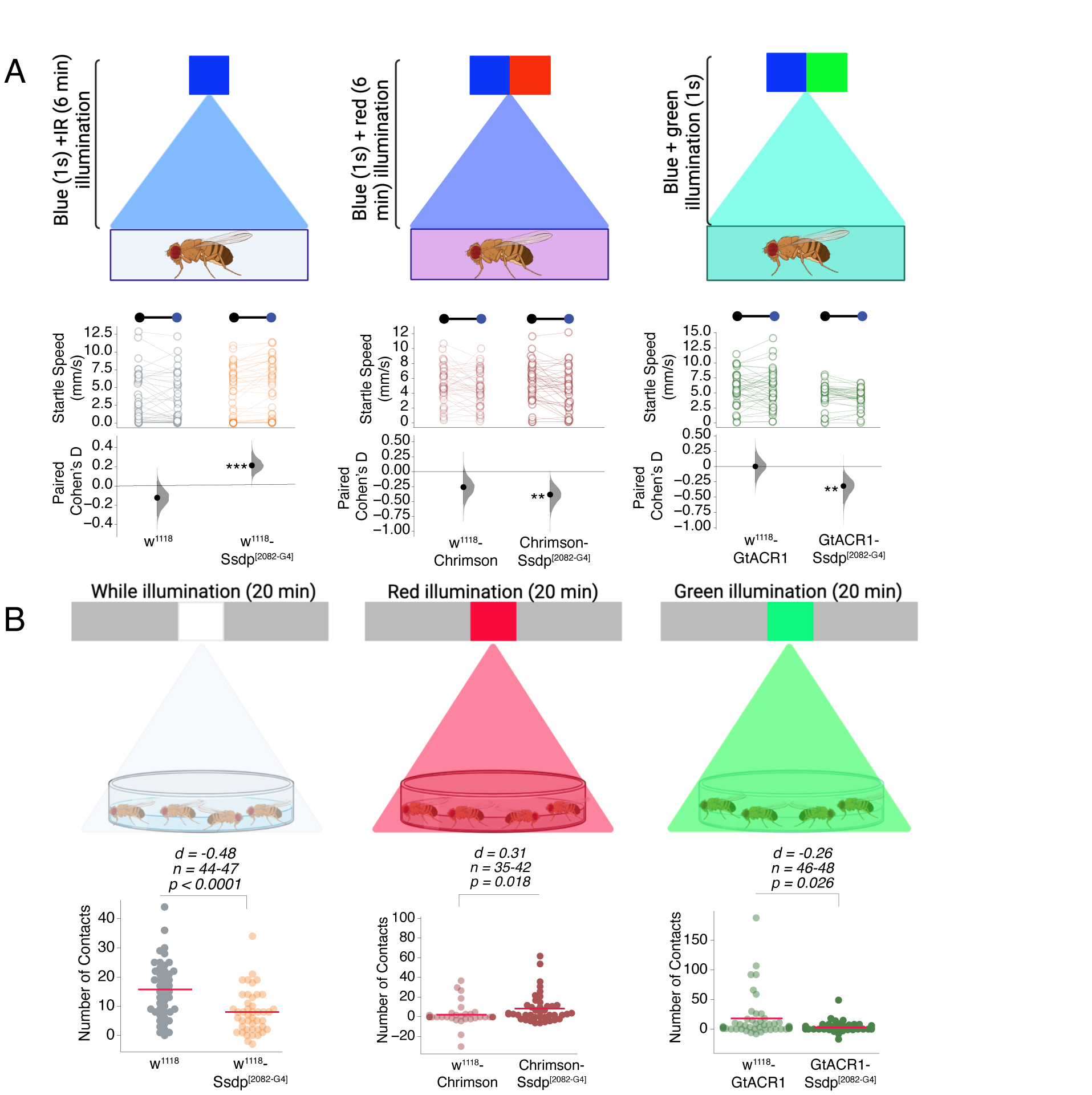

Supplement: S11 Fig — (A) Startle speed is significantly increased in heterozygous Ssdp[2082-G4]/+ flies (w1118, n = 43, Cohen’s d = −0.12 [95 CI −0.312, 0.0533], p = 0.183; Ssdp[2082-G4]/+, n = 43, Cohen’s d = 0.21 [95 CI 0.105, 0.359], p = 6 × 10^−04^); however, it is significantly decreased in Ssdp[2082-G4]>UAS-Chrimson (w1118>UAS-Chrimson, n = 43, Cohen’s d = −0.26 [95 CI −0.544, 0.0314], p = 0.083; Ssdp[2082-G4]>UAS-Chrimson, n = 43, Cohen’s d = −0.385 [95 CI −0.674, −0.146], p = 0.0044) and Ssdp[2082-G4]>UAS-GtACR1 (w1118>UAS-GtACR1, n = 38, Cohen’s d = 0.00153 [95 CI −0.258, 0.239], p = 0.99; Ssdp[2082-G4] >UAS-GtACR1, n = 34, Cohen’s d = −0.32 [95 CI −0.605, −0.0846], p = 0.0064) flies compared to their controls. Schematic created with BioRender.com. (B) Number of contacts is significantly decreased in Ssdp[2082-G4]/+ flies (n = 44–47, Cliff’s Δ = −0.48 [95 CI −0.662, −0.26], p < 0.0001) and Ssdp[2082-G4] >UAS-GtACR1 flies (n = 35–42, Cliff’s Δ = −0.26 [95 CI −0.478, −0.0285], p = 0.026); however, it is significantly increased in Ssdp[2082-G4]>UAS-Chrimson flies (n = 35–42, Cliff’s Δ = 0.31 [95 CI 0.0374, 0.546], p = 0.018) compared to their controls. Schematic created with BioRender.com. In scatter plots, each dot represents the mean value for a single fly. Horizontal red line represents the mean value. P-values are from a two-sided permutation t test. The raw data underlying this figure can be found in S1 Data file. (TIF) [file pbio.3002210.s011.tif]
