# Supplementary material for: The ortholog of human ssDNA-binding protein SSBP3 influences neurodevelopment and autism-like behaviors in Drosophila melanogaster
Source: PLoS Biol. 2023 Jul 24;21(7):e3002210. doi: 10.1371/journal.pbio.3002210 (PMC10399856; doi:10.1371/journal.pbio.3002210)
Supplement: S1 Table — Human genes that have orthologs in Drosophila melanogaster and their DIOPT scores are presented. The stock numbers for available transgenic lines in Bloomington Drosophila Stock Center (BDSC) or Vienna Drosophila Resource Center (VDRC) are mentioned. (DOCX) [file pbio.3002210.s012.docx]

| **Human Gene** | **Fly Gene** | **DIOPT Score** | **Mutant (BDSC#)** | **RNAi stock (BDSC#)** | **GAL4 (BDSC#)** | **UAS (BDSC#)** | **Human cDNA (BDSC#)** |
| --- | --- | --- | --- | --- | --- | --- | --- |
| *YIPF1* | *CG4645* | 1 | 33294 | v10164 (VDRC), v105497 (VDRC) |  | F003203 (FlyORF) |  |
| *SCP2* | *ScpX* | 0.933 | 27098 | 51479 |  |  |  |
| *CPT2* | *CPT2* | 0.933 | 18574, 59261 | 51900, 62455 |  |  |  |
| *CZIB* | *CG4646* | 0.933 | 63889, 81970, 13699 | 63646 |  |  |  |
| *MAGOH* | *mago* | 0.933 | 82653 | 28931, 35453, 55260 |  |  | 76853 |
| *SSBP3* | *Ssdp* | 0.933 | 65717, 20650, 85081, 91595, 13020 | 62167 | 65717 |  | 84852 |
| *TTC4* | *Dpit47* | 0.933 | 22239 | v34017 (VDRC), v110401 (VDRC) |  |  |  |
| *PARS2* | *ProRS-m* | 0.933 | 17811, 81808 | v330525 (VDRC), v330560 (VDRC) |  |  |  |
| *NDC1* | *Ndc1* | 0.866 | 31850 | 67275 |  |  |  |
| *CYB5RL* | *CG7914* | 0.866 | 33511 | v22618 (VDRC), v106416 (VDRC) |  |  |  |
| *MRPL37* | *mRpL37* | 0.8 | 22011 | 67906 |  |  |  |
| *ECHDC2* | *CG8778* | 0.73 | 10024, 76973 | 36793, 64508 |  |  |  |
| *LRP8* | *LpR2* | 0.73 | 17394, 38052, 43774, 44233, 60219, 85439, 93805 | 31150, 54461 |  |  |  |
| *FAM151A* | *CG7231* | 0.533 | 22332 | v2783 (VDRC), v107040 (VDRC) |  |  |  |
| *SLC1A7* | *Eaat1* | 0.466 | 22423, 32771, 86342 | 43287 | 8849 | 8202 |  |
| *DIO1* | *pps* | 0.466 | 68060 | 38529, 38912, 80410 |  |  |  |
| *GLIS1* | *lmd* | 0.4 | 91553 | 42871 | 77663 | PMID: 25568052 | 92093 |
| *USP24* | *faf* | 0.266 | 15390, 65538, 25100, 25102, 25107, 25108 | 35728 |  | 25102 |  |
| *TCEANC2* | *CG8117* | 0.13 |  | 77385 |  |  |  |
| *PCSK9* | *Fur2* | 0.13 | 11429 | 25959, 42577, 51743 |  | 63081 |  |
| *ZYG11B* | *CG12084* | 0.066 | 23756, 81946, 12996 | 34553 |  |  |  |
| *PODN* | *2mit* | 0.066 | 34219, 53196, 83041 | 28553 |  |  |  |
| *HSPB11* | *APC10* | 0.066 | 17921 | 34858 |  | F001490 (FlyORF) |  |
| *LDLRAD1* | *CG6553* | 0.066 |  | 61897, 67839 |  |  |  |
| *CDCP2* | *CG7179* | 0.066 |  | 77453 |  |  |  |
| *ACOT11* | *CG13771* | 0.066 |  | v31868 (VDRC), v31869 (VDRC) |  |  |  |
| *MROH7* | *c11.1* | 0.066 | 21420 | 61204 |  |  |  |
| *LEXM* | *CG14507* | 0.066 | 10310 | 33345 |  |  |  |
| *DHCR24* | *Agps* | 0.066 | 18510, 76519, 79234 | 34350 |  |  |  |
| *LRRC42* | no ortholog |  |  |  |  |  |  |
| *TMEM59* | no ortholog |  |  |  |  |  |  |
| *MIR4781* | no ortholog |  |  |  |  |  |  |
| *TTC22* | no ortholog |  |  |  |  |  |  |
| *TMEM61* | no ortholog |  |  |  |  |  |  |
| *BSND* | no ortholog |  |  |  |  |  |  |
