# Supplementary material for: The ortholog of human ssDNA-binding protein SSBP3 influences neurodevelopment and autism-like behaviors in Drosophila melanogaster
Source: PLoS Biol. 2023 Jul 24;21(7):e3002210. doi: 10.1371/journal.pbio.3002210 (PMC10399856; doi:10.1371/journal.pbio.3002210)
Supplement: S2 Table — A fold change of 1.5 was set as the criteria, based on the fold change of Ssdp gene. According to this criteria, 256 genes were down-regulated and 160 genes were up-regulated. The fold change, p-value, human ortholog, and DIOPT score are mentioned for each dysregulated gene. (DOCX) [file pbio.3002210.s013.docx]

| **UPREGULATED GENES** | | | | |  | **DOWNREGULATED GENES** | | | | |
| --- | --- | --- | --- | --- | --- | --- | --- | --- | --- | --- |
| **Gene_Symbol** | **SSDP/CTL.** | | **Human ortholog** | **Orthology Score** |  | **Gene_Symbol** | **SSDP/CTL.** | | **Human ortholog** | **Orthology Score** |
|  | **Fold Change** | **bh. p-value** |  |  |  |  | **Fold Change** | **bh. p-value** |  |  |
| *lncRNA:CR31386* | 85.18 | 0.0000 | *-* | - |  | *CG40486* | -1.50 | 0.0000 | *DHRS11* | 12 |
| *CG33459* | 79.15 | 0.0000 | *PRSS45P* | 2 |  | *CG2121* | -1.51 | 0.0011 | *UNC93A* | 11 |
| *l(2)03659* | 40.82 | 0.0000 | *ABCC4* | 11 |  | *CG10672* | -1.51 | 0.0000 | *DHRS4* | 15 |
| *w* | 38.93 | 0.0000 | *ABCG2* | 8 |  | *CG33543* | -1.51 | 0.0000 | *IGLON5* | 3 |
| *lncRNA:CR44301* | 27.30 | 0.0000 | *-* | - |  | *CG10345* | -1.51 | 0.0002 | *SCARB1* | 5 |
| *Or42a* | 21.51 | 0.0000 | *NA* | - |  | *CG30203* | -1.52 | 0.0002 | *SPON1* | 7 |
| *CG42364* | 9.33 | 0.0000 | *NA* | - |  | *Qsox2* | -1.52 | 0.0119 | *QSOCX2* | 15 |
| *ranshi* | 7.48 | 0.0000 | *ZNF281* | 2 |  | *CG13510* | -1.52 | 0.0229 | *LITAF* | 6 |
| *CG10660* | 7.09 | 0.0000 | *NA* | - |  | *CG15547* | -1.52 | 0.0000 | *NME5* | 5 |
| *CG30087* | 7.04 | 0.0000 | *NA* | - |  | *CG11498* | -1.53 | 0.0319 | *SPATA18* | 1 |
| *lncRNA:CR9162* | 6.83 | 0.0183 | *-* | - |  | *Ada2a* | -1.54 | 0.0000 | *TADA2A* | 12 |
| *CG31220* | 6.58 | 0.0000 | *CMA1* | 1 |  | *CG42390* | -1.54 | 0.0000 | *RANBP3* | 1 |
| *snRNA:U2:34ABa* | 6.36 | 0.0000 | *NA* | - |  | *CG13116* | -1.54 | 0.0000 | *NA* | NA |
| *GILT3* | 6.29 | 0.0000 | *IFI30* | 7 |  | *CG14400* | -1.54 | 0.0007 | *NA* | NA |
| *CG30088* | 5.78 | 0.0000 | *PRSS45P* | 1 |  | *dnd* | -1.54 | 0.0257 | *ARL3* | 12 |
| *inaF-A* | 5.08 | 0.0001 | *NA* |  |  | *CG3348* | -1.54 | 0.0080 | *NA* | NA |
| *stet* | 4.99 | 0.0000 | *RHBDL3* | 14 |  | *CG18302* | -1.54 | 0.0020 | *LIPA* | 10 |
| *CG6142* | 4.72 | 0.0000 | *CHDH* | 6 |  | *Pu* | -1.55 | 0.0001 | *GCH1* | 14 |
| *lncRNA:CR44300* | 4.68 | 0.0001 | *-* | - |  | *dob* | -1.55 | 0.0004 | *PNPLA2* | 10 |
| *can* | 4.52 | 0.0335 | *TAF5* | 7 |  | *CG42807* | -1.56 | 0.0251 | *NA* | NA |
| *CG3397* | 4.46 | 0.0000 | *AKR7A2* | 3 |  | *CG31075* | -1.57 | 0.0003 | *AlDH1A1* | 7 |
| *CG4650* | 4.32 | 0.0000 | *NA* | - |  | *SmydA-3* | -1.57 | 0.0146 | *SMYD1* | 3 |
| *Edg78E* | 4.25 | 0.0000 | *NA* | - |  | *AANAT1* | -1.57 | 0.0017 | *NA* | NA |
| *lncRNA:CR43978* | 4.23 | 0.0034 | *-* | - |  | *Cyp317a1* | -1.58 | 0.0000 | *CYP3A4* | 4 |
| *lncRNA:CR45997* | 4.17 | 0.0000 | *-* | - |  | *GstE11* | -1.60 | 0.0029 | *GSTT2B* | 3 |
| *lncRNA:CR45337* | 4.13 | 0.0101 | *-* | - |  | *CG7470* | -1.60 | 0.0000 | *ALDH18A1* | 15 |
| *CG9451* | 4.13 | 0.0000 | *ACP2* | 6 |  | *cDIP* | -1.61 | 0.0016 | *CPN2* | 1 |
| *CR43671* | 3.87 | 0.0002 | *NA* | - |  | *asRNA:CR45600* | -1.62 | 0.0004 | *NA* | NA |
| *CG9377* | 3.71 | 0.0000 | *F9* | 1 |  | *RhoGAP54D* | -1.63 | 0.0485 | *ARHGAP19* | 12 |
| *Cyp6w1* | 3.70 | 0.0000 | *CYP3A4* | 7 |  | *CG4335* | -1.64 | 0.0000 | *TMLHE* | 14 |
| *CG30287* | 3.68 | 0.0000 | *PRSS45P* | 2 |  | *CG17129* | -1.65 | 0.0321 | *NA* | NA |
| *Spn42Db* | 3.66 | 0.0126 | *SERPINI1* | 6 |  | *CG10208* | -1.66 | 0.0003 | *NA* | NA |
| *CG12224* | 3.63 | 0.0000 | *AKR7A2* | 3 |  | *CG13618* | -1.66 | 0.0000 | *NA* | NA |
| *Ir76a* | 3.61 | 0.0000 | *GRID2* | 2 |  | *Obp57a* | -1.66 | 0.0000 | *NA* | NA |
| *Cpr50Ca* | 3.60 | 0.0308 | *NA* | - |  | *CG11619* | -1.66 | 0.0000 | *GPCPD1* | 8 |
| *CR15280* | 3.50 | 0.0000 | *NA* | - |  | *CG13325* | -1.67 | 0.0000 | *LOC112268211* | 2 |
| *lncRNA:CR45681* | 3.46 | 0.0000 | *-* | - |  | *CG10361* | -1.69 | 0.0376 | *GCAT* | 15 |
| *ninaD* | 3.45 | 0.0000 | *SCARB1* | 6 |  | *CR44391* | -1.69 | 0.0335 | *NA* | NA |
| *CNT2* | 3.38 | 0.0091 | *SLC28A2* | 14 |  | *CG7900* | -1.70 | 0.0003 | *FAAH2* | 6 |
| *CG30090* | 3.34 | 0.0000 | *NA* |  |  | *CG31278* | -1.70 | 0.0311 | *PDF* | 11 |
| *CG18557* | 3.29 | 0.0003 | *TPSAB2* | 2 |  | *CG13117* | -1.70 | 0.0000 | *NA* | NA |
| *p24-2* | 3.27 | 0.0000 | *TMED4* | 12 |  | *CG30050* | -1.70 | 0.0210 | *NA* | NA |
| *lncRNA:CR44602* | 3.19 | 0.0011 | *-* | - |  | *CG1113* | -1.71 | 0.0140 | *FAM161A* | 2 |
| *lncRNA:CR44120* | 3.07 | 0.0026 | *-* | - |  | *CG13796* | -1.71 | 0.0000 | *SLC6A4* | 4 |
| *CG17855* | 3.00 | 0.0023 | *NA* |  |  | *Nepl20* | -1.72 | 0.0020 | *KEL* | 6 |
| *CG42260* | 2.97 | 0.0000 | *CNGA2* | 4 |  | *Vajk1* | -1.72 | 0.0001 | *NA* | NA |
| *Kebab* | 2.78 | 0.0235 | *NA* |  |  | *CG3609* | -1.73 | 0.0001 | *DHDH* | 13 |
| *CG3117* | 2.71 | 0.0000 | *TPSAB1* | 2 |  | *primo-1* | -1.73 | 0.0000 | *ACP1* | 12 |
| *CG42876* | 2.71 | 0.0000 | *NA* | - |  | *lncRNA:CR43417* | -1.73 | 0.0164 | *NA* | NA |
| *lncRNA:CR13130* | 2.63 | 0.0000 | *-* | - |  | *CheB42c* | -1.73 | 0.0252 | *NA* | NA |
| *lncRNA:CR40469* | 2.57 | 0.0000 | *-* | - |  | *CG42740* | -1.74 | 0.0000 | *NA* | NA |
| *Def* | 2.52 | 0.0288 | *NA* | - |  | *FANCI* | -1.75 | 0.0333 | *FANCI* | 13 |
| *Boot* | 2.49 | 0.0000 | *NA* | - |  | *Amy-d* | -1.75 | 0.0000 | *AMY2B* | 12 |
| *Lime* | 2.47 | 0.0162 | *PLAGL2* | 1 |  | *qin* | -1.76 | 0.0002 | *TDRD1* | 6 |
| *TotB* | 2.38 | 0.0047 | *NA* | - |  | *Obp99b* | -1.76 | 0.0016 | *NA* | NA |
| *TotM* | 2.35 | 0.0002 | *NA* | - |  | *CG34227* | -1.76 | 0.0254 | *NA* | NA |
| *mthl3* | 2.31 | 0.0038 | *ADGRG7* | 2 |  | *asRNA:CR43259* | -1.76 | 0.0001 | *NA* | NA |
| *lncRNA:CR43883* | 2.31 | 0.0073 | *-* | - |  | *CG16965* | -1.76 | 0.0000 | *PGGHG* | 14 |
| *phr* | 2.30 | 0.0000 | *NA* | - |  | *Cyp28a5* | -1.77 | 0.0000 | *CYP3A4* | 4 |
| *trnL2* | 2.27 | 0.0000 | *-* | - |  | *GstE1* | -1.78 | 0.0000 | *GSTT2B* | 4 |
|  | 2.24 | 0.0000 | *CYP3A4* | 8 |  | *Spn88Eb* | -1.78 | 0.0000 | *SERPINB1* | 4 |
| *Gdap1* | 2.18 | 0.0011 | *GDAP1* | 13 |  | *CG11626* | -1.78 | 0.0372 | *PRSS16* | 4 |
| *jtb* | 2.16 | 0.0000 | *NA* | - |  | *Cyp9b1* | -1.79 | 0.0002 | *CYP3A5* | 8 |
| *CG43109* | 2.16 | 0.0452 | *NA* | - |  | *ppk29* | -1.80 | 0.0019 | *ASIC1* | 5 |
| *BomT1* | 2.15 | 0.0010 | *NA* | - |  | *CG8620* | -1.81 | 0.0225 | *NA* | NA |
| *BomBc3* | 2.12 | 0.0000 | *NA* | - |  | *CG44008* | -1.81 | 0.0000 | *SPINK7* | 3 |
| *CG15143* | 2.12 | 0.0009 | *MAATS1* | 8 |  | *mthl8* | -1.81 | 0.0000 | *ADGRF3* | 1 |
| *Tsp29Fa* | 2.12 | 0.0386 | *CD63* | 8 |  | *Cralbp* | -1.82 | 0.0025 | *CLVS1* | 5 |
| *CG43179* | 2.11 | 0.0494 | *NA* | - |  | *CG33293* | -1.82 | 0.0318 | *NA* | NA |
| *Pepck2* | 2.08 | 0.0179 | *PCK2* | 14 |  | *Ir93a* | -1.83 | 0.0358 | *GRIA1* | 3 |
| *Cyp6a14* | 2.04 | 0.0041 | *CYP3A4* | 5 |  | *lncRNA:CR32658* | -1.84 | 0.0267 | *NA* | NA |
| *phr6-4* | 2.02 | 0.0229 | *CRY2* | 8 |  | *Cpr49Ag* | -1.85 | 0.0223 | *NA* | NA |
| *lncRNA:CR46123* | 2.01 | 0.0063 | *-* | - |  | *CG9928* | -1.86 | 0.0000 | *NA* | NA |
| *Cyp4e3* | 1.99 | 0.0000 | *CYP4V2* | 6 |  | *Orc1* | -1.86 | 0.0007 | *ORC1* | 12 |
| *Ugt35C1* | 1.99 | 0.0000 | *UGT2B15* | 8 |  | *cup* | -1.87 | 0.0089 | *EIF4ENIF1* | 3 |
| *CG13658* | 1.96 | 0.0267 | *NA* | - |  | *CG42369* | -1.87 | 0.0000 | *NA* | NA |
| *CG34305* | 1.95 | 0.0146 | *NA* | - |  | *CG11825* | -1.87 | 0.0231 | *HIGD1A* | 12 |
| *CG3270* | 1.94 | 0.0000 | *FOXRED1* | 13 |  | *asRNA:CR45835* | -1.87 | 0.0001 | *NA* | NA |
| *ND2* | 1.93 | 0.0240 | *ND2* | 12 |  | *Cpr62Bb* | -1.89 | 0.0000 | *NA* | NA |
| *CG18547* | 1.93 | 0.0005 | *AKR7A2* | 3 |  | *Rpp21* | -1.89 | 0.0000 | *RPP21* | 9 |
| *CG7054* | 1.92 | 0.0000 | *PEBP1* | 6 |  | *CG17786* | -1.90 | 0.0178 | *NA* | NA |
| *lncRNA:Hsromega* | 1.90 | 0.0000 | *-* | - |  | *Amy-p* | -1.91 | 0.0000 | *AMY2B* | 13 |
| *CG34354* | 1.90 | 0.0000 | *TIA1* | 7 |  | *CG31664* | -1.93 | 0.0000 | *NA* | NA |
| *CG11842* | 1.90 | 0.0000 | *GZMA* | 1 |  | *CG40160* | -1.93 | 0.0000 | *TPSAB1* | 3 |
| *CG30148* | 1.89 | 0.0036 | *NA* | - |  | *CG10560* | -1.93 | 0.0000 | *NA* | NA |
| *CG34423* | 1.88 | 0.0000 | *ATP5IF1* | 6 |  | *CG15279* | -1.93 | 0.0000 | *SLC6A7* | 4 |
| *CG15362* | 1.88 | 0.0346 | *HINT3* | 11 |  | *asRNA:CR44065* | -1.94 | 0.0296 | *NA* | NA |
| *CG5245* | 1.87 | 0.0098 | *ZNF45* | 3 |  | *pre-mod(mdg4)-H* | -1.96 | 0.0091 | *FLYWCH1* | 1 |
| *CG40470* | 1.86 | 0.0000 | *ANPEP* | 3 |  | *CG9192* | -1.96 | 0.0422 | *NA* | NA |
| *CG5639* | 1.86 | 0.0000 | *PI3* | 3 |  | *CG8757* | -1.96 | 0.0000 | *DHRS11* | 11 |
| *janA* | 1.86 | 0.0000 | *PHPT1* | 13 |  | *lncRNA:CR44645* | -1.97 | 0.0009 | *NA* | NA |
| *ND3* | 1.85 | 0.0000 | *ND3* | 11 |  | *CG34256* | -1.97 | 0.0001 | *NA* | NA |
| *CG15213* | 1.85 | 0.0100 | *NA* | - |  | *CG3982* | -1.98 | 0.0001 | *ATAT1* | 1 |
| *CG6026* | 1.85 | 0.0245 | *CNGA1* | 1 |  | *CG15539* | -1.98 | 0.0003 | *P4HA3* | 5 |
| *asRNA:CR44030* | 1.83 | 0.0000 | *NA* | - |  | *pr* | -2.04 | 0.0000 | *PTS* | 14 |
| *CG11741* | 1.83 | 0.0108 | *NA* | - |  | *CG12229* | -2.05 | 0.0462 | [*PKLR*](https://www.genenames.org/data/gene-symbol-report/#!/hgnc_id/9020) | 4 |
| *Cyp6d2* | 1.83 | 0.0280 | *CYP3A4* | 4 |  | *asRNA:CR45822* | -2.06 | 0.0000 | *NA* | NA |
| *BomS5* | 1.82 | 0.0000 | *NA* | - |  | *PGRP-SD* | -2.08 | 0.0000 | *PGLYRP1* | 7 |
| *CG16898* | 1.82 | 0.0258 | *NA* | - |  | *lncRNA:CR46483* | -2.08 | 0.0000 | *NA* | NA |
| *Skeletor* | 1.81 | 0.0001 | *THBD* | 1 |  | *Dhc98D* | -2.09 | 0.0000 | *DNAH10* | 14 |
| *PPO2* | 1.80 | 0.0000 | *NA* |  |  | *ZnT33D* | -2.09 | 0.0041 | *SLC30A2* | 10 |
| *Unc-115b* | 1.80 | 0.0000 | *ABLIM1* | 8 |  | *se* | -2.20 | 0.0000 | *GSTO1* | 14 |
| *retinin* | 1.77 | 0.0210 | *TMEM38A* | 1 |  | *CG8908* | -2.21 | 0.0003 | *ABCA3* | 7 |
| *CG4259* | 1.77 | 0.0000 | *TPSAB1* | 1 |  | *betaTub85D* | -2.24 | 0.0001 | *TUBB2B* | 9 |
| *CG14464* | 1.77 | 0.0000 | *ARL14EP* | 8 |  | *CG5435* | -2.24 | 0.0143 | *C1orf194* | 10 |
| *CG11459* | 1.76 | 0.0099 | *CTSL* | 4 |  | *CG30339* | -2.25 | 0.0232 | *CLVS1* | 3 |
| *CG14630* | 1.76 | 0.0362 | *BBOX1* | 12 |  | *CG13168* | -2.27 | 0.0000 | *IQCD* | 6 |
| *hng3* | 1.75 | 0.0019 | *NA* |  |  | *asRNA:CR44370* | -2.30 | 0.0027 | *NA* | NA |
| *Hsp23* | 1.75 | 0.0000 | *CRYAB* | 7 |  | *Osi2* | -2.32 | 0.0000 | *NA* | NA |
| *lncRNA:CR44525* | 1.75 | 0.0082 | *-* | - |  | *CG8526* | -2.32 | 0.0259 | *ASPG* | 13 |
| *lncRNA:CR45054* | 1.74 | 0.0481 | *-* | - |  | *Dnah3* | -2.34 | 0.0165 | *DNAH3* | 13 |
| *Gyc32E* | 1.74 | 0.0049 | *GUCY2F* | 5 |  | *Porin2* | -2.34 | 0.0362 | *VDAC3* | 8 |
| *CG7829* | 1.73 | 0.0000 | *PRSS1* | 2 |  | *CG30091* | -2.39 | 0.0274 | *PRSS53* | 1 |
| *CG31370* | 1.72 | 0.0000 | *NA* | - |  | *CG5653* | -2.40 | 0.0000 | *PAOX* | 6 |
| *Ctl2* | 1.71 | 0.0000 | *SLC44A2* | 13 |  | *Ssl2* | -2.40 | 0.0000 | *APMAP* | 13 |
| *asRNA:CR43426* | 1.70 | 0.0005 | *NA* | - |  | *CG17490* | -2.41 | 0.0157 | *RPL5* | 1 |
| *CG30083* | 1.70 | 0.0490 | *PRSS45P* | 1 |  | *IM18* | -2.42 | 0.0266 | *NA* | NA |
| *Ilp5* | 1.69 | 0.0029 | *INS* | 2 |  | *S-Lap1* | -2.43 | 0.0000 | *LAP3* | 11 |
| *BomS6* | 1.69 | 0.0000 | *NA* | - |  | *Fuca* | -2.45 | 0.0000 | *FUCA2* | 12 |
| *Kif3C* | 1.69 | 0.0000 | *KIF17* | 12 |  | *CG11322* | -2.47 | 0.0481 | *GCNA* | 4 |
| *CG8245* | 1.67 | 0.0000 | *TMEM53* | 14 |  | *CG4714* | -2.48 | 0.0168 | *CCDC96* | 3 |
| *thw* | 1.67 | 0.0422 | *NA* |  |  | *CG13022* | -2.51 | 0.0000 | *NA* | NA |
| *CG31955* | 1.66 | 0.0017 | *CHTF18* | 1 |  | *CG33189* | -2.54 | 0.0207 | *NA* | NA |
| *CG1791* | 1.65 | 0.0025 | *FCN1* | 3 |  | *hmw* | -2.55 | 0.0275 | *CFAP97* | 4 |
| *CG13305* | 1.65 | 0.0083 | *NA* |  |  | *LysX* | -2.59 | 0.0003 | *Lyz* | 10 |
| *Cyp6a20* | 1.64 | 0.0000 | *CYP3A4* | 5 |  | *CG12911* | -2.61 | 0.0255 | *NA* | NA |
| *CG9733* | 1.64 | 0.0142 | *F10* | 1 |  | *CG45045* | -2.63 | 0.0008 | *NA* | NA |
| *CG31199* | 1.63 | 0.0000 | *PAMR1* | 1 |  | *c-cup* | -2.72 | 0.0000 | *NENF* | 4 |
| *Ilp3* | 1.63 | 0.0000 | *IGF1* | 1 |  | *asRNA:CR44291* | -2.73 | 0.0108 | *NA* | NA |
| *CG42331* | 1.63 | 0.0203 | *EPX* | 2 |  | *Cyp6a2* | -2.75 | 0.0000 | *CYP3A4* | 6 |
| *CG11668* | 1.62 | 0.0001 | *THEM6* | 1 |  | *Cyp6a16* | -2.78 | 0.0000 | *CYP3A4* | 3 |
| *CG42494* | 1.61 | 0.0068 | *NA* |  |  | *CG10869* | -2.81 | 0.0000 | *QRICH2* | 3 |
| *Spc105R* | 1.61 | 0.0430 | *NA* |  |  | *CG9314* | -2.82 | 0.0234 | *CAT* | 13 |
| *t* | 1.61 | 0.0000 | *NA* |  |  | *lncRNA:alphagamma-element:CR32865* | -2.84 | 0.0001 | *NA* | NA |
| *THEM6* | 1.61 | 0.0111 | *NR4A2* | 13 |  | *lncRNA:CR42767* | -2.94 | 0.0392 | *NA* | NA |
| *PGRP-LD* | 1.60 | 0.0023 | *PGLYRP1* | 5 |  | *ste24b* | -2.95 | 0.0017 | *ZMPSTE24* | 7 |
| *GNBP1* | 1.60 | 0.0000 | *NA* |  |  | *CG2955* | -2.98 | 0.0086 | *MAPRE3* | 5 |
| *asRNA:CR46354* | 1.59 | 0.0422 | *NA* |  |  | *fan* | -3.00 | 0.0422 | *VAPB* | 8 |
| *CG8329* | 1.59 | 0.0000 | *CTRL* | 1 |  | *CG31178* | -3.01 | 0.0313 | *NA* | NA |
| *CG6125* | 1.59 | 0.0000 | *SLC26A11* | 6 |  | *lncRNA:CR46216* | -3.10 | 0.0237 | *NA* | NA |
| *Cyp309a2* | 1.59 | 0.0257 | *CYP3A4* | 3 |  | *CG11353* | -3.13 | 0.0004 | *LOC112268211* | 2 |
| *sr* | 1.57 | 0.0196 | *EGR2* | 6 |  | *CG32192* | -3.13 | 0.0026 | *NA* | NA |
| *IM14* | 1.57 | 0.0006 | *NA* | - |  | *CG11327* | -3.14 | 0.0107 | *NA* | NA |
| *CG3831* | 1.56 | 0.0143 | *NA* | - |  | *CG12470* | -3.17 | 0.0088 | *FOS* | 1 |
| *CG31676* | 1.56 | 0.0205 | *NA* | - |  | *CG4408* | -3.18 | 0.0000 | *CPA1* | 6 |
| *Unc-115a* | 1.56 | 0.0000 | *ABLIM1* | 13 |  | *alphaTub85E* | -3.21 | 0.0100 | *TUBA1A* | 8 |
| *lrRNA* | 1.55 | 0.0001 | *-* | - |  | *CG13046* | -3.25 | 0.0000 | *NA* | NA |
| *CG7135* | 1.55 | 0.0000 | *NA* | - |  | *CG7208* | -3.26 | 0.0171 | *NA* | NA |
| *Cpn* | 1.55 | 0.0000 | *COCH* | 1 |  | *krimp* | -3.26 | 0.0003 | *TDRD1* | 2 |
| *Ipk1* | 1.54 | 0.0055 | *IPPK* | 11 |  | *CG9920* | -3.36 | 0.0000 | *HSPE1* | 12 |
| *CG46309* | 1.54 | 0.0000 | *NA* |  |  | *CG4546* | -3.41 | 0.0047 | *CKMT2* | 8 |
| *CG7255* | 1.53 | 0.0002 | *SLC7A2* | 9 |  | *CG17005* | -3.44 | 0.0001 | *NA* | NA |
| *asRNA:CR45209* | 1.53 | 0.0154 | *NA* |  |  | *lncRNA:CR45461* | -3.45 | 0.0055 | *NA* | - |
| *Ada1-2* | 1.52 | 0.0004 | *TADA1* | 13 |  | *CG10841* | -3.45 | 0.0081 | *NA* | NA |
| *CG4666* | 1.52 | 0.0044 | *THEM6* | 13 |  | *CG4998* | -3.50 | 0.0005 | *PLG* | 1 |
| *Ssdp* | 1.51 | 0.0000 | *SSBP3* | 14 |  | *lncRNA:CR46350* | -3.57 | 0.0000 | *NA* | - |
| *lncRNA:noe* | 1.51 | 0.0000 | *-* | - |  | *Ir75b* | -3.61 | 0.0002 | *GRID1* | 3 |
| *CG34242* | 1.51 | 0.0083 | *SMIM20* | 4 |  | *CG43149* | -3.61 | 0.0055 | *NA* | - |
| *CG6000* | 1.50 | 0.0000 | *TSTD1* | 10 |  | *CG32436* | -3.64 | 0.0000 | *NA* | - |
| *Hexo1* | 1.50 | 0.0000 | *HEXB* | 9 |  | *CG17376* | -3.71 | 0.0018 | *NA* | - |
|  |  |  |  |  |  | *lncRNA:CR45457* | -3.77 | 0.0000 | *NA* | - |
|  |  |  |  |  |  | *salto* | -3.78 | 0.0000 | *KCTD9* | 1 |
|  |  |  |  |  |  | *CG4375* | -3.82 | 0.0168 | *NA* | NA |
|  |  |  |  |  |  | *ymp* | -3.87 | 0.0338 | *NA* | NA |
|  |  |  |  |  |  | *scpr-C* | -3.87 | 0.0001 | *GLIPR1L1* | 5 |
|  |  |  |  |  |  | *fit* | -3.89 | 0.0000 | *NA* | NA |
|  |  |  |  |  |  | *CG5280* | -3.93 | 0.0184 | *C22orf23* | 12 |
|  |  |  |  |  |  | *CG3494* | -3.96 | 0.0171 | *LRRC40* | 3 |
|  |  |  |  |  |  | *CG12995* | -3.96 | 0.0235 | *NA* | NA |
|  |  |  |  |  |  | *lncRNA:CR45456* | -3.98 | 0.0000 | *NA* | - |
|  |  |  |  |  |  | *Gpo3* | -4.03 | 0.0174 | *GPD2* | 4 |
|  |  |  |  |  |  | *polo* | -4.05 | 0.0185 | [*PLK1*](https://www.genenames.org/data/gene-symbol-report/#!/hgnc_id/9077) | 15 |
|  |  |  |  |  |  | *Mst84Da* | -4.05 | 0.0050 | *NA* | - |
|  |  |  |  |  |  | *CG30376* | -4.06 | 0.0180 | *NA* | - |
|  |  |  |  |  |  | *vrs* | -4.07 | 0.0000 | *NA* | - |
|  |  |  |  |  |  | *asRNA:CR45046* | -4.07 | 0.0000 | *NA* | - |
|  |  |  |  |  |  | *Mst87F* | -4.12 | 0.0239 | *NA* | - |
|  |  |  |  |  |  | *CG42855* | -4.19 | 0.0363 | *NA* | - |
|  |  |  |  |  |  | *lncRNA:CR32690* | -4.24 | 0.0003 | *NA* | - |
|  |  |  |  |  |  | *sip2* | -4.28 | 0.0024 | *NA* | - |
|  |  |  |  |  |  | *GstD5* | -4.32 | 0.0003 | *GSTT2B* | 3 |
|  |  |  |  |  |  | *gskt* | -4.40 | 0.0144 | [*GSK3B*](https://www.genenames.org/data/gene-symbol-report/#!/hgnc_id/4617) | 10 |
|  |  |  |  |  |  | *CG15109* | -4.42 | 0.0000 | *NA* | NA |
|  |  |  |  |  |  | *CG1394* | -4.46 | 0.0191 | *NA* | NA |
|  |  |  |  |  |  | *CG14191* | -4.47 | 0.0019 | *KRTAP5-5* | 1 |
|  |  |  |  |  |  | *CG7886* | -4.50 | 0.0000 | [*CEP78*](https://www.genenames.org/data/gene-symbol-report/#!/hgnc_id/25740) | 9 |
|  |  |  |  |  |  | *Ir75a* | -4.51 | 0.0000 | *NA* | NA |
|  |  |  |  |  |  | *AttA* | -4.51 | 0.0006 | *NA* | NA |
|  |  |  |  |  |  | *CG17470* | -4.55 | 0.0010 | *NA* | NA |
|  |  |  |  |  |  | *lncRNA:CR44851* | -4.62 | 0.0058 | *NA* | - |
|  |  |  |  |  |  | *soti* | -4.63 | 0.0002 | *NA* | NA |
|  |  |  |  |  |  | *saturn* | -4.65 | 0.0148 | *NA* | NA |
|  |  |  |  |  |  | *fzo* | -4.76 | 0.0008 | [*MFN2*](https://www.genenames.org/data/gene-symbol-report/#!/hgnc_id/16877) | 7 |
|  |  |  |  |  |  | *CG4631* | -4.77 | 0.0053 | *NA* | NA |
|  |  |  |  |  |  | *CG5043* | -4.84 | 0.0117 | *NA* | NA |
|  |  |  |  |  |  | *Obp56e* | -4.86 | 0.0000 | *NA* | NA |
|  |  |  |  |  |  | *CG31798* | -4.86 | 0.0185 | *NA* | NA |
|  |  |  |  |  |  | *exu* | -4.91 | 0.0000 | [*TREX1*](https://www.genenames.org/data/gene-symbol-report/#!/hgnc_id/12269) | 1 |
|  |  |  |  |  |  | *Pgm2b* | -4.92 | 0.0000 | *PGM2* | 11 |
|  |  |  |  |  |  | *CG13898* | -4.94 | 0.0148 | *CABP5* | 3 |
|  |  |  |  |  |  | *fest* | -5.04 | 0.0000 | *NA* | NA |
|  |  |  |  |  |  | *Nazo* | -5.18 | 0.0000 | *C19orf12* | 9 |
|  |  |  |  |  |  | *CG10252* | -5.18 | 0.0000 | *ODF3L2* | 11 |
|  |  |  |  |  |  | *CG18063* | -5.55 | 0.0152 | *SPATA17* | 6 |
|  |  |  |  |  |  | *CG5089* | -5.56 | 0.0001 | *NA* | NA |
|  |  |  |  |  |  | *CG31226* | -5.64 | 0.0000 | *NA* | NA |
|  |  |  |  |  |  | *CG1324* | -5.75 | 0.0005 | *NA* | NA |
|  |  |  |  |  |  | *CG8564* | -5.77 | 0.0011 | *CPA1* | 2 |
|  |  |  |  |  |  | *CG3492* | -5.89 | 0.0029 | [*PPIB*](https://pubmed.ncbi.nlm.nih.gov/29955634) | 2 |
|  |  |  |  |  |  | *CG9129* | -5.90 | 0.0001 | *NA* |  |
|  |  |  |  |  |  | *Ppm1* | -5.95 | 0.0008 | [*PPM1A*](https://www.genenames.org/data/gene-symbol-report/#!/hgnc_id/9275) | 5 |
|  |  |  |  |  |  | *CG18662* | -6.01 | 0.0029 | *PHPT1* | 5 |
|  |  |  |  |  |  | *lncRNA:CR9284* | -6.01 | 0.0002 | *NA* |  |
|  |  |  |  |  |  | *mil* | -6.06 | 0.0000 | [*NAP1L1*](https://www.genenames.org/data/gene-symbol-report/#!/hgnc_id/7637) | 7 |
|  |  |  |  |  |  | *CG18568* | -6.07 | 0.0305 | *NA* | NA |
|  |  |  |  |  |  | *lncRNA:CR32652* | -6.27 | 0.0000 | *NA* |  |
|  |  |  |  |  |  | *ProtB* | -6.28 | 0.0001 | *NA* |  |
|  |  |  |  |  |  | *mAcon2* | -6.30 | 0.0323 | *ACO2* | 12 |
|  |  |  |  |  |  | *CG14327* | -6.38 | 0.0006 | *NA* | NA |
|  |  |  |  |  |  | *CG31921* | -6.53 | 0.0038 | *VGF* | 1 |
|  |  |  |  |  |  | *CG42523* | -6.54 | 0.0076 | *NA* | NA |
|  |  |  |  |  |  | *Pen* | -6.57 | 0.0000 | [*KPNA2*](https://www.genenames.org/data/gene-symbol-report/#!/hgnc_id/6395) | 12 |
|  |  |  |  |  |  | *CG31740* | -6.62 | 0.0001 | *NA* | NA |
|  |  |  |  |  |  | *CG10734* | -6.67 | 0.0000 | *NA* | NA |
|  |  |  |  |  |  | *CG43328* | -6.77 | 0.0254 | *MICOS13* | 2 |
|  |  |  |  |  |  | *lncRNA:CR43488* | -6.78 | 0.0388 | *NA* | - |
|  |  |  |  |  |  | *CG3222* | -7.20 | 0.0030 | *MORN5* | 10 |
|  |  |  |  |  |  | *knon* | -7.25 | 0.0005 | *ATP5PD* | 7 |
|  |  |  |  |  |  | *CG17377* | -7.55 | 0.0000 | *KRTAP5-3* | 1 |
|  |  |  |  |  |  | *Mst98Ca* | -7.60 | 0.0000 | *NA* | NA |
|  |  |  |  |  |  | *CG4836* | -7.62 | 0.0000 | [*SORD*](https://www.genenames.org/data/gene-symbol-report/#!/hgnc_id/11184) | 5 |
|  |  |  |  |  |  | *CG33340* | -7.78 | 0.0000 | *NA* | NA |
|  |  |  |  |  |  | *CG43755* | -7.85 | 0.0052 | *KTN1* | 1 |
|  |  |  |  |  |  | *boly* | -7.97 | 0.0195 | *NA* | NA |
|  |  |  |  |  |  | *CG12699* | -8.18 | 0.0000 | *NA* | NA |
|  |  |  |  |  |  | *CG4691* | -8.34 | 0.0000 | *NA* | NA |
|  |  |  |  |  |  | *CG31988* | -8.59 | 0.0000 | [*LPXN*](https://www.genenames.org/data/gene-symbol-report/#!/hgnc_id/14061) | 5 |
|  |  |  |  |  |  | *sinah* | -8.60 | 0.0008 | [*SIAH1*](https://www.genenames.org/data/gene-symbol-report/#!/hgnc_id/10857) | 4 |
|  |  |  |  |  |  | *CG7251* | -8.66 | 0.0028 | *CCDC34* | 3 |
|  |  |  |  |  |  | *Acam* | -8.66 | 0.0180 | *CALM1* | 3 |
|  |  |  |  |  |  | *S-Lap8* | -8.67 | 0.0000 | *LAP3* | 9 |
|  |  |  |  |  |  | *dj* | -9.03 | 0.0000 | *NA* | NA |
|  |  |  |  |  |  | *CG30039* | -9.17 | 0.0000 | *NA* | NA |
|  |  |  |  |  |  | *S-Lap4* | -9.25 | 0.0000 | *LAP3* | 9 |
|  |  |  |  |  |  | *Mst98Cb* | -9.29 | 0.0010 | *NA* | NA |
|  |  |  |  |  |  | *CG5614* | -9.84 | 0.0002 | *FOPNL* | 5 |
|  |  |  |  |  |  | *CG3213* | -10.39 | 0.0000 | [*ODF2*](https://www.genenames.org/data/gene-symbol-report/#!/hgnc_id/8114) | 2 |
|  |  |  |  |  |  | *CG3330* | -10.52 | 0.0000 | *NA* | NA |
|  |  |  |  |  |  | *ssp5* | -11.18 | 0.0000 | *NA* | NA |
|  |  |  |  |  |  | *loopin-1* | -11.56 | 0.0000 | *LAP3* | 9 |
|  |  |  |  |  |  | *CG13991* | -12.70 | 0.0000 | *CCDC38* | 1 |
|  |  |  |  |  |  | *CG9173* | -13.18 | 0.0007 | *NA* | NA |
|  |  |  |  |  |  | *CG1288* | -13.38 | 0.0000 | *NA* | NA |
|  |  |  |  |  |  | *snoRNA:660* | -13.73 | 0.0140 | *NA* | NA |
|  |  |  |  |  |  | *CG8701* | -13.74 | 0.0000 | *NA* | NA |
|  |  |  |  |  |  | *CG3124* | -14.12 | 0.0000 | *PRSS45P* | 2 |
|  |  |  |  |  |  | *HemK2* | -114.60 | 0.0000 | *N6AMT1* | 12 |
