# Supplementary material for: The ortholog of human ssDNA-binding protein SSBP3 influences neurodevelopment and autism-like behaviors in Drosophila melanogaster
Source: PLoS Biol. 2023 Jul 24;21(7):e3002210. doi: 10.1371/journal.pbio.3002210 (PMC10399856; doi:10.1371/journal.pbio.3002210)
Supplement: S3 Table — Down-regulated genes in Ssdp overexpressing flies identified using RNA-seq, with an orthology score ≥5 are presented. The human ortholog, fold change, DIOPT score for these genes are mentioned. Furthermore, the number of patients listed in DECIPHER with variations in these genes that show autistic behavior, micro/macrocephaly, and ID are presented. Evidence of association of these genes with autism, ID, other neurological disorders, and inflammation/immunity response in literature is also listed. (DOCX) [file pbio.3002210.s014.docx]

| **Fly Gene** | **Human Ortholog** | **SSDP/**  **CTL.fc** | **DIOPT Score** | **DECIPHER Patient Variant Numbers** | | | **Gene Association in Literature** | | | | |
| --- | --- | --- | --- | --- | --- | --- | --- | --- | --- | --- | --- |
|  |  |  |  |  |  |  | **Autism** | **Intellectual Disability** | **Other Neurological Disorders** | **Inflammation/Immunity Response** | |
|  |  |  |  | **Autistic Behav.** | **Microcephaly / Macrocephaly** | **Intellectual Disability** |  |  |  |  |  |
| *CG10672* | *DHRS4* | -1.51 | 15 | 3 | 10 | 13 |  |  |  | [1,2] | |
| *Qsox2* | *QSOX2* | -1.52 | 15 | 1 | 20 | 43 |  |  |  | - |  |
| *CG7470* | *ALDH18A1* | -1.59 | 15 | 0 | 6 | 8 | [3] |  |  | [4] |  |
| *CG10361* | *GCAT* | -1.68 | 15 | 2 | 9 | 29 |  |  |  | [5] |  |
| *polo* | [*PLK1*](https://www.genenames.org/data/gene-symbol-report/#!/hgnc_id/9077) | -4.04 | 15 | 4 | 5 | 9 | [6] |  |  | [7,8] |  |
| *Pu* | *GCH1* | -1.54 | 14 | 0 | 4 | 8 | [9] |  |  | [10] |  |
| *CG4335* | *TMLHE* | -1.64 | 14 | 9 | 17 | 35 | [11,12] |  |  | [13] |  |
| *CG16965* | *PGGHG* | -1.76 | 14 | 1 | 21 | 25 |  |  |  | - |  |
| *pr* | *PTS* | -2.03 | 14 | 0 | 44 | 72 | [14] |  |  | [15] |  |
| *Dhc98D* | *DNAH10* | -2.08 | 14 | 2 | 25 | 31 | [16,17] |  |  | [13] |  |
| *se* | *GSTO1* | -2.19 | 14 | 0 | 4 | 3 |  |  | Rett Syndrome [18] | [19] |  |
| *CG3609* | *DHDH* | -1.72 | 13 | 1 | 6 | 8 |  |  |  | [20] |  |
| *FANCI* | *FANCI* | -1.75 | 13 | 1 | 25 | 38 | [21] |  |  |  |  |
| *Amy-p* | *AMY2B* | -1.91 | 13 | 1 | 4 | 9 |  |  |  |  |  |
| *CG8526* | *ASPG* | -2.32 | 13 | 1 | 36 | 51 |  |  |  | [22] |  |
| *Dnah3* | *DNAH3* | -2.34 | 13 | 4 | 5 | 7 |  | [23] |  | [24] |  |
| *Ssl2* | *APMAP* | -2.40 | 13 | 3 | 5 | 4 |  |  |  | [25] |  |
| *CG9314* | *CAT* | -2.82 | 13 | 2 | 8 | 27 | [26], [27] |  |  | [28] |  |
| *CG40486* | *DHRS11* | -1.50 | 12 | 22 | 18 | 64 |  |  |  |  |  |
| *Ada2a* | *TADA2A* | -1.53 | 12 | 22 | 20 | 67 | [29] | [29] |  |  |  |
| *dnd* | *ARL3* | -1.54 | 12 | 0 | 4 | 8 | [30] |  |  | [31] |  |
| *RhoGAP54D* | *ARHGAP19* | -1.62 | 12 | 2 | 7 | 12 | [32] |  |  | [33] |  |
| *primo-1* | *ACP1* | -1.73 | 12 | 4 | 15 | 36 | [34] | [35] |  | [36] |  |
| *Amy-d* | *AMY2B* | -1.75 | 12 | 1 | 4 | 9 | [37] |  |  | [22] |  |
| *Orc1* | *ORC1* | -1.86 | 12 | 0 | 3 | 5 |  | [38] |  | [39] |  |
| *CG11825* | *HIGD1A* | -1.87 | 12 | 0 | 5 | 3 |  |  | [40] | [41] |  |
| *Fuca* | *FUCA2* | -2.45 | 12 | 0 | 4 | 5 | [37] |  |  | [42] |  |
| *CG9920* | *HSPE1* | -3.35 | 12 | 2 | 7 | 23 | [43], [44] |  |  |  |  |
| *CG5280* | *C22orf23* | -3.93 | 12 | 3 | 19 | 28 |  |  | Waardenburg syndrome[45] | [46] |  |
| *mAcon2* | *ACO2* | -6.29 | 12 | 4 | 19 | 33 |  |  |  | [47] |  |
| *Pen* | [*KPNA2*](https://www.genenames.org/data/gene-symbol-report/#!/hgnc_id/6395) | -6.56 | 12 | 1 | 7 | 4 | [48] | [49] | Cerebral Malformations [50] |  |  |
| *HemK2* | *N6AMT1* | -114.59 | 12 | 2 | 9 | 8 | [51] |  | Essential tremor [52] |  |  |
| *CG2121* | *UNC93A* | -1.50 | 11 | 6 | 63 | 57 |  |  |  |  |  |
| *CG31278* | *PDF* | -1.69 | 11 | 2 | 11 | 15 |  |  | [53] |  |  |
| *CG8757* | *DHRS11* | -1.95 | 11 | 22 | 18 | 64 |  | [54] |  |  |  |
| *S-Lap1* | *LAP3* | -2.42 | 11 | 0 | 14 | 27 |  |  |  |  |  |
| *Pgm2b* | *PGM2* | -4.91 | 11 | 0 | 3 | 7 |  |  |  | [55] |  |
| *CG10252* | *ODF3L2* | -5.18 | 11 | 5 | 13 | 14 | [56] |  |  |  |  |
| *CG18302* | *LIPA* | -1.54 | 10 | 5 | 6 | 6 |  |  |  |  |  |
| *dob* | *PNPLA2* | -1.54 | 10 | 1 | 18 | 20 |  |  |  |  |  |
| *ZnT33D* | *SLC30A2* | -2.08 | 10 | 0 | 4 | 1 |  |  | Brain development [57] |  |  |
| *CG5435* | *C1orf194* | -2.24 | 10 | 2 | 3 | 14 | [58] |  |  |  |  |
| *LysX* | *Lyz* | -2.59 | 10 | 1 | 2 | 3 |  |  |  |  |  |
| *gskt* | [*GSK3B*](https://www.genenames.org/data/gene-symbol-report/#!/hgnc_id/4617) | -4.40 | 10 | 3 | 4 | 9 | [59], [60] |  |  |  |  |
| *CG3222* | *MORN5* | -7.19 | 10 | 1 | 6 | 7 |  |  | Embryonic lethal mutation [61] |  |  |
| *Rpp21* | *RPP21* | -1.89 | 9 | 0 | 1 | 2 | [62] |  |  |  |  |
| *betaTub85D* | *TUBB2B* | -2.24 | 9 | 7 | 36 | 53 |  | [63] | [64] |  |  |
| *CG7886* | [*CEP78*](https://www.genenames.org/data/gene-symbol-report/#!/hgnc_id/25740) | -4.50 | 9 | 3 | 3 | 13 | [65] |  | Several Mendelian diseases including NDD [66] |  |  |
| *Nazo* | [*C19orf12*](https://www.genenames.org/data/gene-symbol-report/#!/hgnc_id/25443) | -5.17 | 9 | 2 | 4 | 3 |  |  |  |  |  |
| *S-Lap8* | *LAP3* | -8.66 | 9 | 0 | 14 | 27 | [67] | [68] | Schizophrenia  [69] | [70] |  |
| *S-Lap4* | *LAP3* | -9.24 | 9 | 0 | 14 | 27 | [67] | [68] | Schizophrenia [69] | [70] |  |
| *loopin-1* | *LAP3* | -11.55 | 9 | 0 | 14 | 27 | [67] | [68] | Schizophrenia [69] | [70] |  |
| *CG11619* | *GPCPD1* | -1.66 | 8 | 2 | 6 | 12 |  |  |  |  |  |
| *Cyp9b1* | *CYP3A5* | -1.79 | 8 | 0 | 11 | 12 | [71] |  |  |  |  |
| *Porin2* | *VDAC3* | -2.34 | 8 | 6 | 6 | 11 | [72] |  |  |  |  |
| *fan* | *VAPB* | -3.00 | 8 | 3 | 8 | 20 | [73] |  |  |  |  |
| *alphaTub85E* | *TUBA1A* | -3.20 | 8 | 2 | 17 | 16 | [74] | [75] | Cerebral Palsy, brain malformations[76], [77] |  |  |
| *CG4546* | *CKMT2* | -3.40 | 8 | 2 | 3 | 3 |  |  |  |  |  |
| *CG30203* | *SPON1* | -1.51 | 7 | 1 | 10 | 8 |  |  | Dementia[78] |  |  |
| *CG31075* | *AlDH1A1* | -1.56 | 7 | 4 | 2 | 11 | [79] |  |  |  |  |
| *PGRP-SD* | *PGLYRP1* | -2.07 | 7 | 2 | 6 | 8 |  |  | Neurodevelopment disorders[80] |  |  |
| *CG8908* | *ABCA3* | -2.20 | 7 | 2 | 16 | 31 |  |  |  |  |  |
| *ste24b* | *ZMPSTE24* | -2.95 | 7 | 0 | 3 | 2 |  |  | Progeroid syndrome ,Craniofacial problems [81], [82] | [83] |  |
| *fzo* | [*MFN2*](https://www.genenames.org/data/gene-symbol-report/#!/hgnc_id/16877) | -4.76 | 7 | 3 | 19 | 27 | [84] | [85] | Charcot-Marie-Tooth Disease[86][87] |  |  |
| *mil* | [*NAP1L1*](https://www.genenames.org/data/gene-symbol-report/#!/hgnc_id/7637) | -6.06 | 7 | 0 | 2 | 5 | [88] | [89] | Brain development[90] |  |  |
| *knon* | *ATP5PD* | -7.24 | 7 | 1 | 8 | 15 |  |  |  |  |  |
| *CG13510* | *LITAF* | -1.52 | 6 | 2 | 7 | 14 | [91] | [92], [93] | Neuropathies [94] |  |  |
| *CG7900* | *FAAH2* | -1.69 | 6 | 3 | 5 | 16 | [95] |  |  |  |  |
| *Nepl20* | *KEL* | -1.72 | 6 | 3 | 19 | 20 |  |  |  |  |  |
| *qin* | *TDRD1* | -1.75 | 6 | 3 | 11 | 23 |  |  |  |  |  |
| *CG13168* | *IQCD* | -2.26 | 6 | 0 | 14 | 17 |  |  |  |  |  |
| *CG5653* | *PAOX* | -2.39 | 6 | 3 | 19 | 37 | [96] |  |  |  |  |
| *Cyp6a2* | *CYP3A4* | -2.75 | 6 | 0 | 11 | 13 | [97,98] |  |  | [99] |  |
| *CG4408* | *CPA1* | -3.17 | 6 | 2 | 5 | 11 |  |  |  |  |  |
| *CG18063* | [*SPATA17*](https://www.genenames.org/data/gene-symbol-report/#!/hgnc_id/25184) | -5.55 | 6 | 1 | 2 | 4 |  | [100] | Anorexia Nervosa[101] | [102] |  |
| *CG10345* | *SCARB1* | -1.51 | 5 | 2 | 23 | 31 | [103] |  |  | [104] |  |
| *CG15547* | *NME5* | -1.52 | 5 | 0 | 3 | 9 |  |  |  | [105] |  |
| *ppk29* | *ASIC1* | -1.79 | 5 | 1 | 2 | 4 |  |  |  | [106][107] |  |
| *Cralbp* | *CLVS1* | -1.82 | 5 | 1 | 4 | 7 |  |  | Schizophrenia(Suicidal Ideation), Bipolar Disorder [108] |  |  |
| *CG15539* | *P4HA3* | -1.97 | 5 | 1 | 3 | 4 |  |  |  | [109] |  |
| *CG2955* | *MAPRE3* | -2.98 | 5 | 3 | 6 | 8 |  |  | Cognitive Impairment, Alzheimer’s and Major depressive disorder[110], [111] | [112] |  |
| *scpr-C* | *GLIPR1L1* | -3.87 | 5 | 0 | 2 | 5 | [113] |  |  |  |  |
| *Ppm1* | [*PPM1A*](https://www.genenames.org/data/gene-symbol-report/#!/hgnc_id/9275) | -5.95 | 5 | 0 | 8 | 8 | [114] |  |  | [115] |  |
| *CG18662* | *PHPT1* | -6.00 | 5 | 2 | 9 | 23 | [96] | [116] | Lymphoblastic leukemia, Charcot-Marie-Tooth disease [117] , [118] | [119] |  |
| *CG4836* | [*SORD*](https://www.genenames.org/data/gene-symbol-report/#!/hgnc_id/11184) | -7.61 | 5 | 3 | 4 | 11 |  |  | Axonal hereditary neuropathy [120] | [121] |  |
| *CG31988* | [*LPXN*](https://www.genenames.org/data/gene-symbol-report/#!/hgnc_id/14061) | -8.58 | 5 | 1 | 3 | 3 | [122] |  |  | [123] |  |
| *CG5614* | *FOPNL* | -9.84 | 5 | 45 | 23 | 115 | [124] | [125] |  |  |  |

### References:

1. Li S, Zhu Y, Wei C, Li C, Chen W, Jiang S, et al. Identification of Molecular Correlations Between DHRS4 and Progressive Neurodegeneration in Amyotrophic Lateral Sclerosis By Gene Co-Expression Network Analysis. Front Immunol. 2022;13. doi:10.3389/fimmu.2022.874978

2. Riege K, Hölzer M, Klassert TE, Barth E, Bräuer J, Collatz M, et al. Massive effect on LncRNAs in human monocytes during fungal and bacterial infections and in response to Vitamins A and D. Sci Rep. 2017;7. doi:10.1038/srep40598

3. Jurnak F. The pivotal role of Aldehyde toxicity in autism spectrum disorder: The therapeutic potential of micronutrient supplementation. Nutr Metab Insights. 2016;8. doi:10.4137/NMI.S29531

4. Lin J, Chen YT, Xia J, Yang Q. MiR674 inhibits the neuraminidase-stimulated immune response on dendritic cells via down-regulated Mbnl3. Oncotarget. 2016;7. doi:10.18632/oncotarget.9832

5. Kizil C, Sariya S, Kim YA, Rajabli F, Martin E, Reyes-Dumeyer D, et al. Admixture Mapping of Alzheimer’s disease in Caribbean Hispanics identifies a new locus on 22q13.1. Mol Psychiatry. 2022;27: 2813–2820. doi:10.1038/s41380-022-01526-6

6. Barber JCK, Hall V, Maloney VK, Huang S, Roberts AM, Brady AF, et al. 16p11.2-p12.2 duplication syndrome; A genomic condition differentiated from euchromatic variation of 16p11.2. Eur J Hum Genet. 2013;21. doi:10.1038/ejhg.2012.144

7. Vertii A, Ivshina M, Zimmerman W, Hehnly H, Kant S, Doxsey S. The Centrosome Undergoes Plk1-Independent Interphase Maturation during Inflammation and Mediates Cytokine Release. Dev Cell. 2016;37. doi:10.1016/j.devcel.2016.04.023

8. Zhou J, Yang Q, Lu L, Tuo Z, Shou Z, Cheng J. Plk1 inhibition induces immunogenic cell death and enhances immunity against nsclc. Int J Med Sci. 2021;18. doi:10.7150/ijms.60135

9. Mordaunt CE, Park BY, Bakulski KM, Feinberg JI, Croen LA, Ladd-Acosta C, et al. A meta-analysis of two high-risk prospective cohort studies reveals autism-specific transcriptional changes to chromatin, autoimmune, and environmental response genes in umbilical cord blood. Mol Autism. 2019;10. doi:10.1186/s13229-019-0287-z

10. Larbalestier H, Keatinge M, Watson L, White E, Gowda S, Wei W, et al. GCH1 Deficiency Activates Brain Innate Immune Response and Impairs Tyrosine Hydroxylase Homeostasis. J Neurosci. 2022;42. doi:10.1523/JNEUROSCI.0653-21.2021

11. Celestino-Soper PBS, Shaw CA, Sanders SJ, Li J, Murtha MT, Gulhan Ercan-Sencicek A, et al. Use of array CGH to detect exonic copy number variants throughout the genome in autism families detects a novel deletion in TMLHE. Hum Mol Genet. 2011;20. doi:10.1093/hmg/ddr363

12. Nava C, Lamari F, Héron D, Mignot C, Rastetter A, Keren B, et al. Analysis of the chromosome X exome in patients with autism spectrum disorders identified novel candidate genes, including TMLHE. Transl Psychiatry. 2012;2. doi:10.1038/tp.2012.102

13. Rose S, Niyazov DM, Rossignol DA, Goldenthal M, Kahler SG, Frye RE. Clinical and Molecular Characteristics of Mitochondrial Dysfunction in Autism Spectrum Disorder. Molecular Diagnosis and Therapy. 2018. doi:10.1007/s40291-018-0352-x

14. Anderson BM, Schnetz-Boutaud N, Bartlett J, Wright HH, Abramson RK, Cuccaro ML, et al. Examination of association to autism of common genetic variationin genes related to dopamine. Autism Res. 2008;1. doi:10.1002/aur.55

15. Haider AS, Cardinale IR, Whynot JA, Krueger JG. Effects of etanercept are distinct from infliximab in modulating proinflammatory genes in activated human leukocytes. J Investig Dermatology Symp Proc. 2007;12. doi:10.1038/sj.jidsymp.5650032

16. De Rubeis S, He X, Goldberg AP, Poultney CS, Samocha K, Cicek AE, et al. Synaptic, transcriptional and chromatin genes disrupted in autism. Nature. 2014;515. doi:10.1038/nature13772

17. Krupp DR, Barnard RA, Duffourd Y, Evans SA, Mulqueen RM, Bernier R, et al. Exonic Mosaic Mutations Contribute Risk for Autism Spectrum Disorder. Am J Hum Genet. 2017;101. doi:10.1016/j.ajhg.2017.07.016

18. Pecorelli A, Leoni G, Cervellati F, Canali R, Signorini C, Leoncini S, et al. Genes related to mitochondrial functions, protein degradation, and chromatin folding are differentially expressed in lymphomonocytes of rett syndrome patients. Mediators Inflamm. 2013;2013. doi:10.1155/2013/137629

19. Menon D, Innes A, Oakley AJ, Dahlstrom JE, Jensen LM, Brüstle A, et al. GSTO1-1 plays a pro-inflammatory role in models of inflammation, colitis and obesity. Sci Rep. 2017;7. doi:10.1038/s41598-017-17861-6

20. Morgan MJ, Liu ZG. Crosstalk of reactive oxygen species and NF-κB signaling. Cell Research. 2011. doi:10.1038/cr.2010.178

21. Ionita-Laza I, Xu B, Makarov V, Buxbaum JD, Roos JL, Gogos JA, et al. Scan statistic-based analysis of exome sequencing data identifies FAN1 at 15q13.3 as a susceptibility gene for schizophrenia and autism. Proc Natl Acad Sci U S A. 2014;111. doi:10.1073/pnas.1309475110

22. Elhag DA, Kumar M, Khodor S Al. Exploring the triple interaction between the host genome, the epigenome, and the gut microbiome in type 1 diabetes. International Journal of Molecular Sciences. 2021. doi:10.3390/ijms22010125

23. Järvelä I, Määttä T, Acharya A, Leppälä J, Jhangiani SN, Arvio M, et al. Exome sequencing reveals predominantly de novo variants in disorders with intellectual disability (ID) in the founder population of Finland. Hum Genet. 2021;140. doi:10.1007/s00439-021-02268-1

24. Li N, Li B, Zhan X. Comprehensive Analysis of Tumor Microenvironment Identified Prognostic Immune-Related Gene Signature in Ovarian Cancer. Front Genet. 2021;12. doi:10.3389/fgene.2021.616073

25. Suttapitugsakul S, Tong M, Wu R. Time-Resolved and Comprehensive Analysis of Surface Glycoproteins Reveals Distinct Responses of Monocytes and Macrophages to Bacterial Infection. Angew Chemie - Int Ed. 2021;60. doi:10.1002/anie.202102692

26. Burke SL, Cobb J, Agarwal R, Maddux M, Cooke MS. How Robust is the Evidence for a Role of Oxidative Stress in Autism Spectrum Disorders and Intellectual Disabilities? J Autism Dev Disord. 2021;51. doi:10.1007/s10803-020-04611-3

27. Ranjbar A, Rashedi V, Rezaei M. Comparison of urinary oxidative biomarkers in Iranian children with autism. Research in Developmental Disabilities. 2014. doi:10.1016/j.ridd.2014.07.010

28. Shi X, Shi Z, Huang H, Zhu H, Zhou P, Zhu H, et al. Ability of recombinant human catalase to suppress inflammation of the murine lung induced by influenza A. Inflammation. 2014;37. doi:10.1007/s10753-013-9800-2

29. Milone R, Tancredi R, Cosenza A, Ferrari AR, Scalise R, Cioni G, et al. 17q12 recurrent deletions and duplications: Description of a case series with neuropsychiatric phenotype. Genes (Basel). 2021;12. doi:10.3390/genes12111660

30. D’Aurizio R, Catona O, Pitasi M, Li YE, Ren B, Nicolis SK. Bridging between Mouse and Human Enhancer-Promoter Long-Range Interactions in Neural Stem Cells, to Understand Enhancer Function in Neurodevelopmental Disease. Int J Mol Sci. 2022;23. doi:10.3390/ijms23147964

31. Powell L, Samarakoon YH, Ismail S, Sayer JA. ARL3, a small GTPase with a functionally conserved role in primary cilia and immune synapses. Small GTPases. 2021. doi:10.1080/21541248.2019.1703466

32. Hashimoto R, Nakazawa T, Tsurusaki Y, Yasuda Y, Nagayasu K, Matsumura K, et al. Whole-exome sequencing and neurite outgrowth analysis in autism spectrum disorder. J Hum Genet. 2016;61. doi:10.1038/jhg.2015.141

33. He H, Huang J, Wu S, Jiang S, Liang L, Liu Y, et al. The roles of GTPase-activating proteins in regulated cell death and tumor immunity. Journal of Hematology and Oncology. 2021. doi:10.1186/s13045-021-01184-1

34. Anne Spence M, Ritvo ER, Marazita ML, Funderburk SJ, Sparkes RS, Freeman BJ. Gene mapping studies with the syndrome of autism. Behav Genet. 1985;15. doi:10.1007/BF01071928

35. Doco-Fenzy M, Leroy C, Schneider A, Petit F, Delrue MA, Andrieux J, et al. Early-onset obesity and paternal 2pter deletion encompassing the ACP1, TMEM18, and MYT1L genes. Eur J Hum Genet. 2014;22. doi:10.1038/ejhg.2013.189

36. Gansburgskii AN, Pavlov A V. Proliferative properties of cell differons in the vascular wall. Morfologiia. 1998;113.

37. Liu W, Li L, Xia X, Zhou X, Du Y, Yin Z, et al. Integration of Urine Proteomic and Metabolomic Profiling Reveals Novel Insights Into Neuroinflammation in Autism Spectrum Disorder. Front Psychiatry. 2022;13. doi:10.3389/fpsyt.2022.780747

38. Zu G, Liu Y, Cao J, Zhao B, Zhang H, You L. BRPF1-KAT6A/KAT6B Complex: Molecular Structure, Biological Function and Human Disease. Cancers. MDPI; 2022. doi:10.3390/cancers14174068

39. Costa Svedman F, Das I, Tuominen R, Darai Ramqvist E, Höiom V, Egyhazi Brage S. Proliferation and Immune Response Gene Signatures Associated with Clinical Outcome to Immunotherapy and Targeted Therapy in Metastatic Cutaneous Malignant Melanoma. Cancers (Basel). 2022;14. doi:10.3390/cancers14153587

40. Hayashi T, Asano Y, Shintani Y, Aoyama H, Kioka H, Tsukamoto O, et al. Higd1a is a positive regulator of cytochrome c oxidase. Proc Natl Acad Sci U S A. 2015;112. doi:10.1073/pnas.1419767112

41. Xia J, Kong L, Zhou LJ, Wu SZ, Yao LJ, He C, et al. Genome-Wide bimolecular fluorescence complementation-based proteomic analysis of Toxoplasma gondii ROP18’s human interactome shows its key role in regulation of cell immunity and apoptosis. Front Immunol. 2018;9. doi:10.3389/fimmu.2018.00061

42. Ackerman ME, Crispin M, Yu X, Baruah K, Boesch AW, Harvey DJ, et al. Natural variation in Fc glycosylation of HIV-specific antibodies impacts antiviral activity. J Clin Invest. 2013;123. doi:10.1172/JCI65708

43. Kim JJ, Mandelli L, Lim S, Lim HK, Kwon OJ, Pae CU, et al. Association analysis of heat shock protein 70 gene polymorphisms in schizophrenia. Eur Arch Psychiatry Clin Neurosci. 2008;258. doi:10.1007/s00406-007-0791-6

44. Guan J, Cai JJ, Ji G, Sham PC. Commonality in dysregulated expression of gene sets in cortical brains of individuals with autism, schizophrenia, and bipolar disorder. Transl Psychiatry. 2019;9. doi:10.1038/s41398-019-0488-4

45. Jelena B, Christina L, Eric V, Fabiola QR. Phenotypic variability in Waardenburg syndrome resulting from a 22q12.3-q13.1 microdeletion involving SOX10. Am J Med Genet Part A. 2014;164. doi:10.1002/ajmg.a.36446

46. Calandra T, Roger T. Macrophage migration inhibitory factor: A regulator of innate immunity. Nature Reviews Immunology. 2003. doi:10.1038/nri1200

47. Williams NC, O’Neill LAJ. A role for the krebs cycle intermediate citrate in metabolic reprogramming in innate immunity and inflammation. Frontiers in Immunology. 2018. doi:10.3389/fimmu.2018.00141

48. Rai-Bhogal R, Ahmad E, Li H, Crawford DA. Microarray analysis of gene expression in the cyclooxygenase knockout mice – a connection to autism spectrum disorder. Eur J Neurosci. 2018;47. doi:10.1111/ejn.13781

49. Vergult S, Dauber A, Chiaie BD, Van Oudenhove E, Simon M, Rihani A, et al. 17q24.2 microdeletions: A new syndromal entity with intellectual disability, truncal obesity, mood swings and hallucinations. Eur J Hum Genet. 2012;20. doi:10.1038/ejhg.2011.239

50. Paciorkowski AR, Weisenberg J, Kelley JB, Spencer A, Tuttle E, Ghoneim D, et al. Autosomal recessive mutations in nuclear transport factor KPNA7 are associated with infantile spasms and cerebellar malformation. Eur J Hum Genet. 2014;22. doi:10.1038/ejhg.2013.196

51. Williams EL, Casanova MF, Switala AE, Li H, Qiu M. Transposable elements occur more frequently in autism-risk genes: Implications for the role of genomic instability in autism. Transl Neurosci. 2013;4. doi:10.2478/s13380-013-0113-6

52. Clark LN, Gao Y, Wang GT, Hernandez N, Ashley-Koch A, Jankovic J, et al. Whole genome sequencing identifies candidate genes for familial essential tremor and reveals biological pathways implicated in essential tremor aetiology. eBioMedicine. 2022;85: 104290. doi:10.1016/j

53. Lee MD, She Y, Soskis MJ, Borella CP, Gardner JR, Hayes PA, et al. Human mitochondrial peptide deformylase, a new anticancer target of actinonin-based antibiotics. J Clin Invest. 2004;114. doi:10.1172/JCI200422269

54. Merikangas AK, Shelly M, Knighton A, Kotler N, Tanenbaum N, Almasy L. What genes are differentially expressed in individuals with schizophrenia? A systematic review. Molecular Psychiatry. 2022. doi:10.1038/s41380-021-01420-7

55. Hecker J, Chun S, Samiei A, Liu C, Laurie C, Kachroo P, et al. *FGF20* and *PGM2* variants are associated with childhood asthma in family-based whole-genome sequencing studies. Hum Mol Genet. 2022. doi:10.1093/hmg/ddac258

56. Salyakina D, Ma DQ, Jaworski JM, Konidari I, Whitehead PL, Henson R, et al. Variants in several genomic regions associated with asperger disorder. Autism Res. 2010;3. doi:10.1002/aur.158

57. Willekens J, Runnels LW. Impact of Zinc Transport Mechanisms on Embryonic and Brain Development. Nutrients. MDPI; 2022. doi:10.3390/nu14122526

58. Quartier A, Chatrousse L, Redin C, Keime C, Haumesser N, Maglott-Roth A, et al. Genes and Pathways Regulated by Androgens in Human Neural Cells, Potential Candidates for the Male Excess in Autism Spectrum Disorder. Biol Psychiatry. 2018;84. doi:10.1016/j.biopsych.2018.01.002

59. Mines MA, Yuskaitis CJ, King MK, Beurel E, Jope RS. GSK3 influences social preference and anxiety-related behaviors during social interaction in a mouse model of fragile X syndrome and autism. PLoS One. 2010;5. doi:10.1371/journal.pone.0009706

60. Hamanaka K, Miyake N, Mizuguchi T, Miyatake S, Uchiyama Y, Tsuchida N, et al. Large-scale discovery of novel neurodevelopmental disorder-related genes through a unified analysis of single-nucleotide and copy number variants. Genome Med. 2022;14. doi:10.1186/s13073-022-01042-w

61. Kline J, Vardarajan B, Abhyankar A, Kytömaa S, Levin B, Sobreira N, et al. Embryonic lethal genetic variants and chromosomally normal pregnancy loss. Fertil Steril. 2021;116. doi:10.1016/j.fertnstert.2021.06.039

62. Mellios N, Sur M. The emerging role of microRNAs in schizophrenia and autism spectrum disorders. Frontiers in Psychiatry. 2012. doi:10.3389/fpsyt.2012.00039

63. Kumar R, Corbett MA, Van Bon BWM, Gardner A, A.Woenig J, Jolly LA, et al. Increased STAG2 dosage defines a novel cohesinopathy with intellectual disability and behavioral problems. Hum Mol Genet. 2015;24. doi:10.1093/hmg/ddv414

64. Lasser M, Tiber J, Lowery LA. The role of the microtubule cytoskeleton in neurodevelopmental disorders. Frontiers in Cellular Neuroscience. 2018. doi:10.3389/fncel.2018.00165

65. Patowary A, Won SY, Oh SJ, Nesbitt RR, Archer M, Nickerson D, et al. Family-based exome sequencing and case-control analysis implicate CEP41 as an ASD gene. Transl Psychiatry. 2019;9. doi:10.1038/s41398-018-0343-z

66. Sanchis-Juan A, Stephens J, French CE, Gleadall N, Mégy K, Penkett C, et al. Complex structural variants in Mendelian disorders: identification and breakpoint resolution using short- and long-read genome sequencing. Genome Med. 2018;10. doi:10.1186/s13073-018-0606-6

67. Toma C, Torrico B, Hervás A, Valdés-Mas R, Tristán-Noguero A, Padillo V, et al. Exome sequencing in multiplex autism families suggests a major role for heterozygous truncating mutations. Mol Psychiatry. 2014;19. doi:10.1038/mp.2013.106

68. Alesi V, Barrano G, Morara S, Darelli D, Petrilli K, Capalbo A, et al. A previously undescribed de novo 4p15 deletion in a patient with apparently isolated metopic craniosynostosis. Am J Med Genet Part A. 2011;155. doi:10.1002/ajmg.a.34201

69. Hjelm BE, Rollins B, Mamdani F, Lauterborn JC, Kirov G, Lynch G, et al. Evidence of Mitochondrial Dysfunction within the Complex Genetic Etiology of Schizophrenia. Complex Psychiatry. 2015;1. doi:10.1159/000441252

70. Rutgeerts P, Vermeire S, Van Assche G. Biological Therapies for Inflammatory Bowel Diseases. Gastroenterology. 2009. doi:10.1053/j.gastro.2009.02.001

71. Nezgovorova V, Ferretti CJ, Taylor BP, Shanahan E, Uzunova G, Hong K, et al. Potential of cannabinoids as treatments for autism spectrum disorders. Journal of Psychiatric Research. 2021. doi:10.1016/j.jpsychires.2021.02.048

72. Masini E, Loi E, Vega-Benedetti AF, Carta M, Doneddu G, Fadda R, et al. An overview of the main genetic, epigenetic and environmental factors involved in autism spectrum disorder focusing on synaptic activity. International Journal of Molecular Sciences. 2020. doi:10.3390/ijms21218290

73. Rojas-Charry L, Nardi L, Methner A, Schmeisser MJ. Abnormalities of synaptic mitochondria in autism spectrum disorder and related neurodevelopmental disorders. Journal of Molecular Medicine. 2021. doi:10.1007/s00109-020-02018-2

74. Willsey J, Yamrom B, Lee Y, Grabowska E, Dalkic E, Rosenbaum J, et al. The contribution of de novo coding mutations to autism spectrum disorder. Nature. 2014.

75. Valentino F, Bruno LP, Doddato G, Giliberti A, Tita R, Resciniti S, et al. Exome sequencing in 200 intellectual disability/autistic patients: New candidates and atypical presentations. Brain Sci. 2021;11. doi:10.3390/brainsci11070936

76. Fahey MC, Maclennan AH, Kretzschmar D, Gecz J, Kruer MC. The genetic basis of cerebral palsy. Developmental Medicine and Child Neurology. 2017. doi:10.1111/dmcn.13363

77. Gardner JF, Cushion TD, Niotakis G, Olson HE, Grant PE, Scott RH, et al. Clinical and functional characterization of the recurrent TUBA1A p.(ARG2HIS) mutation. Brain Sci. 2018;8. doi:10.3390/brainsci8080145

78. Jahanshad N, Rajagopalan P, Hua X, Hibar DP, Nir TM, Toga AW, et al. Genome-wide scan of healthy human connectome discovers SPON1 gene variant influencing dementia severity. Proc Natl Acad Sci U S A. 2013;110. doi:10.1073/pnas.1216206110

79. Alhusaini A, Sarawi W, Mattar D, Abo-Hamad A, Almogren R, Alhumaidan S, et al. Acetyl-L-carnitine and/or liposomal co-enzyme Q10 prevent propionic acid-induced neurotoxicity by modulating oxidative tissue injury, inflammation, and ALDH1A1-RA-RARα signaling in rats. Biomed Pharmacother. 2022;153. doi:10.1016/j.biopha.2022.113360

80. Gonzalez-Santana A, Diaz Heijtz R. Bacterial Peptidoglycans from Microbiota in Neurodevelopment and Behavior. Trends in Molecular Medicine. 2020. doi:10.1016/j.molmed.2020.05.003

81. Akinci B, Sankella S, Gilpin C, Ozono K, Garg A, Agarwal AK. Progeroid syndrome patients with ZMPSTE24 deficiency could benefit when treated with rapamycin and dimethylsulfoxide. Mol Case Stud. 2017;3. doi:10.1101/mcs.a001339

82. Ahmad Z, Zackai E, Medne L, Garg A. Early onset mandibuloacral dysplasia due to compound heterozygous mutations in ZMPSTE24. Am J Med Genet Part A. 2010;152. doi:10.1002/ajmg.a.33664

83. Messner M, Ghadge SK, Maurer T, Graber M, Staggl S, Christine Maier S, et al. ZMPSTE24 Is Associated with Elevated Inflammation and Progerin mRNA. Cells. 2020;9. doi:10.3390/cells9091981

84. Citrigno L, Muglia M, Qualtieri A, Spadafora P, Cavalcanti F, Pioggia G, et al. The mitochondrial dysfunction hypothesis in autism spectrum disorders: Current status and future perspectives. International Journal of Molecular Sciences. 2020. doi:10.3390/ijms21165785

85. Madrid R, Guariglia SR, Haworth A, Korosh W, Gavin M, Lyon GJ. Early-onset cerebellar ataxia in a patient with CMT2A2. Cold Spring Harb Mol Case Stud. 2020;6. doi:10.1101/MCS.A005108

86. Bernard-Marissal N, Van Hameren G, Juneja M, Pellegrino C, Louhivuori L, Bartesaghi L, et al. Altered interplay between endoplasmic reticulum and mitochondria in Charcot-Marie-Tooth type 2A neuropathy. Proc Natl Acad Sci U S A. 2019;116. doi:10.1073/pnas.1810932116

87. Stuppia G, Rizzo F, Riboldi G, Del Bo R, Nizzardo M, Simone C, et al. MFN2-related neuropathies: Clinical features, molecular pathogenesis and therapeutic perspectives. Journal of the Neurological Sciences. 2015. doi:10.1016/j.jns.2015.05.033

88. Gao H, Zhong J, Huang Q, Wu X, Mo X, Lu L, et al. Integrated Systems Analysis Explores Dysfunctional Molecular Modules and Regulatory Factors in Children with Autism Spectrum Disorder. J Mol Neurosci. 2021;71. doi:10.1007/s12031-020-01658-w

89. Buczyńska A, Sidorkiewicz I, Trochimiuk A, Ławicki S, Krętowski AJ, Zbucka‐krętowska M. Novel approaches to an integrated route for trisomy 21 evaluation. Biomolecules. 2021. doi:10.3390/biom11091328

90. Qiao H, Li Y, Feng C, Duo S, Ji F, Jiao J. Nap1l1 Controls Embryonic Neural Progenitor Cell Proliferation and Differentiation in the Developing Brain. Cell Rep. 2018;22. doi:10.1016/j.celrep.2018.02.019

91. Smedler E, Kleppe J, Neufeld J, Lundin K, Bölte S, Landén M. Cerebrospinal fluid and serum protein markers in autism: A co-twin study. J Neurochem. 2021;158. doi:10.1111/jnc.15338

92. Tassano E, Alpigiani MG, Calcagno A, Salvati P, De Miglio L, Fiorio P, et al. Clinical and molecular delineation of a 16p13.2p13.13 microduplication. Eur J Med Genet. 2015;58. doi:10.1016/j.ejmg.2014.12.016

93. Micleaa D, Al-Khzouza C, Osan S, Bucerzan S, Cret V, Popp RA, et al. Genomic study via chromosomal microarray analysis in a group of Romanian patients with obesity and developmental disability/intellectual disability. J Pediatr Endocrinol Metab. 2019;32. doi:10.1515/jpem-2018-0439

94. Sharma S, Govindaraj P, Chickabasaviah Y, Siram R, Shroti A, Seshagiri D, et al. Genetic spectrum of inherited neuropathies in India. Ann Indian Acad Neurol. 2022;25: 407. doi:10.4103/aian.aian_269_22

95. Sirrs S, Van Karnebeek CDM, Peng X, Shyr C, Tarailo-Graovac M, Mandal R, et al. Defects in fatty acid amide hydrolase 2 in a male with neurologic and psychiatric symptoms. Orphanet J Rare Dis. 2015;10. doi:10.1186/s13023-015-0248-3

96. Martin J, Cooper M, Hamshere ML, Pocklington A, Scherer SW, Kent L, et al. Biological overlap of attention-deficit/hyperactivity disorder and autism spectrum disorder: Evidence from copy number variants. J Am Acad Child Adolesc Psychiatry. 2014;53. doi:10.1016/j.jaac.2014.03.004

97. LS C. Cariprazine in Autism Spectrum Disorder and Intellectual Disability Disorder. Glob J Intellect Dev Disabil. 2019;6. doi:10.19080/gjidd.2019.06.555694

98. Erickson CA, Stigler KA, Posey DJ, McDougle CJ. Aripiprazole in autism spectrum disorders and fragile X syndrome. Neurotherapeutics. 2010;7. doi:10.1016/j.nurt.2010.04.001

99. Wollmann BM, Watterdal Syversen S, Vistnes M, Lie E, Mehus LL, Molden E. Associations between cytokine levels and CYP3A4 phenotype in patients with rheumatoid arthritis. Drug Metab Dispos. 2018;46. doi:10.1124/dmd.118.082065

100. Bednarczyk D, Sasiadek MM, Smigiel R. Chromosome aberrations and gene mutations in patients with esophageal atresia. Journal of Pediatric Gastroenterology and Nutrition. 2013. doi:10.1097/MPG.0b013e3182a373dc

101. Brandys MK, De Kovel CGF, Kas MJ, Van Elburg AA, Adan RAH. Overview of genetic research in anorexia nervosa: The past, the present and the future. Int J Eat Disord. 2015;48. doi:10.1002/eat.22400

102. Legrand JMD, Roy E, Baz B, Mukhopadhyay P, Wong HY, Ram R, et al. Genetic variation in the mitogen-activated protein kinase/extracellular signal-regulated kinase pathway affects contact hypersensitivity responses. J Allergy Clin Immunol. 2018;142. doi:10.1016/j.jaci.2018.04.028

103. Hu VW, Nguyen AT, Kim KS, Steinberg ME, Sarachana T, Scully MA, et al. Gene expression profiling of lymphoblasts from autistic and nonaffected sib pairs: Altered pathways in neuronal development and steroid biosynthesis. PLoS One. 2009;4. doi:10.1371/journal.pone.0005775

104. Sadeghi S, Davari M, Asli E, Gharibzadeh S, Vaziri F, Jamnani FR, et al. Effect of IL15 rs10833 and SCARB1 rs10846744 on virologic responses in chronic hepatitis C patients treated with pegylated interferon-α and ribavirin. Gene. 2017;630. doi:10.1016/j.gene.2017.08.005

105. Richetto J, Chesters R, Cattaneo A, Labouesse MA, Gutierrez AMC, Wood TC, et al. Genome-Wide Transcriptional Profiling and Structural Magnetic Resonance Imaging in the Maternal Immune Activation Model of Neurodevelopmental Disorders. Cereb Cortex. 2017;27. doi:10.1093/cercor/bhw320

106. Friese MA, Craner MJ, Etzensperger R, Vergo S, Wemmie JA, Welsh MJ, et al. Acid-sensing ion channel-1 contributes to axonal degeneration in autoimmune inflammation of the central nervous system. Nat Med. 2007;13. doi:10.1038/nm1668

107. Kong X, Tang X, Du W, Tong J, Yan Y, Zheng F, et al. Extracellular acidosis modulates the endocytosis and maturation of macrophages. Cell Immunol. 2013;281. doi:10.1016/j.cellimm.2012.12.009

108. Corponi F, Bonassi S, Vieta E, Albani D, Frustaci A, Ducci G, et al. Genetic basis of psychopathological dimensions shared between schizophrenia and bipolar disorder. Prog Neuro-Psychopharmacology Biol Psychiatry. 2019;89. doi:10.1016/j.pnpbp.2018.08.023

109. Yu C, You M, Zhang P, Zhang S, Yin Y, Zhang X. A five-gene signature is a prognostic biomarker in pan-cancer and related with immunologically associated extracellular matrix. Cancer Med. 2021;10. doi:10.1002/cam4.3986

110. Li S ming, Li B, Zhang L, Zhang G fen, Sun J, Ji M huo, et al. A complement-microglial axis driving inhibitory synapse related protein loss might contribute to systemic inflammation-induced cognitive impairment. Int Immunopharmacol. 2020;87. doi:10.1016/j.intimp.2020.106814

111. Cheng Y, Sun M, Wang F, Geng X, Wang F. Identification of Hub Genes Related to Alzheimer’s Disease and Major Depressive Disorder. Am J Alzheimers Dis Other Demen. 2021;36. doi:10.1177/15333175211046123

112. Austbø L, Kampmann A, Müller-Ladner U, Neumann E, Olsaker I, Skretting G. Identification of differentially expressed genes in ileal Peyer’s patch of scrapie-infected sheep using RNA arbitrarily primed PCR. BMC Vet Res. 2008;4. doi:10.1186/1746-6148-4-12

113. Kuo PH, Chuang LC, Su MH, Chen CH, Chen CH, Wu JY, et al. Genome-wide association study for autism spectrum disorder in taiwanese han population. PLoS One. 2015;10. doi:10.1371/journal.pone.0138695

114. Nishimura Y, Martin CL, Vazquez-Lopez A, Spence SJ, Alvarez-Retuerto AI, Sigman M, et al. Genome-wide expression profiling of lymphoblastoid cell lines distinguishes different forms of autism and reveals shared pathways. Hum Mol Genet. 2007;16. doi:10.1093/hmg/ddm116

115. Lee B, Song YS, Rhodes C, Goh TS, Roh JS, Jeong H, et al. Protein phosphatase magnesium-dependent 1A induces inflammation in rheumatoid arthritis. Biochem Biophys Res Commun. 2020;522. doi:10.1016/j.bbrc.2019.11.112

116. Hamdan N, Mehawej C, Sebaaly G, Jalkh N, Corbani S, Abou-Ghoch J, et al. A homozygous stop gain mutation in BOD1 gene in a Lebanese patient with syndromic intellectual disability. Clin Genet. 2020;98. doi:10.1111/cge.13799

117. van Vlierberghe P, Meijerink JPP, Lee C, Ferrando AA, Look AT, van Wering ER, et al. A new recurrent 9q34 duplication in pediatric T-cell acute lymphoblastic leukemia. Leukemia. 2006;20. doi:10.1038/sj.leu.2404247

118. Yuan RY, Ye ZL, Zhang XR, Xu LQ, He J. Evaluation of SORD mutations as a novel cause of Charcot-Marie-Tooth disease. Ann Clin Transl Neurol. 2021;8. doi:10.1002/acn3.51268

119. Sekar S, McDonald J, Cuyugan L, Aldrich J, Kurdoglu A, Adkins J, et al. Alzheimer’s disease is associated with altered expression of genes involved in immune response and mitochondrial processes in astrocytes. Neurobiol Aging. 2015;36. doi:10.1016/j.neurobiolaging.2014.09.027

120. Laššuthová P, Mazanec R, Staněk D, Sedláčková L, Plevová B, Haberlová J, et al. Biallelic variants in the SORD gene are one of the most common causes of hereditary neuropathy among Czech patients. Sci Rep. 2021;11. doi:10.1038/s41598-021-86857-0

121. Chew V, Lam KP. Leupaxin negatively regulates B cell receptor signaling. J Biol Chem. 2007;282. doi:10.1074/jbc.M704625200

122. Coşkunpınar E, Bozdağı Günal Ö. The Study of Whole Genome Sequencing in Monozygotic Twins with Autism Spectrum Disorder. Experimed. 2022;12: 49–60. doi:10.26650/experimed.1067169

123. Drobin K, Assadi G, Hong MG, Andersson E, Fredolini C, Forsström B, et al. Targeted Analysis of Serum Proteins Encoded at Known Inflammatory Bowel Disease Risk Loci. Inflamm Bowel Dis. 2019;25. doi:10.1093/ibd/izy326

124. Ünsel Bolat G, Bolat H. The Role of Copy Number Variations and FHIT Gene on Phenotypic Characteristics of Cases Diagnosed with Autism Spectrum Disorder. Mol Syndromol. 2021;12. doi:10.1159/000512171

125. Türkyllmaz A, Geckinli BB, Tekin E, Ates EA, Yarali O, Cebi AH, et al. Array-Based Comparative Genomic Hybridization Analysis in Children with Developmental Delay/Intellectual Disability. Balk J Med Genet. 2021;24: 15–24. doi:10.2478/bjmg-2021-0020
