# Supplementary material for: The ortholog of human ssDNA-binding protein SSBP3 influences neurodevelopment and autism-like behaviors in Drosophila melanogaster
Source: PLoS Biol. 2023 Jul 24;21(7):e3002210. doi: 10.1371/journal.pbio.3002210 (PMC10399856; doi:10.1371/journal.pbio.3002210)
Supplement: S4 Table — Up-regulated genes in Ssdp overexpressing flies identified using RNA-seq, with an orthology score ≥5 are presented. The human ortholog, fold change, DIOPT score for these genes are mentioned. Furthermore, the number of patients listed in DECIPHER with variations in these genes that show autistic behavior, micro/macrocephaly, and ID are presented. Evidence of association of these genes with autism, ID, other neurological disorders, and inflammation/immunity response in literature is also listed. (DOCX) [file pbio.3002210.s015.docx]

| **Fly Gene** | **Human Ortholog** | **SSDP/**  **CTL.fc** | **DIOPT Score** | **DECIPHER Patient Variant Numbers** | | | **Gene Association in Literature** | | | |
| --- | --- | --- | --- | --- | --- | --- | --- | --- | --- | --- |
|  |  |  |  |  |  |  | **Autism** | **Intellectual Disability** | **Other Neurological Disorders** | **Inflammation/Immunity Response** |
|  |  |  |  | **Autistic Behav.** | **Microcephaly / Macrocephaly** | **Intellectual Disability** |  |  |  |  |
| *Pepck2* | *PCK2* | 2.08 | 14 | 1 | 10 | 1 | [1] |  |  | [2] |
| *CNT2* | *SLC28A2* | 3.37 | 14 | 1 | 5 | 1 | [3,4] |  |  | [5] |
| *janA* | *PHPT1* | 1.85 | 13 | 2 | 9 | 23 | [6] |  |  |  |
| *Ctl2* | *SLC44A2* | 1.70 | 13 | 3 | 7 | 10 | [7] |  |  | [8] |
| *Hr38* | *NR4A2* | 1.60 | 13 | 3 | 4 | 18 | [9] |  |  | [10,11] |
| *CG11668* | *THEM6* | 1.51 | 13 | 1 | 9 | 29 | [12] |  |  | [13] |
| *Unc-115a* | *ABLIM1* | 1.55 | 13 | 3 | 10 | 23 | [14] |  |  |  |
| *ND2* | *ND2* | 1.93 | 12 |  |  |  | [15] |  |  | [16] |
| *Kif3C* | *KIF17* | 1.68 | 12 | 3 | 5 | 7 | [17] |  | Schizophrenia [18] |  |
| *BBOX1* | *BBOX1* | 1.75 | 12 | 2 | 5 | 21 | [19] |  | Schizophrenia [20] |  |
| *ND3* | *ND3* | 1.85 | 11 |  |  |  | [21–23] |  |  | [16] |
| *CG7255* | *SLC7A2* | 1.52 | 9 | 2 | 11 | 31 | [24] |  |  | [25] |
| *phr6-4* | *CRY2* | 2.01 | 8 | 3 | 5 | 19 | [26] |  |  | [27,28] |
| *Tsp29Fa* | *CD63* | 2.11 | 8 | 1 | 1 | 5 | [29] |  |  | [30,31] |
| *Ugt35C1* | *UGT2B15* | 1.98 | 8 | 1 | 3 | 8 | [32] |  |  |  |
| *Cyp9c1* | *CYP3A4* | 2.23 | 8 | 0 | 11 | 13 | [33,34] |  |  | [35] |
| *Unc-115b* | *ABLIM1* | 1.79 | 8 | 3 | 10 | 23 | [14] |  |  |  |
| *Hsp23* | *CRYAB* | 1.74 | 7 | 0 | 44 | 71 | [36] |  |  | [37,38] |
| *Cyp6w1* | *CYP3A4* | 3.69 | 7 | 0 | 11 | 13 | [33,34] |  |  | [35] |
| *ninaD* | *SCARB1* | 3.45 | 6 | 2 | 23 | 31 | [39] |  |  |  |
| *Cyp4e3* | *CYP4V2* | 1.98 | 6 | 6 | 35 | 69 | [40] | [41] |  | [42] |
| *CG7054* | *PEBP1* | 1.92 | 6 | 0 | 16 | 19 |  |  |  | [43] |
| *sr* | *EGR2* | 1.56 | 6 | 2 | 2 | 8 | [44] |  |  | [45,46] |
| *CG6125* | *SLC26A11* | 1.58 | 6 | 0 | 18 | 26 | [47] |  |  | [48] |
| *Spn42Db* | *SERPINI1* | 3.66 | 6 | 0 | 6 | 5 | [49] |  |  | [50] |
| *PGRP-LD* | *PGLYRP1* | 1.60 | 5 | 2 | 6 | 8 | [51] |  |  | [52] |
| *Cyp6a14* | *CYP3A4* | 2.03 | 5 | 0 | 11 | 13 | [33,34] |  |  | [35] |
| *Cyp6a20* | *CYP3A4* | 1.63 | 5 | 0 | 11 | 13 | [33,34] |  |  | [35] |

### References:

1. Chen WX, Liu B, Zhou L, Xiong X, Fu J, Huang ZF, et al. De novo mutations within metabolism networks of amino acid/protein/energy in Chinese autistic children with intellectual disability. Hum Genomics. 2022;16. doi:10.1186/s40246-022-00427-7

2. Pazhouhandeh M, Sahraian MA, Siadat SD, Fateh A, Vaziri F, Tabrizi F, et al. A systems medicine approach reveals disordered immune system and lipid metabolism in multiple sclerosis patients. Clin Exp Immunol. 2018;192. doi:10.1111/cei.13087

3. Verkhratsky A, Zorec R, Parpura V. Stratification of astrocytes in healthy and diseased brain. Brain Pathol. 2017;27. doi:10.1111/bpa.12537

4. Drokhlyansky E, Smillie CS, Van Wittenberghe N, Ericsson M, Griffin GK, Eraslan G, et al. The Human and Mouse Enteric Nervous System at Single-Cell Resolution. Cell. 2020;182. doi:10.1016/j.cell.2020.08.003

5. Hu J, He Y, Liao K, Yang Q, Xu Y, Cao G, et al. Identification of inflammatory factor-related genes associated with the prognosis and immune cell infiltration in colorectal cancer patients. Genes Dis. 2022. doi:10.1016/j.gendis.2022.07.015

6. Martin J, Cooper M, Hamshere ML, Pocklington A, Scherer SW, Kent L, et al. Biological overlap of attention-deficit/hyperactivity disorder and autism spectrum disorder: Evidence from copy number variants. J Am Acad Child Adolesc Psychiatry. 2014;53. doi:10.1016/j.jaac.2014.03.004

7. Jasani S, Tartaglia G, Yeung PL, Wei Lu C. A Review of the Placenta and Trophoblast Induced Pluripotent Stem Cells in Autism Spectrum Disorder Research. J Stem Cell Res Ther. 2018;08. doi:10.4172/2157-7633.1000413

8. Constantinescu-Bercu A, Grassi L, Frontini M, Salles-Crawley II, Woollard KJ, Crawley JTB. Activated αiibβ3 on platelets mediates flow-dependent netosis via slc44a2. Elife. 2020;9. doi:10.7554/eLife.53353

9. Lévy J, Grotto S, Mignot C, Maruani A, Delahaye-Duriez A, Benzacken B, et al. NR4A2 haploinsufficiency is associated with intellectual disability and autism spectrum disorder. Clin Genet. 2018;94. doi:10.1111/cge.13383

10. Han YF, Cao GW. Role of nuclear receptor NR4A2 in gastrointestinal inflammation and cancers. World J Gastroenterol. 2012;18. doi:10.3748/wjg.v18.i47.6865

11. Zhang W, Cao X, Zhong X, Wu H, Feng M, Gwack Y, et al. Steroid nuclear receptor coactivator 2 controls immune tolerance by promoting induced T reg differentiation via up-regulating Nr4a2. Sci Adv. 2022.

12. Garg P, Sharp AJ. Screening for rare epigenetic variations in autism and schizophrenia. Hum Mutat. 2019;40. doi:10.1002/humu.23740

13. Zhao X, Hu J, Li J, Gu L, Chen J, Othmane B, et al. THEM6: A Novel Molecular Biomarker Predicts Tumor Microenvironment, Molecular Subtype, and Prognosis in Bladder Cancer. Dis Markers. 2022;2022. doi:10.1155/2022/7147279

14. Wu Y, Cao H, Baranova A, Huang H, Li S, Cai L, et al. Multi-trait analysis for genome-wide association study of five psychiatric disorders. Transl Psychiatry. 2020;10. doi:10.1038/s41398-020-00902-6

15. Rossignol DA, Frye RE. Mitochondrial dysfunction in autism spectrum disorders: A systematic review and meta-analysis. Molecular Psychiatry. 2012. doi:10.1038/mp.2010.136

16. East IJ, Washington EA, Brindley PJ, Monroy GF, Scott-Young N. Nematospiroides dubius: Passive transfer of protective immunity to mice with monoclonal antibodies. Exp Parasitol. 1988;66. doi:10.1016/0014-4894(88)90044-6

17. Moretto E, Passafaro M, Bassani S. X-Linked ASDs and ID Gene Mutations. Neuronal and Synaptic Dysfunction in Autism Spectrum Disorder and Intellectual Disability. 2016. doi:10.1016/B978-0-12-800109-7.00009-1

18. Sardaar S, Qi B, Dionne-Laporte A, Rouleau GA, Rabbany R, Trakadis YJ. Machine learning analysis of exome trios to contrast the genomic architecture of autism and schizophrenia. BMC Psychiatry. 2020;20. doi:10.1186/s12888-020-02503-5

19. Kępka A, Ochocińska A, Chojnowska S, Borzym-Kluczyk M, Skorupa E, Knaś M, et al. Potential role of l-carnitine in autism spectrum disorder. Journal of Clinical Medicine. 2021. doi:10.3390/jcm10061202

20. Lee H, Kim HK, Kwon JT, Park S, Park HJ, Kim SK, et al. BBOX1 is down-regulated in maternal immune-activated mice and implicated in genetic susceptibility to human schizophrenia. Psychiatry Res. 2018;259. doi:10.1016/j.psychres.2017.10.018

21. Valiente-Pallejà A, Torrell H, Muntané G, Cortés MJ, Martínez-Leal R, Abasolo N, et al. Genetic and clinical evidence of mitochondrial dysfunction in autism spectrum disorder and intellectual disability. Hum Mol Genet. 2018;27. doi:10.1093/hmg/ddy009

22. Slavotinek A, van Hagen JM, Kalsner L, Pai S, Davis-Keppen L, Ohden L, et al. Jumonji domain containing 1C (JMJD1C) sequence variants in seven patients with autism spectrum disorder, intellectual disability and seizures. Eur J Med Genet. 2020;63. doi:10.1016/j.ejmg.2020.103850

23. Yao Y, Uddin MN, Manley K, Lawrence DA. Improvements of autism-like behaviors but limited effects on immune cell metabolism after mitochondrial replacement in BTBR T+ Itpr3tf/J mice. J Neuroimmunol. 2022;368. doi:10.1016/j.jneuroim.2022.577893

24. Yingjun X, Haiming Y, Mingbang W, Liangying Z, Jiaxiu Z, Bing S, et al. Copy number variations independently induce autism spectrum disorder. Biosci Rep. 2017;37. doi:10.1042/BSR20160570

25. Xia S, Wu J, Zhou W, Zhang M, Zhao K, Liu J, et al. SLC7A2 deficiency promotes hepatocellular carcinoma progression by enhancing recruitment of myeloid-derived suppressors cells. Cell Death Dis. 2021;12. doi:10.1038/s41419-021-03853-y

26. Van Bon BWM, Coe BP, Bernier R, Green C, Gerdts J, Witherspoon K, et al. Disruptive de novo mutations of DYRK1A lead to a syndromic form of autism and ID. Mol Psychiatry. 2016;21. doi:10.1038/mp.2015.5

27. Hoffman AE, Zheng T, Stevens RG, Ba Y, Zhang Y, Leaderer D, et al. Clock-cancer connection in non-Hodgkin’s lymphoma: A genetic association study and pathway analysis of the Circadian gene Cryptochrome 2. Cancer Res. 2009;69. doi:10.1158/0008-5472.CAN-08-4572

28. Hefang Xiao, Yonghui Dong, Likang Xiao, Xiaming Liang, Jia Zheng. Identification of key gene contributingto vitiligo by immune infiltration.

29. Dean DD, Agarwal S, Muthuswamy S, Asim A. Brain exosomes as minuscule information hub for Autism Spectrum Disorder. Expert Review of Molecular Diagnostics. 2021. doi:10.1080/14737159.2021.2000395

30. Chettimada S, Lorenz DR, Misra V, Dillon ST, Reeves RK, Manickam C, et al. Exosome markers associated with immune activation and oxidative stress in HIV patients on antiretroviral therapy. Sci Rep. 2018;8. doi:10.1038/s41598-018-25515-4

31. Im Y, Yoo H, Ko RE, Lee JY, Park J, Jeon K. Exosomal CD63 in critically ill patients with sepsis. Sci Rep. 2021;11. doi:10.1038/s41598-021-99777-w

32. Celestino-Soper PBS, Shaw CA, Sanders SJ, Li J, Murtha MT, Gulhan Ercan-Sencicek A, et al. Use of array CGH to detect exonic copy number variants throughout the genome in autism families detects a novel deletion in TMLHE. Hum Mol Genet. 2011;20. doi:10.1093/hmg/ddr363

33. LS C. Cariprazine in Autism Spectrum Disorder and Intellectual Disability Disorder. Glob J Intellect Dev Disabil. 2019;6. doi:10.19080/gjidd.2019.06.555694

34. Erickson CA, Stigler KA, Posey DJ, McDougle CJ. Aripiprazole in autism spectrum disorders and fragile X syndrome. Neurotherapeutics. 2010;7. doi:10.1016/j.nurt.2010.04.001

35. Hayney MS, Muller D. Effect of Influenza Immunization on CYP3A4 Activity in Vivo. J Clin Pharmacol. 2003;43. doi:10.1177/0091270003260330

36. Lin M, Zhao D, Hrabovsky A, Pedrosa E, Zheng D, Lachman HM. Heat shock alters the expression of schizophrenia and autism candidate genes in an induced pluripotent stem cell model of the human telencephalon. PLoS One. 2014;9. doi:10.1371/journal.pone.0094968

37. Arac A, Brownell SE, Rothbard JB, Chen C, Ko RM, Pereira MP, et al. Systemic augmentation of αB-crystallin provides therapeutic benefit twelve hours post-stroke onset via immune modulation. Proc Natl Acad Sci U S A. 2011;108: 13287–13292. doi:10.1073/pnas.1107368108

38. Lim EMF, Hoghooghi V, Hagen KM, Kapoor K, Frederick A, Finlay TM, et al. Presence and activation of pro-inflammatory macrophages are associated with CRYAB expression in vitro and after peripheral nerve injury. J Neuroinflammation. 2021;18. doi:10.1186/s12974-021-02108-z

39. Hu VW, Nguyen AT, Kim KS, Steinberg ME, Sarachana T, Scully MA, et al. Gene expression profiling of lymphoblasts from autistic and nonaffected sib pairs: Altered pathways in neuronal development and steroid biosynthesis. PLoS One. 2009;4. doi:10.1371/journal.pone.0005775

40. AlAyadhi LY, Hashmi JA, Iqbal M, Albalawi AM, Samman MI, Elamin NE, et al. High-resolution SNP genotyping platform identified recurrent and novel CNVs in autism multiplex families. Neuroscience. 2016;339. doi:10.1016/j.neuroscience.2016.10.030

41. Rees E, Kendall K, Pardiñas AF, Legge SE, Pocklington A, Escott-Price V, et al. Analysis of intellectual disability copy number variants for association with schizophrenia. JAMA Psychiatry. 2016;73. doi:10.1001/jamapsychiatry.2016.1831

42. Osborne N, Leahy C, Lee YK, Rote P, Song BJ, Hardwick JP. CYP4V2 fatty acid omega hydroxylase, a druggable target for the treatment of metabolic associated fatty liver disease (MAFLD). Biochemical Pharmacology. 2022. doi:10.1016/j.bcp.2021.114841

43. Reumer A, Bogaerts A, Van Loy T, Husson SJ, Temmerman L, Choi C, et al. Unraveling the protective effect of a Drosophila phosphatidylethanolamine-binding protein upon bacterial infection by means of proteomics. Dev Comp Immunol. 2009;33. doi:10.1016/j.dci.2009.06.010

44. Swanberg SE, Nagarajan RP, Peddada S, Yasui DH, Lasalle JM. Reciprocal co-regulation of EGR2 and MECP2 is disrupted in Rett syndrome and autism. Hum Mol Genet. 2009;18. doi:10.1093/hmg/ddn380

45. Okamura T, Fujio K, Sumitomo S, Yamamoto K. Roles of LAG3 and EGR2 in regulatory T cells. Annals of the Rheumatic Diseases. 2012. doi:10.1136/annrheumdis-2011-200588

46. Miao T, Symonds ALJ, Singh R, Symonds JD, Ogbe A, Omodho B, et al. Egr2 and 3 control adaptive immune responses by temporally uncoupling expansion from T cell differentiation. J Exp Med. 2017;214. doi:10.1084/jem.20160553

47. Nguyen LS, Kim HG, Rosenfeld JA, Shen Y, Gusella JF, Lacassie Y, et al. Contribution of copy number variants involving nonsense-mediated mRNA decay pathway genes to neuro-developmental disorders. Hum Mol Genet. 2013;22. doi:10.1093/hmg/ddt035

48. Zhang F, Zhong W, Li H, Huang K, Yu M, Liu Y. TP53 Mutational Status-Based Genomic Signature for Prognosis and Predicting Therapeutic Response in Pancreatic Cancer. Front Cell Dev Biol. 2021;9. doi:10.3389/fcell.2021.665265

49. Fillman SG, Sinclair D, Fung SJ, Webster MJ, Shannon Weickert C. Markers of inflammation and stress distinguish subsets of individuals with schizophrenia and bipolar disorder. Transl Psychiatry. 2014;4. doi:10.1038/tp.2014.8

50. Rajaraman P, Brenner A V., Butler MA, Wang SS, Pfeiffer RM, Ruder AM, et al. Common variation in genes related to innate immunity and risk of adult glioma. Cancer Epidemiol Biomarkers Prev. 2009;18. doi:10.1158/1055-9965.EPI-08-1041

51. Gonzalez-Santana A, Diaz Heijtz R. Bacterial Peptidoglycans from Microbiota in Neurodevelopment and Behavior. Trends in Molecular Medicine. 2020. doi:10.1016/j.molmed.2020.05.003

52. Dziarski R, Gupta D. Mammalian peptidoglycan recognition proteins (PGRPs) in innate immunity. Innate Immunity. 2010. pp. 168–174. doi:10.1177/1753425910366059
