# Supplementary material for: The ortholog of human ssDNA-binding protein SSBP3 influences neurodevelopment and autism-like behaviors in Drosophila melanogaster
Source: PLoS Biol. 2023 Jul 24;21(7):e3002210. doi: 10.1371/journal.pbio.3002210 (PMC10399856; doi:10.1371/journal.pbio.3002210)
Supplement: S1 Raw Image — Lanes labeled with X were not depicted in the blot in Fig 6A but have been used to perform quantification depicted in the bar graphs. (PDF) [file pbio.3002210.s017.pdf]

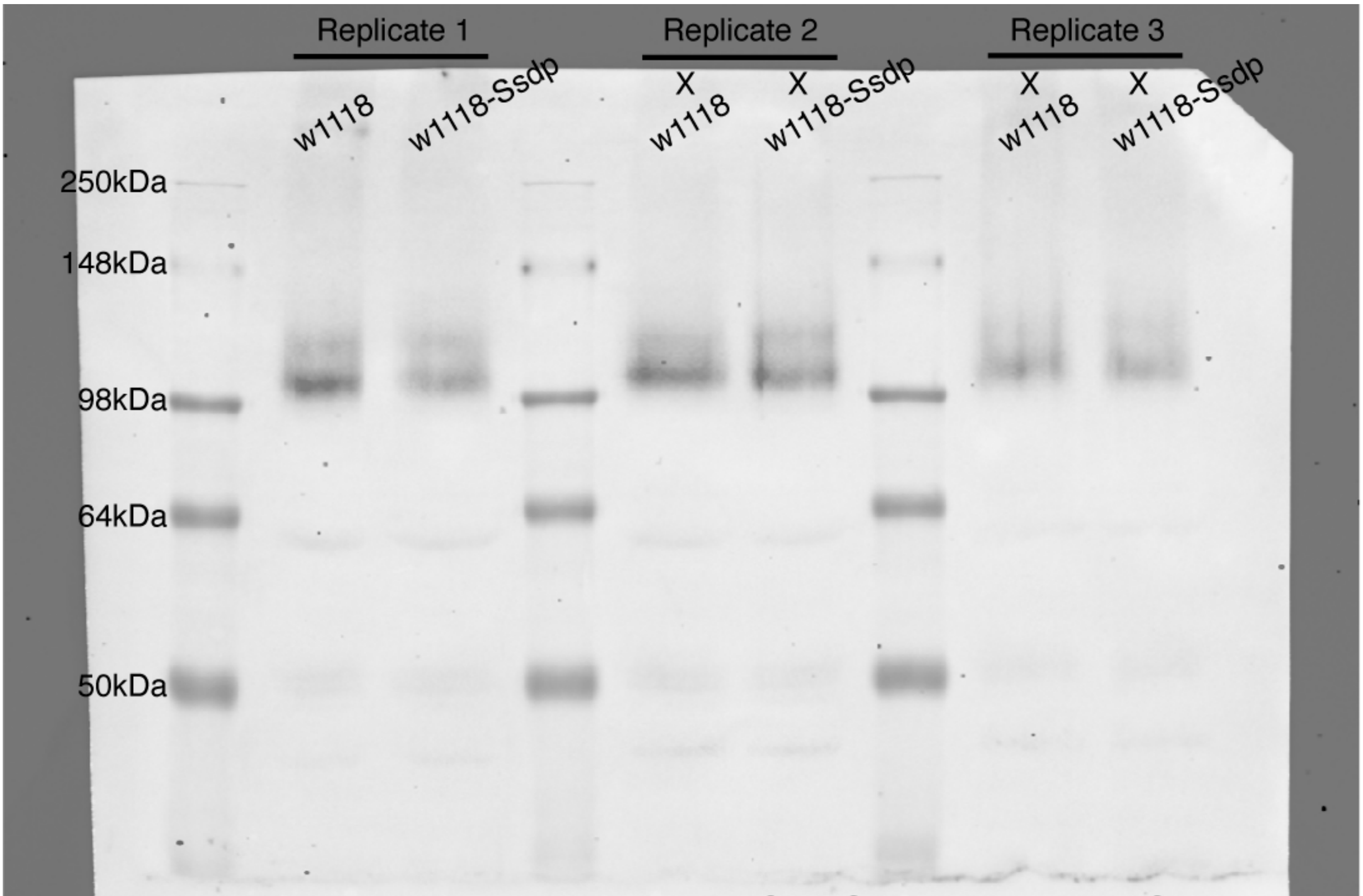

Anti-Armadillo blot was developed using SuperSignal West Pico PLUS Chemiluminescent Substrate (ThermoScientific 34578) and imaged in Bio-Rad ChemiDoc MP Imaging Blot System. Blot used to generated panel 6A.

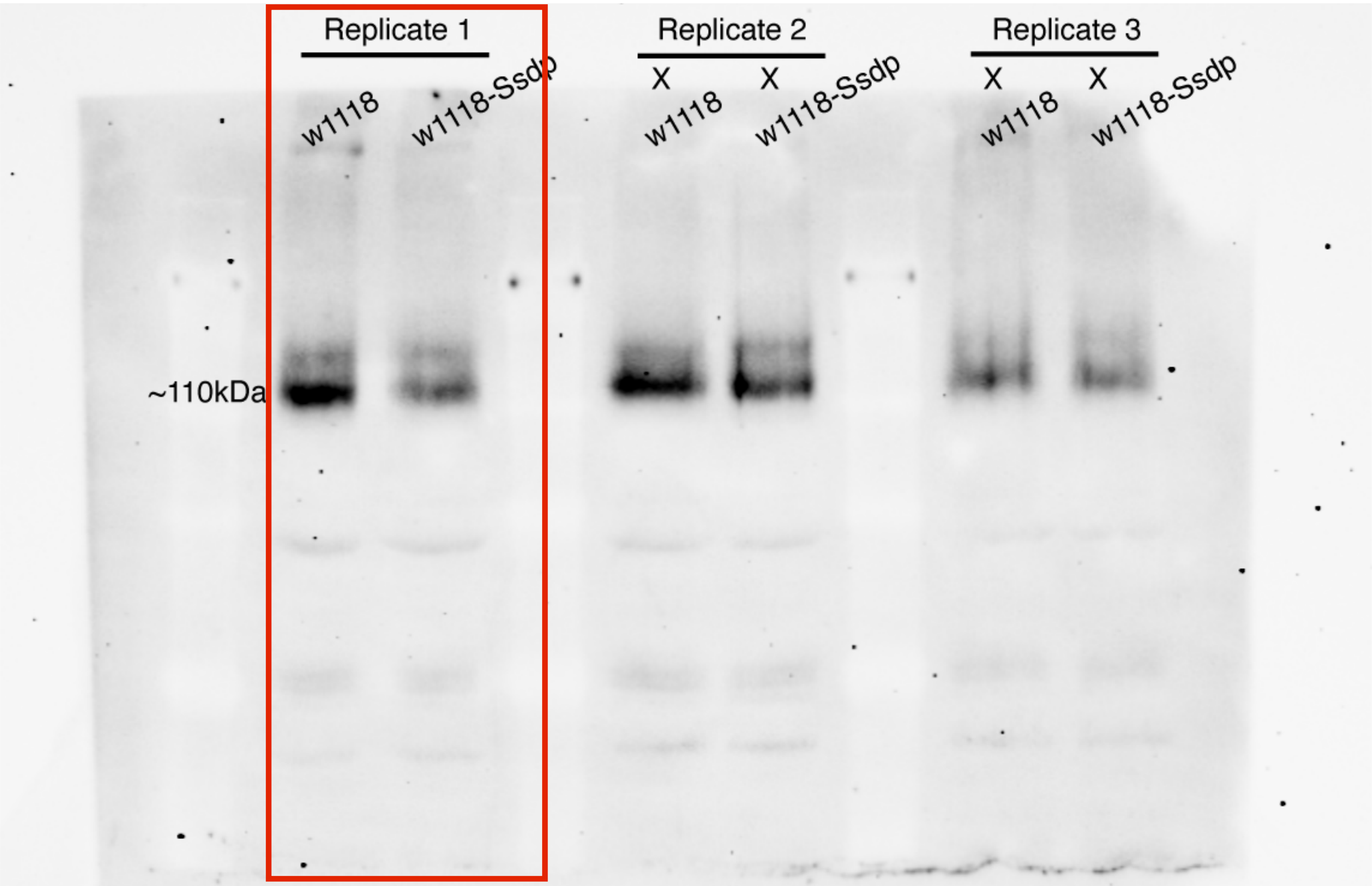

- Anti-Armadillo blot was developed using SuperSignal West Pico PLUS Chemiluminescent Substrate (ThermoScientific 34578) and imaged in Bio-Rad ChemiDoc MP Imaging Blot System. Blot used to generated panel 6A.

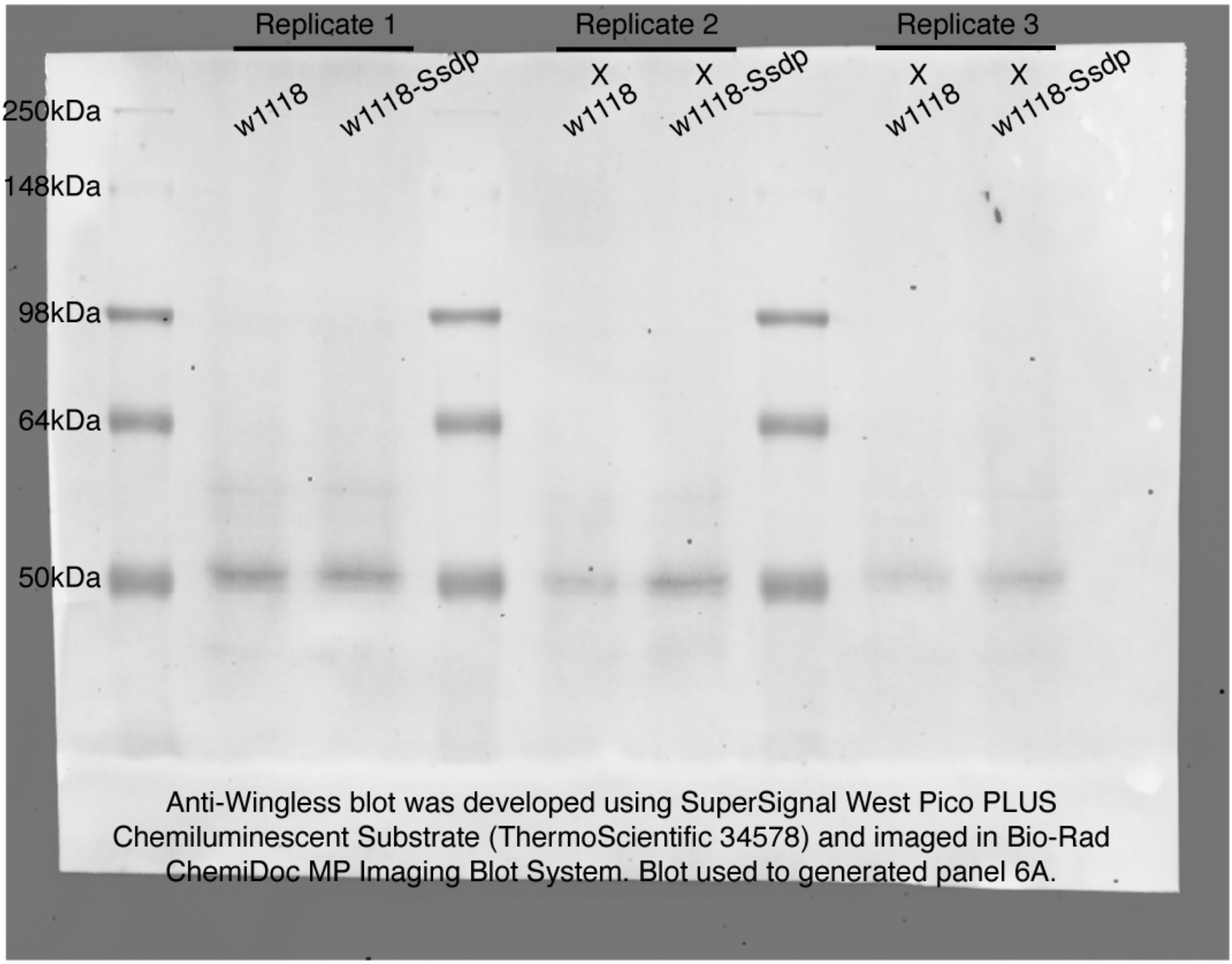

Anti-Wingless blot was developed using SuperSignal West Pico PLUS Chemiluminescent Substrate (ThermoScientific 34578) and imaged in Bio-Rad ChemiDoc MP Imaging Blot System. Blot used to generated panel 6A.

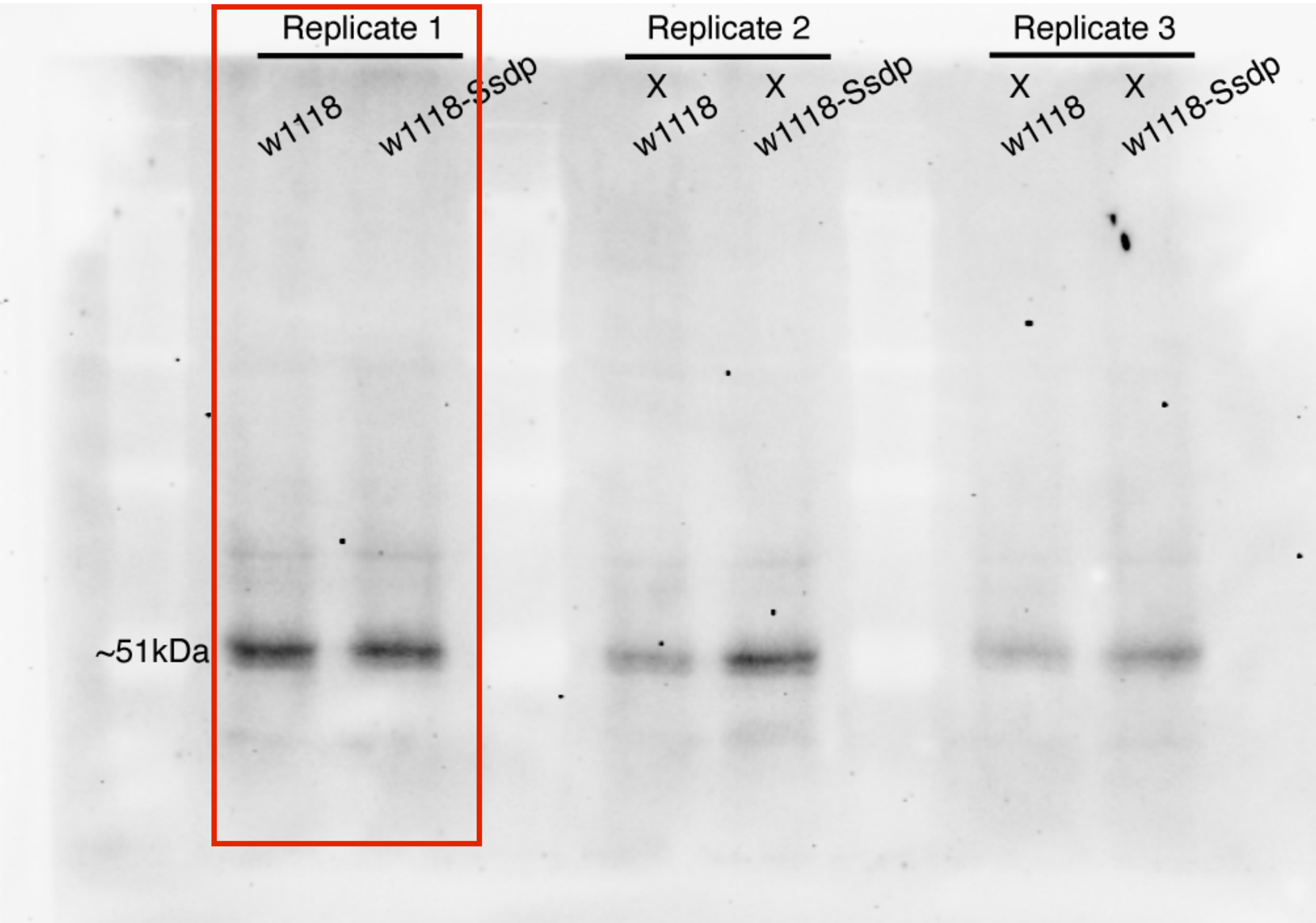

Anti-Wingless blot was developed using SuperSignal West Pico PLUS Chemiluminescent Substrate (ThermoScientific 34578) and imaged in Bio-Rad ChemiDoc MP Imaging Blot System. Blot used to generated panel 6A.

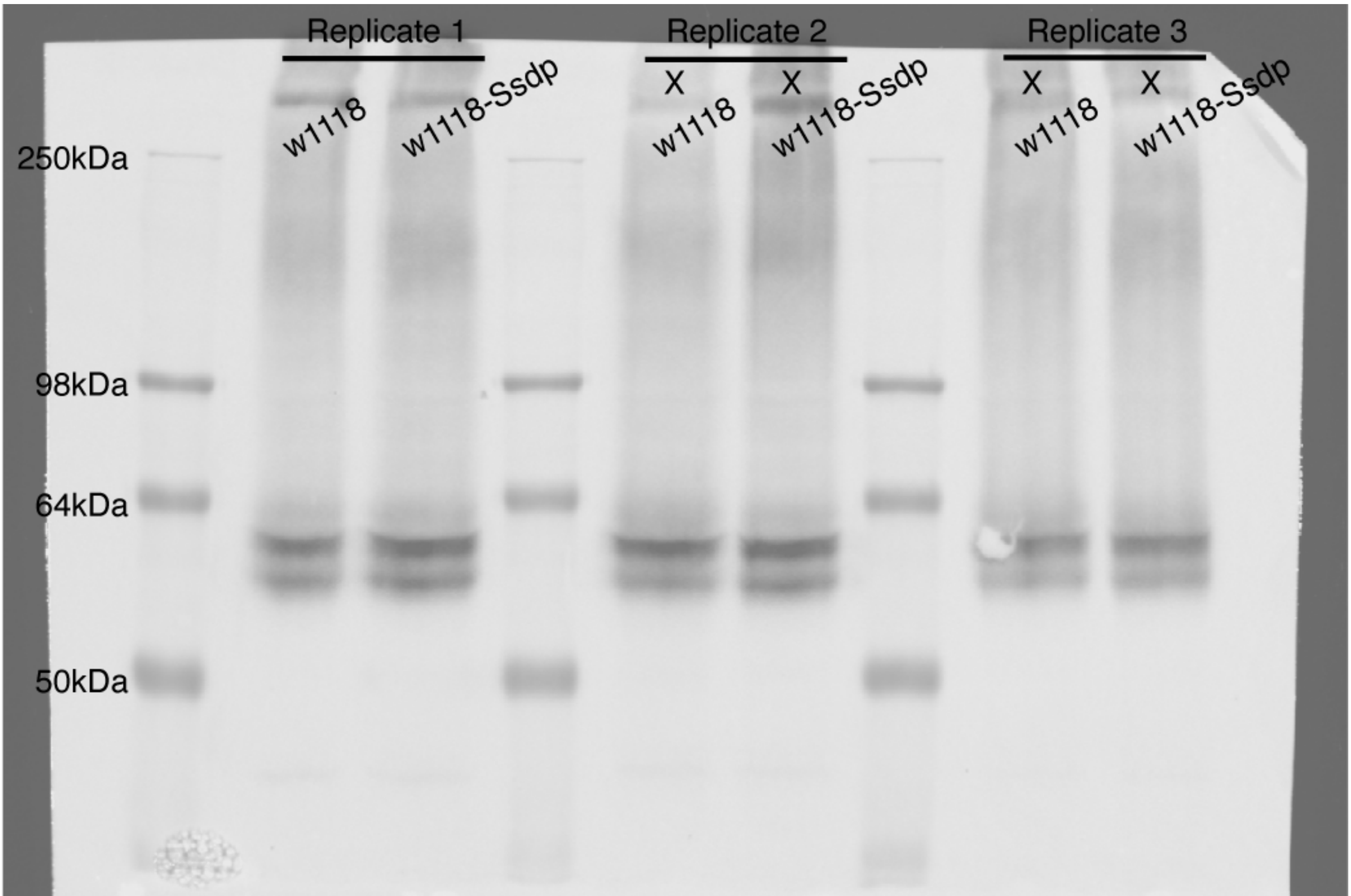

Anti-Tubulin blot was developed using SuperSignal West Pico PLUS Chemiluminescent Substrate (ThermoScientific 34578) and imaged in Bio-Rad ChemiDoc MP Imaging Blot System. Blot used to generated panel 6A.

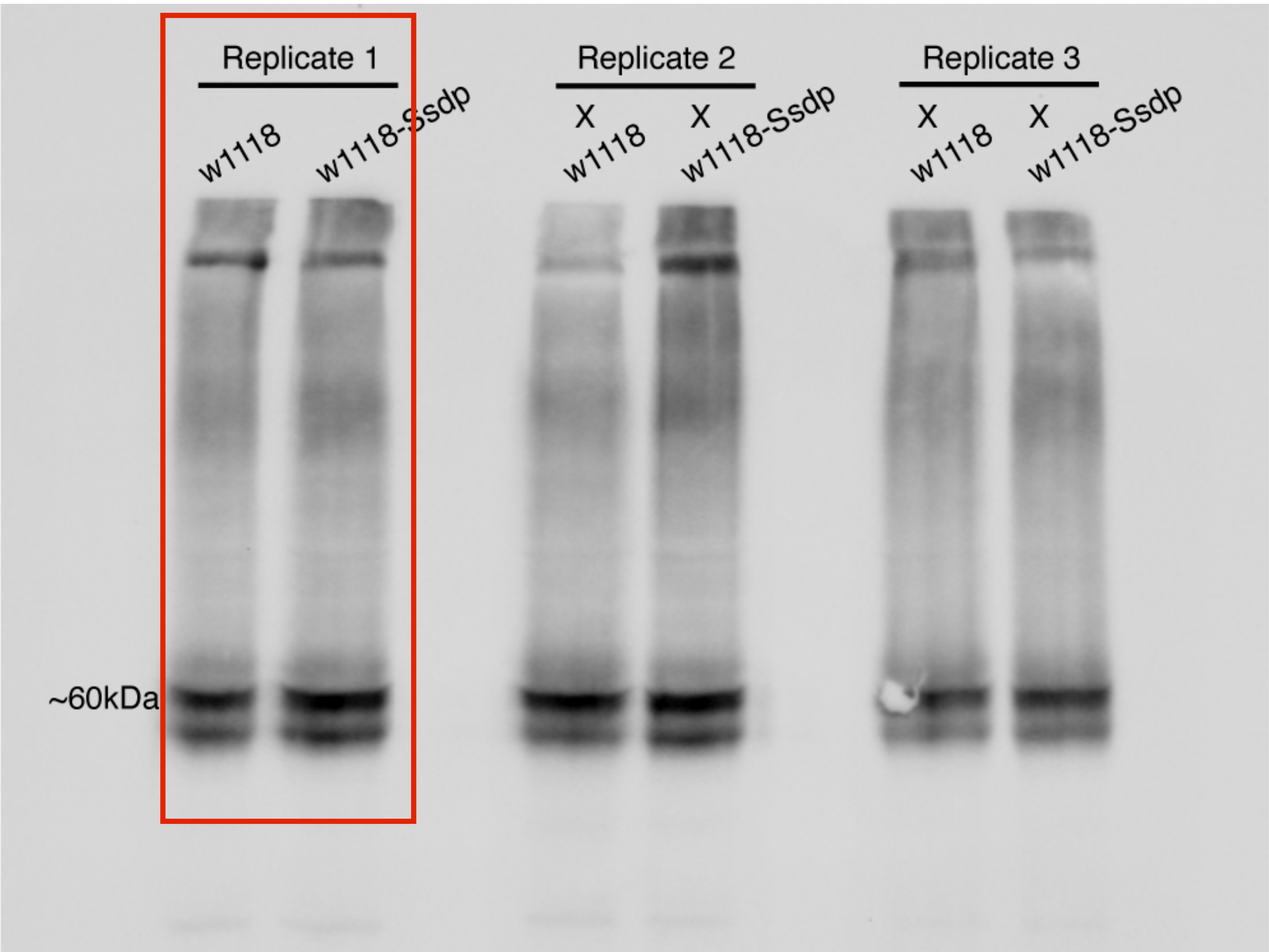

Anti-Tubulin blot was developed using SuperSignal West Pico PLUS Chemiluminescent Substrate (ThermoScientific 34578) and imaged in Bio-Rad ChemiDoc MP Imaging Blot System. Blot used to generated panel 6A.
