# Supplementary material for: The ortholog of human ssDNA-binding protein SSBP3 influences neurodevelopment and autism-like behaviors in Drosophila melanogaster
Source: PLoS Biol. 2023 Jul 24;21(7):e3002210. doi: 10.1371/journal.pbio.3002210 (PMC10399856; doi:10.1371/journal.pbio.3002210)
Supplement: Attachment — Submitted filename: Response to Editorai-requests and Reviewers.pdf [file pbio.3002210.s037.pdf]

Response to Editor:

Please find below our response to all editorial requests:

1. TITLE: After some discussion within the team, we think the title should be modified slightly to make clearer the link between ssdp and human SSB3. If you agree, we suggest you change the title to "The ortholog of human single-stranded DNA-binding protein SSBP3 influences neurodevelopment and autism-like behaviors in Drosophila melanogaster"

>> We are happy to keep the suggested title "The ortholog of human single-stranded DNA-binding protein SSBP3 influences neurodevelopment and autism-like behaviors in Drosophila melanogaster."

2. FINANCIAL DISCLOSURES: I noticed in your financial disclosure that you report having received no specific funding for this work. Even if you did not have a grant specifically written to fund this project, we would require that you acknowledge the sources of money that funded this work, including salaries, etc. You should also acknowledge institutional funding or private grants as well, if relevant.

>> We have mentioned grants in the acknowledgement section, which have partly helped in execution of this manuscript. Line 1009-1010.

3. DATA: Thank you for depositing your RNA seq datasets on the GEO database - can you please provide me with a reviewer token so that I can access this data and make sure it complies with our data policies?

>> To review GEO accession GSE220311: Go to https://eur05.safelinks.protection.outlook.com/?url=https%3A%2F%2Fwww.ncbi.nlm.nih.gov%2Fgeo%2Fquery%2Facc.cgi%3Facc%3DGSE220311&data=05%7C01%7C%7C5476d17f48ad4ee3ef8f08db635bd987%7C0edca4720b7146e696c70a68c10dcb96%7C0%7C0%7C638213018369793069%7CUnknown%7CTWFpbGZsb3d8eyJWIjoiMC4wLjAwMDAiLCJQIjoiV2luMzIiLCJBTiI6Ik1haWwiLCJXVCI6Mn0%3D%7C3000%7C%7C%7C&sdata=eTTq%2FuQ6zt1AqaoWEP0%2FTKGk0CPIQ27RJZCdfHSxuHM%3D&reserved=0

Enter token klajeuoivjwbtiv into the box

4. DATA: In addition to access to the RNA seq data, we ask that you provide the underlying data for each of your figures. You may be aware of the PLOS Data Policy, which requires that all data be made available without restriction:
Note that we do not require all raw data. Rather, we ask that all individual quantitative observations that underlie the data summarized in the figures and results of your paper be made available in one of the following forms:

a Supplementary files (e.g., excel). Please ensure that all data files are uploaded as 'Supporting Information' and are invariably referred to (in the manuscript, figure legends, and the Description field when uploading your files) using the following format verbatim: S1 Data, S2 Data, etc. Multiple panels of a single or even several figures can be included as multiple sheets in one excel file that is saved using exactly the following convention: S1_Data.xlsx (using an underscore).

>>>Regardless of the method selected, please ensure that you provide the individual numerical values that underlie the summary data displayed in the following figure panels as they are essential for readers to assess your analysis and to reproduce it:
Fig 2E; Fig 4B,D,F,H; Fig 5A-F; Fig 6A-D; Fig 7I-J,L; Fig 8B-C; Fig 9A-E,G,I; Fig 10B-C,E,G;
Fig S2A; S7; S8; S9; S10; S11

NOTE: the numerical data provided should include all replicates AND the way in which the plotted mean and errors were derived (it should not present only the mean/average values). Please also ensure that figure legends in your manuscript include information on where the underlying data can be found, and ensure your supplemental data file/s has a legend.

>> We have provided raw data as a S1_Data.xlsx and mentioned the availability of raw data in each figure legend as well.

5) BLOT AND GEL REPORTING:
Please provide the original, uncropped blots accompanying Fig 6A.

>>We have included here uncropped blots related to Fig 6A.

6) DATA NOT SHOWN: Please note that per journal policy, we do not allow the mention of "data not shown", "personal communication", "manuscript in preparation" or other references to data that is not publicly available or contained within this manuscript. Please either remove mention of these data or provide figures presenting the results and the data underlying the figure(s). I noticed one instance of this on page 13 “however downregulated genes were not enriched for any brain function related biological process GO terms (not shown)"

>> We have removed mentioning of “data not shown” in the revised manuscript.

Reviewer #1: The author answered the questions I raised, supported by additional figures or clarifications. These answers are compelling and I think the paper is now mature for publication.

>> We are thankful to the reviewers for their thorough review and constructive comments, which have undoubtedly enhanced the quality and readiness of this manuscript. We look forward to the opportunity to share our research findings with the broader scientific community through publication.

Reviewer #2: The revised version of the manuscript addressed most if not all the points that have been raised by the reviewers and overall, the neurodevelopmental analysis has been substantially improved and strengthen by the new data and analysis. I have appreciated the way the authors answered my comments and the experimental strategies undertaken to solve the critical points and to improve the rigor of the study. I still have some minor comments regarding the revised version that I would like to see addressed:

>> We are pleased to hear that the reviewer agrees that most of their points have been addressed, we appreciate the recognition of our efforts to address the comments raised by the reviewers and enhance the rigor of the study through experimental strategies. We are committed to further refining the manuscript based on your remaining minor comments to ensure the highest quality of the final

publication. Your continued input and suggestions are invaluable in shaping the manuscript and we appreciate your thorough and thoughtful review. We have carefully considered your comments and below provide detailed responses and appropriate revisions in the this version of the manuscript.

1. I do appreciate the new volumetric analysis of the brains that takes into account all the stacks, and the new results are indeed more accurate. However, as I have highlighted in the previous rounds of comments regarding the Elav-immunostaining, I do not think the analysis performed as it is, gives any insight into the effect of Ssdp on brain volume. If Ssdp levels alteration affects at the same time neuronal number and size, no difference in volume would emerge and the defect would not be detected. This might be the case. In fact, in line 205-207 and 243-244, the authors use anti-Elav immunostainings to quantify brain volume without seeing any difference. I think that, if the authors would like to use Elav as a marker they should perform a quantification of cell numbers, as they did for Repo.

>> Thank you, for your valuable feedback and suggestions. We appreciate your recognition of the improved volumetric analysis and the accuracy of our nc82 data in assessing brain volume, which matches with anti-Dlg based brain volume analysis. We understand your concern regarding the brain volume analysis using Elav-positive cells and the potential challenges associated with their small numbers and clustered nature. Considering these factors and the availability of reliable nc82 data in the manuscript, as reviewers suggested and agreed that it provides an accurate measure of brain volume, we decided to remove the Elav-based approach for quantifying brain volume from the revised manuscript.

2. I wonder why in the analysis of brain volume of Ssdp knockdown flies, the authors did not perform the anti-Bruchpilot analysis as in the overexpression flies (see lines 239-240 compared to 203-205).

>> We thank the Reviewer, for their comment regarding the differences in approach for brain volume analysis in Ssdp overexpressing and knockdown flies. We appreciate your observation and would like to provide clarification on this matter. In our study, we utilized anti-nc82 (also known as anti-Bruchpilot) immunostaining to analyze brain volume in the Ssdp overexpression flies. This antibody specifically labels presynaptic active zones and serves as a marker for synaptic organization. We demonstrated the comparability of the volume analysis using anti-nc82 and anti-Dlg antibodies in the Ssdp overexpression flies, as shown in Figure XX. However, in the analysis of brain volume in the Ssdp knockdown flies, we solely relied on the anti-Dlg immunostaining approach. This was a decision based on the validation results from the Ssdp overexpression flies, where we did not observe any significant differences between the anti-nc82 and anti-Dlg analyses. Therefore, we deemed it appropriate to utilize the anti-Dlg immunostaining method for consistency in our quantification of brain volume.

3. Lines 282-284: I think it would be useful to cite again the figure panel the authors are referring to, after describing the differences in the number of stripes, maybe at the end of line 284.
>> We appreciate reviewer's attention to detail. In the revised manuscript, we have made the necessary revisions to address this oversight.

4. In figure S7 the authors perform some RT-PCR on brains to confirm the data on mitochondrial fission/fusion genes obtained from heads extract (lines 1326-1327). Interestingly, Marf1 levels are not upregulated in the brains, while they are in the head extracts (Fig 8C) that also include eyes and cuticles. Does this suggest that the upregulation of Marf1 is dependent on these tissues? I would like the authors to comment on this.
>> We appreciate the reviewer's insightful comment and the opportunity to address their concern. The

differential expression pattern of Marf1 between brain and head extracts, as observed in our study, can be attributed to several factors. First, it is important to note that the brain and head extracts contain different tissues, including the eyes and cuticle, which can contribute to the overall gene expression profile in the head extracts. In contrast, the brain-specific expression of Ssdp and its localized expression in fewer neurons might limit its influence on Marf1 expression in the whole brain. This could potentially dilute any significant changes in Marf1 expression that may be present in the specific neuronal subpopulations affected by Ssdp. While we did observe a trend of upregulation in Marf1 expression in the brain (1.2-fold change), this increase did not reach statistical significance. We have discussed this limitation in the manuscript, emphasizing that the lack of statistical significance could be due to the dilution of information from the whole brain, where Ssdp may not be expressed or have a significant influence on Marf1 expression. Importantly, the trend of upregulation in Marf1 expression supports the overall findings of increased Marf1 expression in the context of Ssdp dysregulation.

5.In figure 9G the RNAi control (blue dots) seems to display an interaction time much closer to that of the Elav-RNAi flies, rather than to that of the other control (grey dots, Elav alone). Can the authors comment on this? In most of the other experiments it behaves very similar to the other control. Is the statistics calculated by pooling the two controls together and comparing them to the Elav-RNAi strain? What is the genotype of the two controls, are they outcrossed to a control strain? (i.e. Elav/+ and RNAi/+)
>>

6. In figure S9 the authors show a partial rescue of behavioral defects upon knocking down Ssdp expression in Ssdp-overexpressing cells. Is there any statistical analysis performed between the Ssdp-Gal4 and Ssdp-Gal4>Ssdp-RNAi?
>>

7. In the discussion, lines 640-641 the authors state that "Ssdp may plays an inhibitory or excitatory role in a tissue context-dependent manner", I think that "excitatory" is not the correct term for Ssdf function, maybe I would refer to "activatory".
>> we agree with the reviewer and have changed the excitatory to activatory in lines 640-641

8. In the discussion, lines 648-650 the authors state "Therefore, our findings suggest that Ssdp may play a critical role in glial cell function during neurodevelopment and maintenance of the nervous system". Since the evidence produced regards the number of glia cells, I think this statement should be tuned down.
>> we agree with reviewer, and we have revised our statement.

9. In line 667-668 the authors state "Reduced Wnt/β-catenin signaling in Ssdp[2082-G4] brains may have resulted in larger brains and reduced gliogenesis". If the meaning of this sentence is that a reduction in Wnt signaling might represent the molecular mechanism underlying larger brain and reduced gliogenesis then the authors need to rephrase the concept
>> We thanks reviewer for their comments and we have revised our statement to reflect reviewers suggestion.
