# Supplementary material for: The ortholog of human ssDNA-binding protein SSBP3 influences neurodevelopment and autism-like behaviors in Drosophila melanogaster
Source: PLoS Biol. 2023 Jul 24;21(7):e3002210. doi: 10.1371/journal.pbio.3002210 (PMC10399856; doi:10.1371/journal.pbio.3002210)
Supplement: Attachment — Submitted filename: Response-reviewers_PlosBio_4-05-2023.docx [file pbio.3002210.s036.docx]

Reviewer #1:

This paper by Safa Salim et al. makes the following conclusions: that the human gene SSBP3 located in the chromosome band 1p32.3, whose microdeletion/duplication is associated with neurodevelopmental defects and autism has its Drosophila melanogaster ortholog called Ssdp. Through a knockdown and overexpression approaches they showed that Ssdp plays a role in the "brain development" and autism-like behaviors in flies. At the cellular and molecular levels, Ssdp regulates gliogenesis, Wnt signaling, and oxidative reductase and mitochondrial function. Understanding the mechanism of neurodevelopmental disorders (NDDs) such as autism caused by genomic dysfunctions including chromosomal rearrangements is important. Aberrations in 1p32.3 have been reported in patients with NDDs, but the link between genes located within the 1p32.3 region and autism spectrum disorders (ASD) remains unclear. However, there are a number of issues that would need to be addressed before a possiblepublication.

>> We appreciate the reviewers' feedback on our study and for providing their constructive feedback. Please find here our comments on the questions raised.

1) The most significant major comment I have is related to the evidence on neuro-developmental defects or disorders in the presented study. The authors claim across the manuscript that Ssdp influences neurodevelopment, but all the data were conducted or collected after the development: from fully mature brains or adult flies. What is the evidence that links Ssdp expression to neurodevelopment?

It is important for the authors to demonstrate that (i) brain structures and function at early developmental stages (e.g. larvae and pupae) are also affected in the condition of constant Ssdp overexpression and knockdown; (ii) and in a conditional experiment to exclusively manipulate Ssdp expressions in the adult brain there are no cellular and behavioral defects. These fairly experiments would significantly improve the rigor of the work.

>>Thank you for your valuable feedback. The primer reason of our claim was based on genetic manipulations which were done throughout development. However, we agree with the reviewers' comments and have made significant updates to our manuscript to address the concerns raised.

1. We have now included data on brain structure changes in larvae, where *Ssdp* expression was manipulated either by overexpression (lines 198-203 and lines 215-218, Fig 4A, G) or knockdown (lines 239-241 and lines 245-247, Fig 5B, E). This provides evidence of the impact of *Ssdp* on neurodevelopment during early stages of brain development, strengthening our claim that *Ssdp* influences neurodevelopment.
2. In addition, we have included behavioral data when *Ssdp* expression was exclusively manipulated in adult brains (lines 539-552, S10 Fig A-D). This conditional experiment strongly suggest that alterations in Ssdp levels throughout development manifest as behavioral and cellular defects in adult flies.
3. Furthermore, we have included data on cellular effects when *Ssdp* levels were manipulated only in adult brains (lines 552-555, S10 Fig E). We found no cellular effects when *Ssdp* levels were manipulated only in adult brains, complementing our findings on neurodevelopmental defects.

We believe that the inclusion of these additional datasets significantly enhances the rigor of our study and strengthens our conclusions on the developmental role of Ssdp and its regulation of autism-like behaviors in Drosophila.

2) Fig. 2C: What do Ssdp-positive neurons look like in Ssdp[2082-G4] flies? Are their structural components (axons, dendrites, soma) affected? These experiments would provide key insight at the cellular level into the neuro-developmental nature of this study.

>> We appreciate the reviewers' comments and their suggestion to provide more insights at the cellular level into the neurodevelopmental nature of our study. In response to this feedback, we have now included nc82 images and data on synaptic density in Fig 4E, F and lines 210-215. We have also attempted to label axons and dendrites using UAS-DenMark;UAS-syt, but unfortunately, this cross resulted in developmental lethality.

These additional experiments and data on synaptic density provide valuable information on the structural components of Ssdp-positive neurons in Ssdp[2082-G4] flies. We believe that these findings further support our conclusions on the role of Ssdp in neurodevelopment and its potential impact on synaptic function in Drosophila. We are grateful for the reviewers' feedback, and have made efforts to address their concerns and enhance the rigor of our study.

Thank you for considering our response, and we hope that our revisions adequately address the questions raised by the reviewers.

3) Page 4 and Fig. 2E: I am curious about Ssdp upregulation in Ssdp[2082-G4]? Does an expression of UAS-SsdpRNAi driven by Ssdp[2082-G4] lead to a normalization of Ssdp levels?

>> We agree with the reviewers' curiosity regarding *Ssdp* upregulation in Ssdp[2082-G4] flies and the potential normalization of *Ssdp* levels upon knockdown using UAS-Ssdp-RNAi driven by Ssdp[2082-G4]. In response to this question, we have performed additional experiments and included data on this topic in S9 Fig and presented results in lines 518-538. We observed partial rescue of behavioral defects when *Ssdp* expression was knocked down in Ssdp-overexpressing cells in Ssdp[2082-G4] flies. Specifically, all behaviors except for social interaction were rescued upon knockdown of *Ssdp* in Ssdp-overexpressing cells in Ssdp[2082-G4] flies.

These findings provide important insights into the relationship between *Ssdp* expression levels and behavioral outcomes in our study. They suggest that modulating *Ssdp* expression in Ssdp[2082-G4] flies can partially rescue the observed behavioral defects, further supporting the role of *Ssdp* in regulating autism-like behaviors in Drosophila. We thank the reviewers for their comments, and we have included these additional data to further strengthen our findings.

4) The link between gene dosage and diseases is well-described in the literature. What are the consequences of an overexpression of Ssdp using Ssdp[2082-G4] or Elav-Gal4 on brain development and behaviors?

>> We appreciate the reviewers' comment on the potential consequences of Ssdp overexpression on brain development and behaviors using Ssdp[2082-G4] or Elav-Gal4 driver lines. We agree that investigating the effects of Ssdp overexpression would provide valuable insights into the role of Ssdp in neurodevelopmental processes and behaviors.

However, we regret to inform that currently there are no UAS-Ssdp flies available for conducting such experiments to directly address this question. We acknowledge the importance of exploring the effects of Ssdp overexpression in future studies to fully understand the implications of altered gene dosage on brain development and behaviors. We have included this in the limitation section in the discussion.

5) It is unclear (Pages 6,7 and Figs. 3,4) how the authors explain the much larger neuropil, while the whole-brain morphology labeled by Elav or repo show no changes? Did author try another neuropil marker (e.g. nc82).

>> We thank reviewers for bringing this to our attention on the discrepancy between the observed larger neuropil and the lack of changes in whole-brain morphology labeled by Elav or repo markers. Upon careful consideration, we propose that the larger neuropils observed in our study may be attributed to the increased size of axons and dendrites. To provide additional evidence supporting this hypothesis, we have performed immunohistochemistry (IHC) using the nc82 marker, which specifically labels synaptic neuropils in *Drosophila melanogaster* brains (Fig 4C, D and lines 203-205). Our IHC data using nc82 staining indeed show larger neuropils in Ssdp[2082-G4] flies, providing further support to our interpretation that the observed changes in neuropil size are likely due to altered axon and dendrite morphology.

We appreciate the reviewers' suggestion and have now included the nc82 IHC data in our revised manuscript to further support our findings. and we hope that the additional data and explanation provide a clearer understanding of our results.

6) Fig. 3C-F and Fig. 4D-G: Immunostaining Anti-repo or anti-Elav normally targets thousands of cells stacked on top of each other. How did the authors count Also, how did the authors count the neuron or glia cell bodies in the brains. A more detailed description of the counting method as well as the provision of all the dataset (e.g. Excel, tiff files) supporting this analysis are important to convince the readers.

>> We appreciate the reviewers' comment on the counting method for neuron or glia cell bodies in our study. We have provided a more detailed description of the counting method, in the revised manuscript (lines 827-839). We have performed a re-analysis of the cell counting using a 3D counter to accurately quantify the cell bodies in the brains. We have updated the figures (Fig 3C-F and Fig 4D-G) with the revised cell counts (updated Fig 4B, D, H and Fig 5B, C, D, E, F).

Additionally, we have revised our manuscript to provide a more detailed description of the counting method in the Materials and Methods section, including information on the software and settings used for the 3D cell counting (lines 838-850). We have also provided dataset, including Excel files and TIFF images in our manuscript (S1 file), and we have also provided supplementary video files 1-22 that go through the stacks of representative larval CNS/adult brains labeled by anti-DLG, anti-Elav, anti-nc82, and anti-Repo antibodies. Further, we have included a statement to indicate our willingness to provide any dataset, included in the paper, upon request to ensure transparency and reproducibility of our findings.

7) What is the evidence for the specificity of the RNAi?

In this manuscript the authors employ one RNAi transgene for Ssdp. RNA-interference is often conducted by expressing inverted repeats of cDNA fragments matching a gene of-interest using the Gal4/UAS system, as done in this study. However, this approach is notoriously prone to off-target effects. This is of particular concern when monitoring behavioral phenotypes, which are notoriously sensitive to genetic background.

- Has the SsdpRNAi line used in this manuscript (Fig. 4 and 8) been previously validated? Anyway, to what extent it reduce Ssdp expression? A simple experiment is to conduct a PCR experiment in the presence and absence of SsdpRNAi.

>> We thank reviewers for their comments. Actually, we have provided qPCR data in our manuscript (Fig 5A) to measure the efficiency of Ssdp-RNAi. We have observed more than 50% downregulation of Ssdp expression when RNAi was expressed using Elav-Gal4, indicating a significant reduction in Ssdp levels in the experimental group compared to the control group (lines 227-229).

- Can the authors observe effects on the brain (as in figure 4) as well as behaviors (as in Fig. 8) with additional SsdpRNAi transgenes?

>>We appreciate the reviewers' comment and acknowledge the importance of further validating our findings with additional SsdpRNAi transgenes. We attempted to obtain an additional RNAi line from VDRC, but unfortunately, these flies were not viable, which precluded us from conducting further experiments with them.

Despite this limitation, we believe that our current findings using Ssdp[2082-G4] and Elav-Gal4>Ssdp-RNAi flies provide robust evidence for the effects of Ssdp on brain morphology (as shown in Fig 4 and 5) as well as behaviors (as shown in Fig 8 and S8 Fig). The consistency of the phenotypes observed between Ssdp[2082-G4] and Elav-Gal4>Ssdp-RNAi flies supports the validity of our findings.

However, we acknowledge the limitation of not being able to use additional SsdpRNAi transgenes in our study, and we have included this limitation in the discussion section of our manuscript (lines 740-743).

8) On a related note, knockdown approach raises the question of what is the null phenotype? It may be the same palette of phenotypes but at a greater penetrance, or it may be a completely different phenotype. It is important for the authors to try other knockout tools (mutants) to determine the full LOF phenotype.

>>We appreciate the reviewers' comment and acknowledge the importance of determining the full loss-of-function (LOF) phenotype of *Ssdp*. However, we would like to highlight that homozygous Ssdp[20G4] flies are lethal, which precludes us from using this approach to determine the complete LOF phenotype of *Ssdp*.

9) Figure 8: What is the behavioral consequences of Ssdp knockdown in Ssdp positive cells using Ssdp[2082-G4] driver? is there a rescue of phenotypes?

>> We appreciate the reviewers' comment. As mentioned earlier, We have performed additional experiments to investigate the behavioral consequences of *Ssdp* knockdown specifically in Ssdp-positive cells using the Ssdp[2082-G4] driver. We have included the data in a S10 Fig, in our revised manuscript. Our findings show partial rescue of the observed behavioral phenotypes upon knockdown of *Ssdp* in Ssdp-positive cells, suggesting a role for *Ssdp* in regulating behaviors. We provide a detailed description of these results in our revised manuscript (lines 518-538.

10) Page 7 and Fig. 5. Additional characterization of the influence of Ssdp on the head is needed. How does Ssdp overexpression influence the head? Is the eye, antenna or proboscis affected. Light microscope images of the Drosophila head should be a better way to show the connection between Ssdp and head development. These data would help readers understand the full scope of the findings.

>> We appreciate the reviewers' comment. We have performed additional characterization of the influence of *Ssdp* on the body of Drosophila. We have examined the effects of *Ssdp* overexpression on the wings and head morphology, including the eye, antenna, and proboscis, using scanning electron microscopy (SEM) (Fig 3). We have included images of the wings and Drosophila head obtained using SEM in our revised manuscript, along with descriptions of the observed defects in wings, antenna, and head size. These data provide a more comprehensive understanding of the impact of *Ssdp* on wings and head development and morphology (lines 160-182).

11) Genotype of RNA sequencing: Why did the authors decide to only focus on the overexpression of Ssdp and not on its knockdown? Please clarify this section.

>> We appreciate the reviewers' comment. The decision to focus on the overexpression of Ssdp rather than its knockdown in our RNA sequencing experiments was based on the similarity of phenotypes observed between *Ssdp* overexpression and knockdown. We found that both *Ssdp* overexpression and knockdown resulted in similar behavioral and morphological phenotypes in *Drosophila*. Therefore, we chose to use *Ssdp* overexpression as a tool to identify molecular changes associated with altered *Ssdp* levels in our RNA sequencing experiments. We have clarified this in the manuscript to provide a better understanding of our experimental approach.

13) Fig. 6: RNA sequencing was performed on RNA extracted from heads which include eye and cuticle. Since the requirement of SsdP (as can be noticed by its level of expression in the FlyAtlas database), verification of the list of main differentially expressed genes via the Quantitative Real-time RNA PCR extracted from the brain (not from the head) is necessary. This verification also concerns Fig.7C.

>> We appreciate the reviewers' comment. In the revised version, we have included validation of multiple genes using RNA extracted specifically from the brains, rather than the heads, to verify the list of main differentially expressed genes identified in our RNA sequencing experiments (Fig 7I and S7 Fig). The results showed consistent upregulation and downregulation of genes in the brain, consistent with the findings from RNA sequencing (lines 381-386) as well as the dysregulated fission/fusion genes (lines 412-413). We have included this additional validation data in the revised version of the manuscript to further support our findings.

14) Page 13 and Fig. 8. Does a knockdown of Ssdp in sensory neurons affect the sensory perception and habituation phenotypes? Even if these experiments may seem outside the scope of the paper, discussing them at a minimal would be critical for the reader to understand the potential novelty of these results.

>>We appreciate the reviewers' comment. While investigating the effects of Ssdp knockdown in sensory neurons on sensory perception and habituation phenotypes would be interesting, it is beyond the scope of the current study, which primarily focuses on the role of Ssdp in neural development and behavior. However, we have included a discussion in the revised version of the manuscript, mentioning the potential novelty of these results and acknowledging that these experiments were not conducted in this study (lines 626-629).

Minor

15) Information on the genotype of the lines used in this work are not clear. Maybe detail the full genotype in the legends.

>>We appreciate the reviewers' comment. In the revised version, we have provided detailed information on the genotypes of the lines used in this work in the figure legends.

16) Fig.1. Alignment analysis: Further description of the method and tools/platforms in the text, or methods is needed.

>>We thank the reviewers' for their comment. We have included a detailed description of the alignment method, including the tools/platforms used, in the Methods section of the revised version of the manuscript (lines 779-786).

17) How old are the adult flies used in this study for the molecular, cellular and behavioral assays?

>>We thanks the reviewers for their comment. In the revised version of the manuscript, we have included the age of adult flies used in all section where the molecular, cellular, and behavioral assays and associated results have been listed.

Reviewer #2: The manuscript is focused on using Drosophila as a tool to study the role of genes located in regions affected by microdeletion/duplication in autism spectrum disorders (ASD) in the pathogenesis of these neurodevelopmental disorders. Changes in the dosage of these genes might be involved in these diseases but the pathogenic mechanisms are still unknown.  In particular, the authors focused on SSBP3, Ssdp in the fruit fly ,and used both a knockdown and an overexpression strategy to assess the role of CNVs in neurodevelopment and autism-associated behaviour.  The authors performed an interesting analysis of the consequences of altering Ssdp levels on different aspect of the animal biology, ranging from tissue development to gene expression alteration, to behavior. Overall, the conclusions are interesting and novel, nevertheless the neurodevelopment analysis is rather superficial and would require a more detailed analysis. In conclusion, I think this paper is a good

candidate for Plos Biology but I would like to see the Major points addressed before considering it for publication.

>> We appreciate the reviewer's positive feedback on our manuscript and their recognition of the interesting and novel findings presented in our study. We agree with the reviewer's comment on the need for a more detailed neurodevelopmental analysis. We have carefully considered the reviewer's comments and have addressed them in our revised manuscript. Specifically, we have provided additional details and analysis on neurodevelopmental aspects, including brain development, and behavioral alterations associated with Ssdp manipulation during larval stage [overexpression (lines 198-203 and lines 215-218, Fig 4A, G) and knockdown (lines 239-241 and lines 245-247, Fig 5B, E)]. We believe that these additions have strengthened our study and addressed the concerns raised by the reviewer. We hope that these revisions adequately address the concerns and make our manuscript suitable for publication in PLOS Biology.

Major points.

-  	The authors analyzed SSBP3 expression in human brain using already available scRNA seq data and conclude that it colocalizes with SLC17A7, CUX2, RORB and oligodendrocytes. Even though the heatmap shown in Figure 2A is quite convincing, the scatter plots (Figure 2B) are not. SSBP3 seems to be expressed at very low levels (if the color scale indicates level of expression, which is not mentioned in the text nor in the figure legend) in most of the domains where SLC17A7 is expressed and the same is true for RORB. Also, in the oligodendrocyte cluster there seem to be some small areas with higher expression of SSBP3 but the majority show very low expression levels. To confirm the colocalization in the different clusters of cells a better analysis is needed, maybe highlighting clearly which area the authors refer to when they speak of colocalization.

>>We thank the reviewer for their comment and feedback on our analysis of SSBP3 expression in human brain using scRNA-seq data. In the revised version of our manuscript, we have included a new panel (Fig 2B) that highlight the colocalization of SSBP3 with different excitatory neuronal markers, addressing the concern about better visualizing the colocalization in different clusters of cells. We acknowledge that SSBP3 is expressed at lower levels compared to markers such as SLC17A7 and RORB, and we have now mentioned this in the revised manuscript for clarity (lines 124-125). Additionally, we have provided a list of cell clusters and highlighted those that colocalize with SSBP3, including the oligodendrocyte cluster (Fig 2B). We believe these revisions have improved the clarity and accuracy of our findings, and we appreciate the opportunity to address the reviewer's concerns.

-  	To investigate the role of Ssdp during development the authors measured the neuropil area by staining the brain with anti-DLG antibody (Figure 3A,B). The threshold feature was used on maximum intensity projection images and the central area of the brain was selected (from methods). I assume the quantification of the neuropil has then been performed on these images. -I think this is not a correct way of quantifying neuropil size in fact it does not take into account the flattening due to the mounting procedure. I do not think the authors used anything to normalize the quantification against this factor, except if all the brains quantified, both the controls and the Ssdp>GFP, were mounted on the same slide. The fact that this method is not the best can be also seen by the fact that the control brains in the overexpression and knowckdown experiments (Figure 3B and 4C) show significantly different neuropile areas, thing I do not think can be due to the genetic background.

>> We appreciate the reviewer's comment and feedback on our quantification of neuropil area in the Drosophila brain using anti-DLG antibody staining. In the revised version of our manuscript, we have made improvements to our methodology by incorporating volumetric analysis using 3D plugins in ImageJ. This allows us to account for flattening effects due to the mounting procedure and obtain more accurate measurements of neuropil size. We have also taken steps to ensure that the quantification is normalized appropriately, such as ensuring that all brains quantified, both the controls and the Ssdp>GFP, were mounted on the same slide. We believe these changes have improved the accuracy and reliability of our neuropil area measurements. These changes are available in the revised Fig 4 and 5 (lines 201-203, 239-243). Further, we have further validated these results using nc82 antibody which specifically label synapses (lines 193-195, Fig 4C, D).

-I think that a better evaluation of the neuropile area would come from the quantification of its volume, by considering all the stacks that compose the single image, since this would take into account the flattening. The analysis of neuronal area (Figure 3 C,D) is also very superficial and not properly performed. In the methods section it is indicated that the quantification of neuronal area has been performed in the same way as the neuropil area, nevertheless this is even less appropriate than in the case of anti-DLG antibody. Since Elav marks the nuclei of the neurons, performing the quantification of the neuronal area on a maximal projection is not correct, because it will not take correctly into account neurons present in different planes (in z) but with a similar x/y location. Moreover, if the Ssdp>GFP would affect neuronal size and neuronal number at the same time, i.e fewer bigger neurons, the area measurements would not be detecting such a defect.

>>We thank the reviewer for their insightful feedback on our quantification of neuropil and neuronal areas in the Drosophila brain. In the revised version of our manuscript, we have taken the reviewer's suggestions into account and made changes to our methodology accordingly (lines 838-850). We now perform volumetric analysis of all stacks, taking into consideration the flattening effects due to the mounting procedure, for both anti-DLG and anti-Elav images (S2 Fig A and Fig 5D) . This allows us to obtain more accurate measurements of neuropil and neuronal areas, accounting for neurons present in different planes in the z-axis but with similar x/y locations.

Finally, regarding the analysis of the Glia nuclei (Figure 3 E,F) also in this case the quantification is performed on a maximal intensity projection image. As clearly visible in Figure 3E w1118, there are areas where multiple nuclei are juxtaposed and overlapping, creating large positive areas, therefore I am wondering how this is taken into account when counting single nuclei. Did the authors used segmentation to be able to identify single nuclei in area such the one described? Otherwise, the quantifications are not accurate enough.

>> We appreciate the reviewer's comment on our analysis of glial nuclei. In the revised version of our manuscript, we have addressed this concern by performing 3D analysis of glial nuclei and implementing a segmentation approach to accurately identify single nuclei, even in areas where multiple nuclei are juxtaposed or overlapping. This allows us to obtain more precise quantification of glial nuclei, and we have updated our results and methods sections accordingly. The updated data is presented in Fig 4 and 5, lines 215-218, lines 245-247).

The same technical comments are valid for the experiments described in Figure 4.

In conclusion I think that for the paper to be considered for publication this part of the analysis (both the SsdpGal4 and the knockdown experiments) must be performed in better detail with appropriate controls: a) a quantification of the brain volume, rather than the brain area b) a quantification of the number of Elav positive cells and eventually size of cell bodies on the entire 3D of the brain c) a quantification of the glia cells in the 3D of the brain, based on all the single stack images, not on a maximal intensity projection.

>> We appreciate the reviewer's comment, we have performed re-analysis and included these results in the revised manuscript in the Fig 4 and 5, S2 Fig A.

-  	Regarding the expression of Ssdp in the brain, are the images showed in Figure 3G maximal intensity projection? Because if the authors want to investigate colocalization of the GFP with Elav or Repo it would be crucial to show single confocal planes or at least a maximal intensity projection of a number of confocal planes that covers only single layers of cell bodies.

>>Thank you for your feedback. In the revised version of our manuscript, we have included single confocal planes or maximal intensity projections of a limited number of confocal planes that cover only single layers of cell bodies in Fig 3G, to better investigate the colocalization of GFP with Elav or Repo. We have presented now this data in the S2 Fig A.

-  	The authors analyzed whether the neuro-anatomical defects are associated with defective Wnt signaling and in Figure 5C they claim that although Armadillo and Wingless immunofluorescence were reduced, they were not statistically significant.  Nevertheless, it seems that the pattern of expression of the two genes is slightly different in terms of number of positive stripes, could the authors comment?

>>Thank you for your observation. Upon re-examination of our data and further analysis, we have identified that Armadillo (Arm) expression was not statistically significant different at multiple regions, except for a downregulation in the middle pouch region (lines 278-280, Fig 6C). On the other hand, Wingless (Wg) expression was downregulated at most regions (lines 280-282, Fig 6D). Regarding number of strips as noticed by reviewer, we didnt find any statistical significant difference in number of armadillo-positive stripes but there was a significant increase in the number of wingless-positive stripes, and we have mentioned this in lines 282-282. We have updated our results accordingly in the revised version of our manuscript and included the revised data in Fig 6.

Minor points

-  	Relative to Figure 1: in the schematic representation of the genetic locations of the genes of interest is indicated the SSBP3-AS1 (antisense), but it is not mentioned in the text nor in the figure legend. If it's indicated because relevant to the study, it should be explained.

>> Thank you for bringing this to our attention. We apologize for the oversight. We have now included a brief mention of SSBP3-AS1 (antisense) in the revised manuscript to provide clarity on its relevance to the study (lines 98-99).

-  	The authors used SCOPE to determine the expression of Ssdp in relations to other genes: the colocalization with Repo and vGlut is clear but, since Ssdp expression seems to be much broader, I think it should be indicated as "partial" co-localization or as "Ssdp is expressed in Repo and vGlut positive cells.

>>Thank you for your suggestion. We have revised the text in the manuscript to indicate that Ssdp is partially co-localized with Repo and vGlut (lines 128-130).

-  	Relative to Figure 2: in the figure legend excitatory neurons are marked Exh and not Exc as in the scatter plot figure. Moreover, since the authors prove that the 2082Gal4, due to its nature, leads to increased mRNA levels of Ssdp, it would be important to mention if in the performed experiment the insertion is in homozygosis or in heterozygosis (either in the main text or in the figure legend).

>>Thank you for bringing this to our attention. We have revised the figure and its legend and it does not include those labels. Additionally, we have mentioned in the revised manuscript that we used heterozygous alleles of Ssdp^[2082-G4]^ flies, as homozygous flies are developmentally lethal (lines 154-155).

-  	Can you indicate in the figure legend or in the test the meaning of the dotted lines that circle some areas of the brain in Figure 3E?

>>We thanks reviewer for their comment. We have now removed dotted lines in the revised figure, dotted lines were highlighting loss of glial cells.

-  	In figure 5B I would suggest using a different layout for the dots in fact, since they are many and very close to each other, it becomes a bit crowded and confusing.

>>We have revised the figure, now labeled as 6B, we used a broken Y-axis to separate the dots and make it less crowded and more visually clear.

-  	In the figure legend of Figure 6I the p value and the number should be moved to the end of the sentence

>>Thank you for your feedback. We have made the necessary updates in the revised figure legend of Fig 6I and adjusted the p value and the number accordingly.

-  	In Figure 7A the UAS-Mito-Red distribution seems very different between the control and the other conditions, for example much more spread and diffuse in the Mushroom Body area of the control, is this due to the changes in size and shape of the mitochondria?

>>Thank you for bringing this to our attention. Yes, you are correct that the differences in the UAS-Mito-Red distribution between the control and other conditions in the revised Fig 8A could be attributed to differences in size and shape of the mitochondria as well as differences in the expression patterns of Elav-Gal4 and Ssdp-Gal4. In our analysis, we specifically zoomed in on similar regions such as SLP and SOG in both Elav-Gal4 and Ssdp-Gal4 samples to compare mitochondrial distribution. We have mentioned this in the revised manuscript to provide clarification (lines 402-406).

-  	In the Figure 7 legend it is indicated wich control has been used for normalization of RT-PCR while in other is not indicated.

>> We have now mentioned that Rpl32 was used as a normalization in all legends where RT-PCR data has been presented.

-  	In Figure 8J what is the meaning of the *? Please add an explanation in the figure legend.

>>	We thank reviewer for their comment, we have now mentioned meaning of * in the legend of revised Fig 9J (lines 462-464).

-  	In Supplementary Figure 6B it is not clear which line is the W1118-Ssdp-RNAi, indicated in light grey below the graph.

>>	We thank reviewer for their comment, we have now revised to reflect genotype clearly. Both the genotypic controls are presented jointly in the line graph.

-  	Figure 8H is not cited in the text

>> We thanks reviewer for pointing out the mistake, we have now cited it in text (now Fig 9H) (line 507).

-  	Figure 9 is cited incorrectly in the text, average speed is 9B, not 9A, wall following behaviour is 9C, not 9B.

>> We thanks reviewer for pointing out the mistake, we have corrected it in the revised version.
